# Aqueous ionic liquids redistribute local enzyme stability via long-range perturbation pathways

**DOI:** 10.1016/j.csbj.2021.07.001

**Published:** 2021-07-08

**Authors:** Till El Harrar, Benedikt Frieg, Mehdi D. Davari, Karl-Erich Jaeger, Ulrich Schwaneberg, Holger Gohlke

**Affiliations:** a Institute of Biotechnology, RWTH Aachen University, 52074 Aachen, Germany; b John-von-Neumann-Institute for Computing (NIC), Jülich Supercomputing Centre (JSC), Institute of Biological Information Processing (IBI-7: Structural Biochemistry), and Institute of Bio- and Geosciences (IBG-4: Bioinformatics), Forschungszentrum Jülich GmbH, 52428 Jülich, Germany; c Institute of Molecular Enzyme Technology, Heinrich Heine University Düsseldorf, 52428 Jülich, Germany; d Institute of Bio- and Geosciences IBG-1: Biotechnology, Forschungszentrum Jülich GmbH, 52428 Jülich, Germany; e Institute for Pharmaceutical and Medicinal Chemistry, Heinrich Heine University Düsseldorf, 40225 Düsseldorf, Germany; f DWI – Leibniz Institute for Interactive Materials e.V., 52074 Aachen, Germany

**Keywords:** Protein stability, Molecular dynamics simulations, Allostery, Biocatalysis, Ionic liquids, Protein engineering

## Abstract

Ionic liquids (IL) and aqueous ionic liquids (aIL) are attractive (co–)solvents for biocatalysis due to their unique properties. On the other hand, the incubation of enzymes in IL or aIL often reduces enzyme activity. Recent studies proposed various aIL-induced effects to explain the reduction, classified as direct effects, e.g., local dehydration or competitive inhibition, and indirect effects, e.g., structural perturbations or disturbed catalytic site integrity. However, the molecular origin of indirect effects has largely remained elusive. Here we show by multi-μs long molecular dynamics simulations, free energy computations, and rigidity analyses that aIL favorably interact with specific residues of *Bacillus subtilis* Lipase A (*Bs*LipA) and modify the local structural stability of this model enzyme by inducing long-range perturbations of noncovalent interactions. The perturbations percolate over neighboring residues and eventually affect the catalytic site and the buried protein core. Validation against a complete experimental site saturation mutagenesis library of *Bs*LipA (3620 variants) reveals that the residues of the perturbation pathways are distinguished sequence positions where substitutions highly likely yield significantly improved residual activity. Our results demonstrate that identifying these perturbation pathways and specific IL ion-residue interactions there effectively predicts focused variant libraries with improved aIL tolerance.

## Graphical abstract

**Figure:**
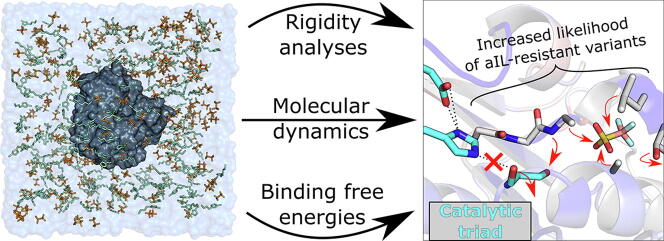

## 1 Introduction

Ionic liquids (IL) are organic salts that are liquid at low temperatures, often at room temperature [1]. Despite their early discovery in 1914, they gained attention in the last decades after discovering the first air- and moisture-stable IL [2], [3]. Their ionic nature grants them a wide range of interesting physicochemical characteristics for industry and ecology, such as strong ionic interactions, negligible vapor pressure, high solvation potential, and high thermal stability [4].

Notably, some of these properties change upon adding other molecular species such as water [5], [6], [7], [8], [9]. While this is beneficial in some cases, e.g., for the reduction of viscosity to increase molecular diffusion [10], [11], other effects such as a drastically decreased solvation potential can be limiting for aqueous IL (aIL) use [12], [13], [14]. Consequently, studying aIL has gained much attention and yielded numerous applications [4], e.g., as environmental-friendly alternatives for commonly used organic solvents in organic synthesis [1], [15], [16] or as (co–)solvents in biocatalysis [17], [18], [19], [20] and green industrial processes [14], [21], [22], [23], [24].

However, for biotechnological approaches that apply IL as co-solvents in enzymatic transformations, enzymes are needed that can tolerate these conditions since IL often reduce enzyme activity and stability [25], [26], [27], [28], [29]. A reduction may even be observed for concentrations as low as the remnants of IL found in regenerated cellulose [23], [30]. Interestingly, the trend of decreasing enzyme stability with increasing IL concentration does not apply to all cases, as stabilizing effects in highly concentrated aIL were observed in multiple studies [31], [32], [33], [34], highlighting the complexity of ion-water-protein interactions in aIL.

Several mechanisms of enzyme inactivation due to aIL have been suggested, such as I) competitive inhibition and blocking of substrate access [35], [36], II) (local) protein dehydration [37], [38], [39], III) structural changes [40], [41] and perturbations [29], IV) disturbed catalytic site integrity [29], [39], V) dampened protein dynamics [35], and VI) protein denaturation, e.g., induced by direct hydrophobic interactions of IL ions with the protein core [37], [42]. Remarkably, although I) and II) comprise direct, local observations whose origins and impacts can often be grasped immediately, III) – VI) relate to indirect effects governed by long-range and often cooperative influences. Particularly for the latter, the molecular basis of these inactivating effects remains mostly unknown. This has precluded to devise a general strategy to increase enzyme tolerance towards aIL, although specific approaches have been proposed [29], [36], [39], [43], [44], [45].

For the enzyme *Bacillus subtilis* Lipase A (*Bs*LipA), a unique, complete site-saturation mutagenesis (SSM) library is available that covers all 3620 potential single substitutions with natural amino acids (181 substitution sites with 20 possible substitutions at each site) [26], which is available to us to use as experimental validation. *Bs*LipA is a small lipase, and the fold does not contain a lid domain [46]. It has often been used as a model system in similar experimental and computational studies [26], [36], [39], [41], [44], [47], [48], [49], [50], [51], [52], [53], [54], [55]. Furthermore, high-resolution X-ray structures (PDB-IDs 1I6W [46] & 1ISP [56]) are available. This library has been tested for tolerance against four commonly used aIL (1-butyl-3-methylimidazolium bromide ([BMIM/Br]), 1-butyl-3-methylimidazolium chloride ([BMIM/Cl]), 1-butyl-3-methylimidazolium iodide ([BMIM/I]) and 1-butyl-3-methylimidazolium trifluoromethanesulfonate ([BMIM/TfO])) and was used to support the hypothesis that introducing charged substitutions at the enzyme surface improves tolerance [26]. The concentration of the individual aIL was adjusted to result in residual activities of 30–40% with respect to the activity in buffer to allow for relative comparisons between the aIL [26]. For these reasons, *Bs*LipA was chosen as a model enzyme in this study. Furthermore, analysis of this library revealed that polar substitutions at buried positions are beneficial for tolerance and that substitutions to chemically different amino acids are most likely beneficial [26]. A mutagenesis study investigating resistant and non-resistant variants of *Bs*LipA in aIL further highlighted the wide range of effects aIL have on *Bs*LipA, which subsequently can be exploited to increase tolerance to IL and aIL via substitutions [36]. Yet, the study also indicated the complexity of finding the optimal substitution for each substitution site since the effects are often site-specific.

To gain insights into the molecular mechanisms underlying enzyme inactivation due to aIL, which can subsequently be used to propose beneficial substitutions, we investigate ion-protein interactions of the four aIL used to screen the SSM library following experimental conditions and salt solutions ([Na/Cl], [Na/I], and [K/Cl]) with *Bs*LipA by all-atom molecular dynamics (MD) simulations of in total 130 μs length and subsequent analyses of structural stability (see Table 5 for details on simulation systems and used IL and salt concentrations). Our MD simulations are the most extensive computations of enzyme-aIL interactions and aIL influences on global and local protein structural dynamics to date. Including salt solutions fits well in the context of “green solvents” since the use of abundantly available seawater was shown to be a viable alternative to freshwater for use in enzymatic hydrolysis of IL-pretreated lignocellulosic biomass [57]. Additionally, it enables us to expose ion-specific effects, particularly for the four different anions, as well as the effects of increased ionic strengths.

As an outstanding result, we present a potentially underestimated, indirect mechanism of aIL-induced enzyme inhibition. It originates from specific aIL interaction sites on the enzyme surface and disturbs the integrity of the core enzyme structure via percolation effects along pathways of perturbed interactions that underlie changes in local structural stability. This result provides a so far overlooked foundation to guide the rational design of enzyme variants with improved aIL resistance.

## 2 Results

### 2.1 Validation of the simulation setup and length

To investigate whether the chosen force field/water model and partial charge combination for aIL adequately reproduce experimental data, we computed the system density (Fig. S1A) and the self-diffusion coefficient (Fig. S1B) for aqueous [BMIM/Br] and [BMIM/Cl] solutions at different concentrations and multiple water model/partial charge combinations (see Table 6 for the used validation systems). The systems were set up as described in Section 4.1. All investigated force field and water model combinations reproduce the system density well and show transport properties in the same order of magnitude as experimental values. In particular, our results show that the non-polarizable general amber force field (GAFF) [58] in connection with the OPC water model [59] predict physicochemical equilibrium and transport properties of the tested aIL in quantitative agreement with experimental data, without the need for charge scaling [60]. This allows us to exploit the higher computational efficiency of non-polarizable force fields *versus* polarizable ones for studying the effects of aIL on proteins. For details on the analysis, see Text S1 in the SI.

**Fig. 1:**
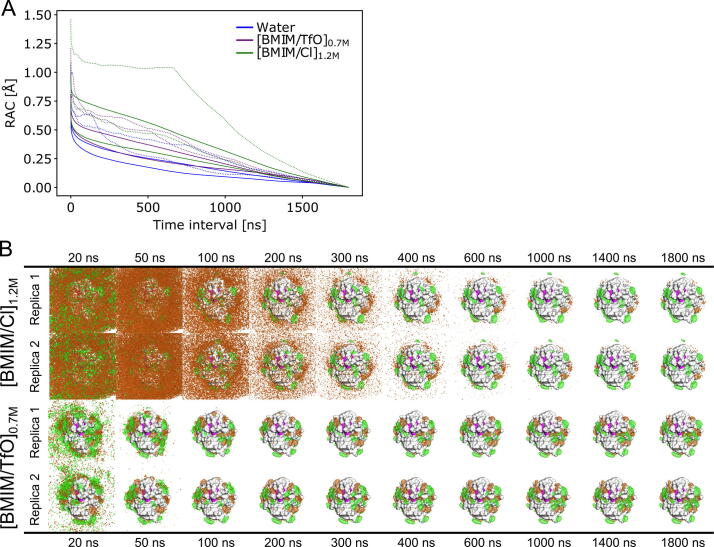
Influence of the simulation length on equilibrium properties of *Bs*LipA and IL ions. (A) RMS average correlation (RAC) curves for C_α_ atoms of *Bs*LipA from two replicas of MD simulations in water (blue), 1.2 M [BMIM/Cl] (green), and 0.7 M [BMIM/TfO] (purple). An offset of 10 frames, i.e., 2 ns, was used. The C_α_ atoms of each snapshot were superimposed to either the average structure over the entire trajectory (solid line) or the first averaged structure (time 0 - *τ*) (dotted line). (B) Density grids are shown for two replicas of *Bs*LipA in 1.2 M [BMIM/Cl] (upper panel) and 0.7 M [BMIM/TfO] (lower panel) after 20 ns, 50 ns, 100 ns, 200 ns, 300 ns, 400 ns, 500 ns, 600 ns, 1000 ns, 1400 ns, and 1800 ns. All distributions were normalized according to the number of frames, which is equivalent to the simulation time. Areas with a high density of [BMIM^+^] are shown as green meshes and areas for [Cl^–^] or [TfO^–^] ions as orange meshes, respectively. The purple patches show the partial surface area of the catalytic site residues. Densities of water are removed for clarity. *σ*-values of 0.04 for [BMIM^+^], 0.02 for [TfO^−^], and 0.0025 for [Cl^–^] were used. (For interpretation of the references to color in this figure legend, the reader is referred to the web version of this article.)

Next, we addressed the convergence of our MD simulations by determining convergence rates of quantities that probe the extent of conformational sampling of *Bs*LipA and the dynamical properties of IL and salt ions around the protein. The RMS average correlation (RAC) [61] was used as a measure of the overall structural convergence of *Bs*LipA at different time intervals *τ* within a single trajectory (Fig. 1A). As expected, the RAC curves obtained by fitting against the average structure over the entire trajectory result in smooth curves with deviations decaying to < 1 Å within a few ns, suggesting the absence of overall structural changes. By contrast, when fitting against the first averaged structure, the bumps in the curves expose the timescales of local structural changes, e.g., caused by a small conformational change of a flexible loop region in the RAC curve of the slowest converging replica in 1.2 M [BMIM/Cl] (Fig. 1A). Here, generally, simulation times of ∼ 750 to 1250 ns are required to reach the RAC curves obtained by fitting against the average structure over the entire trajectory. Overall, these results suggest that, despite the absence of large structural changes in *Bs*LipA, simulation lengths in the order of microseconds are required to obtain converged local structural dynamics of *Bs*LipA in the investigated aIL. Such converged local structural dynamics of *Bs*LipA are a prerequisite to obtaining converged protein-ion interactions.

Finally, to probe for the convergence of ion distributions around *Bs*LipA, we analyzed the time evolution of the distributions over the course of the trajectories at defined time steps (Fig. 1B; see Fig. S2A–D for complete data of all investigated aIL and salt solutions). The results demonstrate that, across all systems, at least 600 ns of simulation time are required to obtain defined spatial distributions of the ions around the protein. More specifically, alkali metal and halogen ions need longer to form defined spatial distributions than the organic ions [BMIM^+^] and [TfO^–^], suggesting that the latter have a higher affinity towards the protein (Fig. S2A–D).

To conclude, our results suggest that the length of our MD simulations of 1.8 μs is sufficient to obtain converged interactions of aIL and salt solutions with *Bs*LipA.

### 2.2 IL ions replace water molecules from the *Bs*LipA surface at specific binding sites

**Fig. 2:**
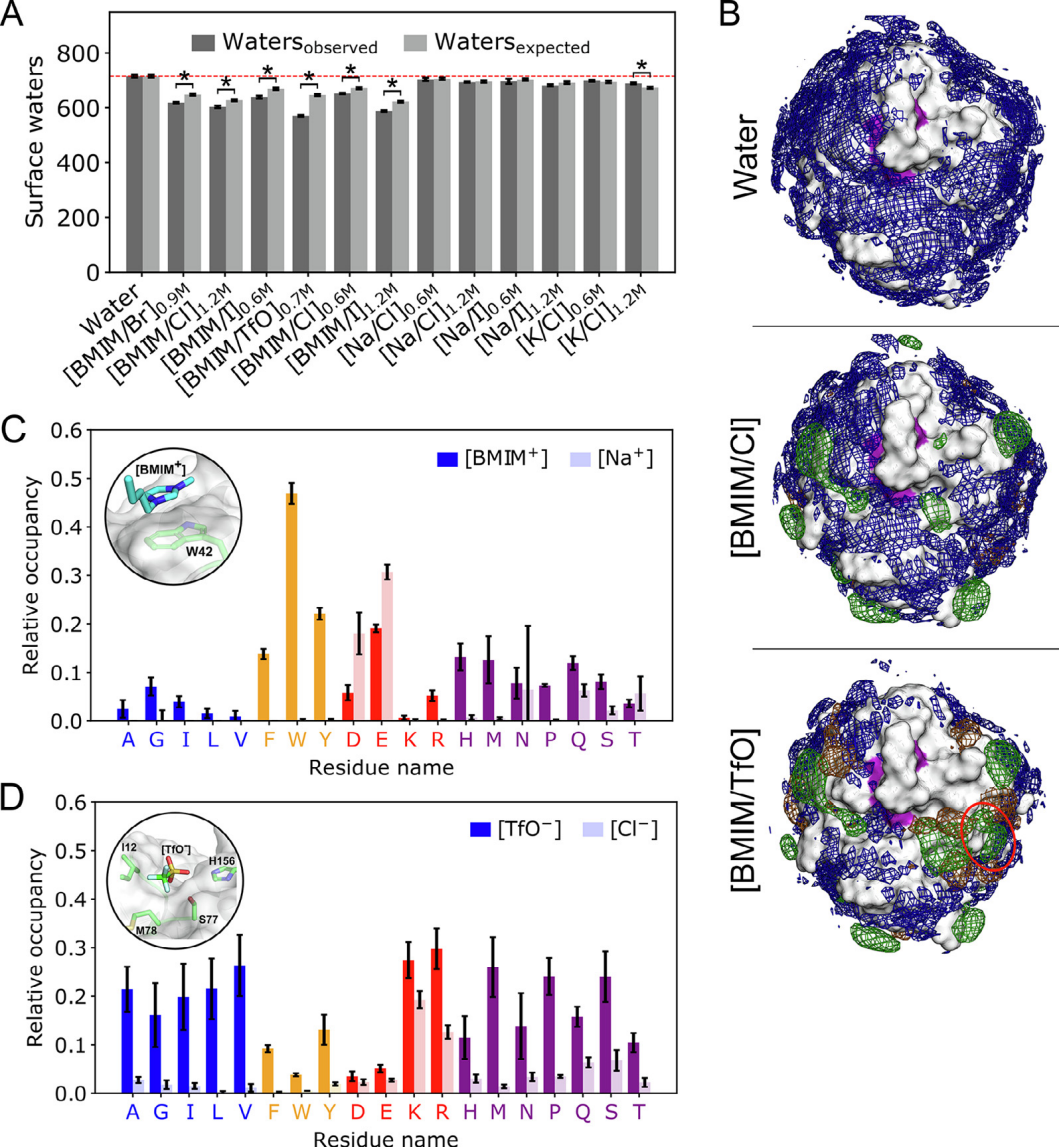
IL ions influence the number of water molecules in the first hydration shell at specific interaction sites. (A) Observed (dark grey) and expected (light grey, see Eq. (1)) number of water molecules around the *Bs*LipA surface. The red line denotes the value for *Bs*LipA in water. Significant differences (*p* ≤ 0.05, two-sided independent Student’s *t*-test) of the observed values with respect to the expected ones are marked with an asterisk. (B) Spatial distribution of solvent molecules around *Bs*LipA in water, 1.2 M [BMIM/Cl], and 0.7 M [BMIM/TfO] obtained from five replicas each. Regions with a high density of water, [BMIM^+^], or [TfO^–^]/[Cl^−^] molecules throughout the MD simulations are shown as blue, green, or orange meshes, respectively. The purple patches show the exposed surface of the catalytic site residues. A unique interaction site of [BMIM^+^] in [BMIM/TfO] is highlighted with a red circle. All distributions were normalized according to the number of frames. *σ*-values defining the intensity cutoff of the represented data of 0.04 for [BMIM^+^], 0.02 for [TfO^–^], and 0.0025 for [Cl^−^] were used. The *σ*-values of water were additionally adjusted using the respective *F_i_*. (C) Average residue-wise interaction frequency of cations with *Bs*LipA surface residues, exemplarily shown for 1.2 M [BMIM/Cl] (saturated bars) and 1.2 M [Na/Cl] (shaded bars). A representative interaction mode of [BMIM^+^] at the highest occupied residue W42 is shown. (D) Average residue-wise interaction frequency of anions with *Bs*LipA surface residues, exemplarily shown for 1.2 M [BMIM/Cl] (shaded bars) and 0.7 M [BMIM/TfO] (saturated bars). Note the different concentrations for this case. A representative interaction mode of [TfO^−^] at the catalytic site is shown. Residues involved in [TfO^−^] binding are depicted as sticks. Results of all graphs are shown as mean ± standard error of the mean (*n* = 5). Residues in panels C and D are grouped into aliphatic (blue), aromatic (yellow), charged (red), and polar (purple). (For interpretation of the references to color in this figure legend, the reader is referred to the web version of this article.)

To probe for the influence of protein-ion interactions on the hydration state of *Bs*LipA, we computed the mean number of water molecules at a distance < 3.4 Å to the protein surface throughout the trajectories (Fig. 2A). This distance relates to the first hydration shell around the protein [62]. According to the raw values, the number of water molecules decreases when ions are present; the decrease is more pronounced when systems contain bulkier IL ions. Notably, however, a significant and good correlation between the number of water molecules in the simulation box and the number of water molecules in the first hydration shell is found across all systems (*R*^2^ = 0.93, *p* < 0.001, Fig. S3). This finding suggests that the reduced water concentration in the aIL and salt systems is a dominant factor in the observed reduction of surface waters. To compensate for this effect, we computed a correction factor *F_i_* (Eq. (1)), which describes the relative amount of water molecules in the system compared to a simulation in pure water. Applying *F_i_* to the number of surface waters from the simulation in pure water yields the expected amount of surface waters for a given aIL and salt system; this amount is a system-specific reference value in the absence of protein-ion interactions (Fig. 2A). When comparing the raw values against these reference values, significant decreases in the number of surface waters due to protein-ion interactions are found for all aIL. The most pronounced decrease to 90% is found for [BMIM/TfO]. These results indicate that organic ions show preferential interactions with the protein that go beyond pure concentration effects. Our results thereby corroborate observations in previous studies, in which no correction for pure concentration effects was applied [36], [39]. Still, the lack of a correction likely leads to an overestimation of the observed protein-IL ion interactions in these studies [36], [39].

Density maps of all solvent molecules from five replicas of MD simulations each of *Bs*LipA in aIL and salt systems (Fig. 2B, exemplarily shown for 1.2 M [BMIM/Cl] and 0.7 M [BMIM/TfO]; see Fig. S4 for the remaining systems) reveal multiple specific interaction sites on the protein surface, particularly for [BMIM^+^] and [TfO^−^] ions. By contrast, inorganic alkali metal cations and halogen anions show only a few regions with increased density. Increasing IL ion concentrations led to the further occupation of regions populated already at low concentrations but also additional occupied regions (Fig. S4), indicating interaction sites with different affinities. A unique interaction site located next to the active site cleft of [BMIM^+^] ions is found for [BMIM/TfO], which suggests a counterion-specific promotion of [BMIM^+^] binding due to the presence of [TfO^–^] (indicated by a red circle in Fig. 2B; Fig. S5A), resulting in a significant increase of [BMIM^+^] molecules around the catalytic site (Fig. S5B) (see Text S3 in SI for more details). Adjusting the *σ*-cutoff of water, which defines the intensity cutoff of the represented data, with the *F_i_* values suggests that organic IL ions replace water molecules at binding regions more strongly than inorganic ions, which more markedly decreases protein hydration. In contrast to ref. [39], which described interactions of [BMIM^+^] and [TfO^–^] with similar regions of *Bs*LipA, we observed distinct interaction sites for either ion. This indicates that long simulation times are needed to accurately describe protein-ion interactions and their subsequent effects on solvent properties such as binding frequencies [55], [63], the spatial or radial distribution functions of solvent molecules [36], [38], [39], [43], and protein hydration [36], [39]. Such timescales are also needed to reveal coupled effects such as the counterion-induced promotion of [BMIM^+^] binding in [BMIM/TfO].

Next, we computed the relative occupancy of ions at all *Bs*LipA residues with bound/unbound states defined based on ion-specific distance criteria deduced from radial distribution functions (Fig. S6; see Section 4.6 for details). [BMIM^+^] ions predominantly interact with aromatic surface residues of *Bs*LipA via π-stacking and π-cation interactions. W42 shows the highest occupancy of up to 71% in 1.2 M [BMIM/Cl]. [Na^+^] and [K^+^] show a different interaction pattern, with the most frequent interactions formed to negatively charged surface residues via electrostatic interactions (average occupancies of 18% and 32% for aspartate and glutamate to [Na^+^]). As to anions, [TfO^−^] binds strongly to a wide range of residues, including aliphatic, polar, and charged ones, with positively charged residues showing the highest occupancy (59% for K23 in 0.7 M [BMIM/TfO]). Halogen anions showed lower binding frequencies of 13–21% for binding to charged residues. Note that the extensive simulation times used here allowed computing binding frequencies and interaction patterns for sufficiently long timescales to reach convergence for most interactions (see also next paragraph). By contrast, simulation times as long as 500 ns used previously were insufficient to yield converged results even for the strongest binding sites of [BMIM^+^] [55].

From the proportions of bound and unbound ions, we computed dissociation constants *K*_D_ (Eq. (2)) and binding free energies Δ*G*^0^ (Eq. (3)) for each ion to every surface residue of *Bs*LipA. Results are shown for selected, representative interactions of IL ions to *Bs*LipA surface residues in Table 1 (for the complete data of all Δ*G*^0^ values, see Tables S1 and S2 in the SI). The number of binding and unbinding events throughout the trajectories across all individual replica, according to the above distance criteria, is between 4,658–83,781 for cations and 19,053–104,901 for anions, respectively (Fig. S7). These results indicate that the computed *K*_D_ and Δ*G^0^* values are not compromised by kinetic trapping effects. Furthermore, the time evolution of selected Δ*G*^0^ demonstrates that the values are converged after simulation times of ∼ 100 ns for favorable residue/ion interactions and up to 600 ns for disfavorable ones (Fig. S8). For all inorganic ions, interactions with surface residues are energetically unfavorable at a 1 M standard state (Tables S1 and S2), which is in quantitative agreement with observations from ref. [64]. Only for [BMIM^+^] and [TfO^–^], binding to individual surface residues was energetically favorable considering a 1 M standard state, e.g., for interactions with W42 and K23, respectively (in 0.7 M [BMIM/TfO]: Δ*G*^0^ = –0.50 ± 0.03 kcal mol^−1^ and –0.44 kcal mol^−1^ ± 0.04 kcal mol^−1^ (mean ± SEM) (Table S1/Table S2)). However, averaging over all exposed aromatic or all exposed negatively charged residues for [BMIM^+^], or all exposed positively charged residues for [TfO^−^], yielded positive Δ*G*^0^ (Table 1).

**Table 1:** Thermodynamics of selected ion-*Bs*LipA residue interactions. [a]

Residue	Ion	Δ*G*^0^_calc._[b]	Δ*G*^0^_Literature_[c]	Comments for literature values
W/F/Y	[BMIM^+^]	0.77 ± 0.38 (*n* = 12)	–	–
D/E	[BMIM^+^]	1.68 ± 0.15 (*n* = 11)	–	–
D/E	[Na^+^]	1.16 ± 0.15 (*n* = 11)	1.38 ± 0.25 (*n* = 15)	Mean of all D/E in S6 of bacterial ribosome in 0.133 M [Na/Cl] [64]
K/R	[TfO^–^]	0.49 ± 0.10 (*n* = 15)	–	–
K/R	[Cl^−^]	1.30 ± 0.08 (*n* = 15)	1.26 ± 0.25 (*n* = 16)	Mean of all K/R in S6 of bacterial ribosome in 0.133 M [Na/Cl] [64]

[a] For a complete list, see Tables S1 and S2 in the SI. The selected residues are those for which most preferential interactions with ions are found according to Fig. 2C and D.

[b] Results from 0.6 M [Na/Cl] or 0.7 M [BMIM/TfO] according to Eqs. (2), (3); averages are computed over all exposed residues; in kcal mol^−1^; for 1 M standard state.

[c] In kcal mol^−1^.

To conclude, our results demonstrate that IL ions show preferential interactions with *Bs*LipA that go beyond pure concentration effects and that IL ions replace water molecules at binding regions more strongly than inorganic ions do. On average, at a 1 M standard state, Δ*G*^0^ of IL ion binding to residues are positive, as are those of inorganic ions. Still, more preferred residue types can be identified (in particular, aromatic or negatively charged ones for [BMIM^+^] or positively charged ones for [TfO^−^]). For interactions to specific residues negative Δ*G*^0^ are found, such as to W42 for [BMIM^+^] or K23 for [TfO^−^].

### 2.3 Incubation of *Bs*LipA in aIL does not influence global protein properties

**Fig. 3:**
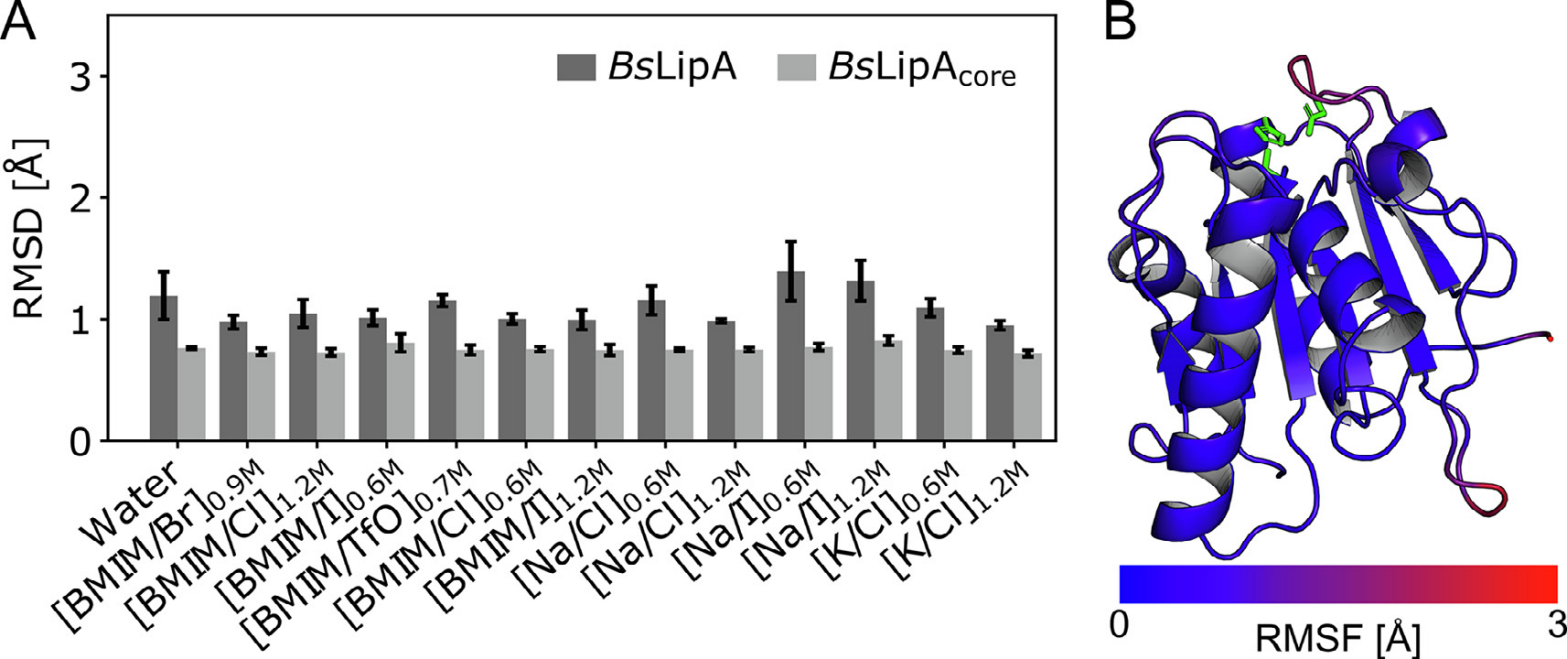
The global structure and dynamics of *Bs*LipA do not change in aIL. (A) RMSD of *Bs*LipA backbone atoms to the crystal structure (PDB-ID 1I6W). *Bs*LipA shows an average RMSD < 1.5 Å for all solvents (dark grey bars). Excluding two flexible loops (residues 118–121 & 131–137) reduces the RMSD to ∼ 0.8 Å in all solvents (termed *Bs*LipA_core_, light grey bars). Data is shown as mean ± standard error of the mean (*n* = 5). No significant differences (*p* ≤ 0.05, two-sided independent Student’s *t*-test) to the results in pure water were observed. (B) Residue-wise backbone RMSF of *Bs*LipA in water. The RMSF is mapped onto the *Bs*LipA crystal structure (see color scale). The catalytic site residues are depicted as green sticks. Several loops show increased mobility. Similar RMSF values and distributions are found for all solvents (Fig. S9). (For interpretation of the references to color in this figure legend, the reader is referred to the web version of this article.)

We next investigated the effects of IL and inorganic ions on *Bs*LipA structure and dynamics. The root-mean-square deviation (RMSD) of backbone atoms, a measure for structural similarity with respect to the crystal structure, is < 1.5 Å in all solvents and statistically not significantly different from MD simulations in pure water (Fig. 3A). Excluding two flexible loops (residues 118–121 & 131–137) reduces the RMSD to ∼ 0.8 Å in all cases.

The residue-wise root-mean-square fluctuations (RMSF) of backbone atoms, a measure for atomic mobility, revealed only a few regions with values > 1.0 Å (Fig. 3B); these results are mostly consistent across all solvents (Fig. S9). The more mobile regions contain single solvent-exposed aromatic and charged surface residues or loops consisting of residues 118–121 and 131–137. Furthermore, it has recently been suggested that aqueous ILs may induce enzyme aggregation, e.g., as a result of local fluctuations in enzyme structure that result in increased exposure of hydrophobic protein residues [27], [35]. Therefore, we computed the average hydrophobic surface area of *Bs*LipA in water and aIL or salt solutions (Fig. S10) of all residues with a hydropathy > 0 according to the Kyte & Doolittle hydropathy index [65]. However, we did not observe significant increases between water and aIL or salt solutions. Finally, other descriptors of the global protein structure, such as the overall solvent-accessible surface area (Fig. S11A), the average structure (Fig. S11B), or the radius of gyration (Fig. S11C), did not indicate pronounced changes in the global *Bs*LipA structure either. To conclude, *Bs*LipA does not show pronounced changes in structure or dynamics upon incubation in aIL, supporting our findings of only specific influences of aIL from the previous section.

### 2.4 aIL alter the local structural stability of *Bs*LipA

To investigate how specific interactions of IL ions with *Bs*LipA residues perturb the structural stability (rigidity and flexibility) of *Bs*LipA, we used the rigidity theory- and ensemble-based Constraint Network Analysis (CNA) [66] following ref. [67]. Here, thermal unfolding simulations were carried out on the ensemble of network topologies generated from MD snapshots and subsequently analyzed to obtain the neighbor stability map *rc_ij,_*_neighbor_ that displays at which stage of the unfolding simulation a rigid contact *R_i,j_* between two residues *i* and *j* at most 5 Å apart is lost; lower values of the cutoff energy *E*_cut_ display a higher local stability.

**Fig. 4:**
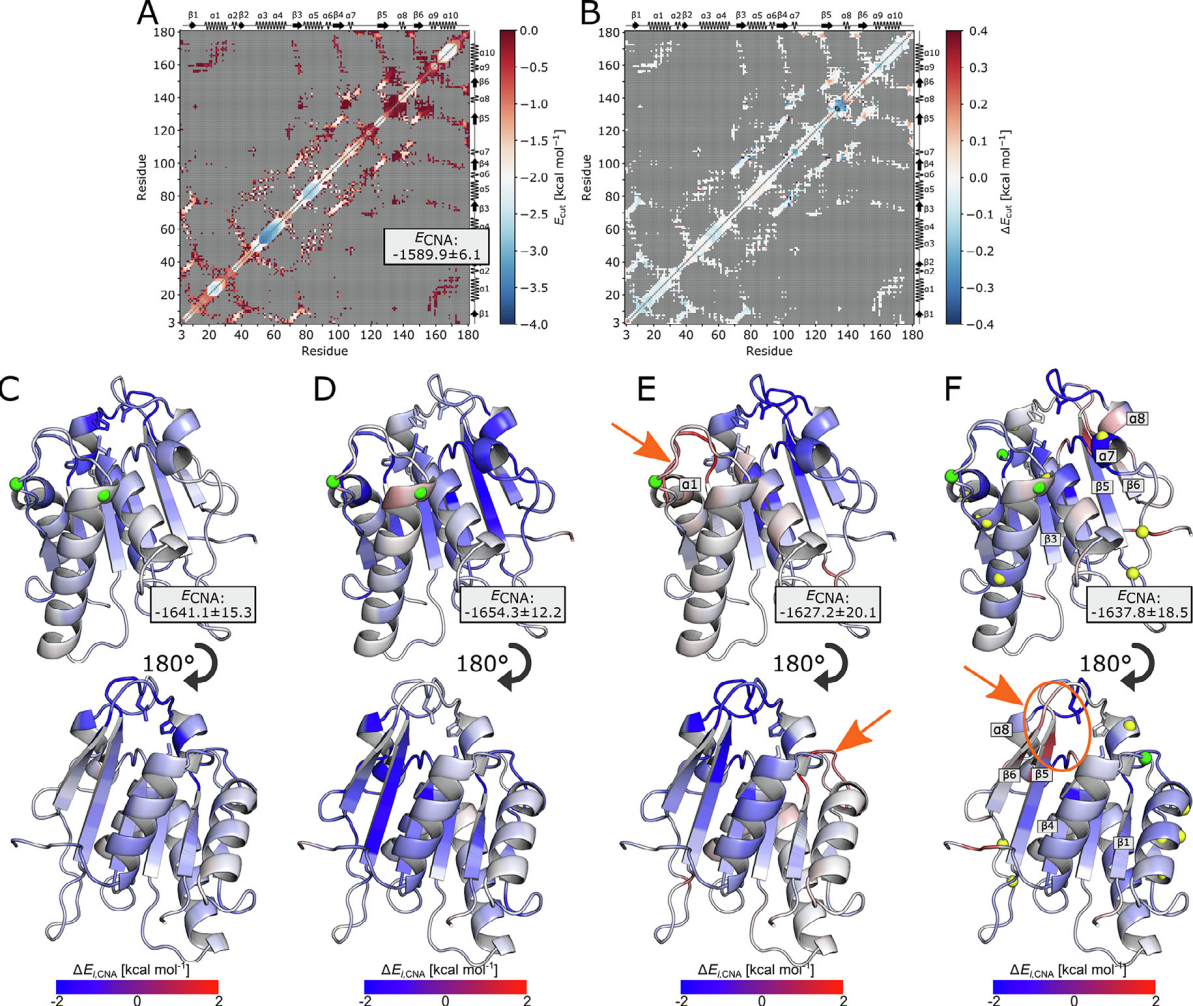
Incubation of *Bs*LipA in aIL induces changes in the local structural stability. (A) Neighbor stability maps *rc_ij,neighbor_* of *Bs*LipA in water. *E*_cut_ describes the energy when a rigid contact between two residues *R_i,j_* is lost during the thermal unfolding simulation with red (blue) colors denoting lower (higher) energies, thus representing less (more) stable contacts. Only contacts of residues that are at most 5 Å apart are considered; contacts with higher distances are shown in grey. (B) Δ*rc_ij,neighbor_* with respect to water, exemplarily shown for 0.7 M [BMIM/TfO] (see Fig. S12 for the remaining results), highlights changes in the protein stability upon incubation in aIL. Blue (red) color indicates that a contact is more (less) stabilized in aIL than in water. (C-F) Δ*E_i_*_,CNA_ (Eq. (4)) mapped onto the *Bs*LipA crystal structure for (C) 0.9 M [BMIM/Br], (D) 1.2 M [BMIM/Cl], (E) 0.6 M [BMIM/I], and (F) 0.7 M [BMIM/TfO]. Blue (red) color indicates that a contact is more (less) stabilized in aIL than in water. Data is shown as the mean over all replica (*n* = 5). Cation (anion) binding sites with Δ*G*^0^ < 0 kcal mol^−1^ are shown as green (yellow) spheres. Solvent-specifically destabilized residue patches are highlighted with arrows. For clarity, Δ*E_i_*_,CNA_ values were capped at ± 2 kcal mol^−1^. *E*_CNA_ values, representative for the global structural stability (see Eq. (5)), are presented as mean ± standard error of the mean (*n* = 5) over all replica. (For interpretation of the references to color in this figure legend, the reader is referred to the web version of this article.)

Analysis of the residue-wise extent of the structural stabilization and destabilization with regard to water, Δ*E_i_*_,CNA_ (Eq. (4)), showed that incubation of *Bs*LipA in aIL resulted in a marked redistribution of structural stability across the *Bs*LipA structure compared to incubation in water (Fig. 4A, B), with some regions becoming more and others less structurally stable (Fig. 4C-F; Fig. S12A–D). This finding is also reflected in the sum over all rigid contacts *E*_CNA_ (Eq. (5)), which has been used as a proxy for the melting enthalpy of a protein [68]. Here, *E*_CNA_ values with respect to the system in water differ by at most 4%, indicating similar global structural stabilities of *Bs*LipA in the aIL; the *E*_CNA_ values with respect to the system in water are lower, indicating that *Bs*LipA is globally slightly more rigid in aIL. Regions that were similarly affected in most aIL include the N-terminal residues H3-N4, the four central β-strands β1(V6-V9), β3(D72-A75), β4(N98-L102), β5(L124-Y129), the α-helices α7 (N106-T109), α10 (S163-N174) and the residues H10-F19 (including part of α1), H76-G79, and S130-Y139.

Notably, incubation in the aIL structurally stabilized most of these patches (see Fig. 4C–F). Such stabilization can arise from aIL-induced local conformational changes that increase or reinforce interactions between patch residues and their neighborhoods [69]. Residues 130–136 have a direct functional implication in that they contain the catalytic residue D133 [46]. Rigidification of this loop may influence substrate access or product egress or impact the correct localization of D133 for catalysis. As to destabilizing effects, only the N-terminal residues H3 and N4 were destabilized in most aIL (Fig. 4C–F). The destabilization of H3 (Δ*E_H3,_*_CNA_ = −0.07 to 0.39 kcal mol^−1^) and N4 (Δ*E_N4,_*_CNA_ = −0.12 to 0.51 kcal mol^−1^) is likely connected to the strong stabilization of K35 (Δ*E_K35,_*_CNA_ = −0.45 to −1.57 kcal mol^−1^) and its neighboring residues D34 (Δ*E_D34,_*_CNA_ = −0.24 to −0.61 kcal mol^−1^), L36 (Δ*E_L36,_*_CNA_ = −0.10 to −0.99 kcal mol^−1^), and Y37 (Δ*E_Y37,_*_CNA_ = –0.29 to –0.69 kcal mol^−1^), which arise from a redistribution of noncovalent interactions (see below). In [BMIM/I] and [BMIM/TfO], one additional major residue patch became destabilized (indicated by arrows in Fig. 4E/F). In [BMIM/I], this includes residues H10 to A20 and the neighboring residues M78, L160, and Y161 (Δ*E_i,_*_CNA_ values ranging from +0.16 to +2.29 kcal mol^−1^; Fig. 4E). Likely, the destabilization of the neighboring residues M78, L160, and Y161 was an indirect effect of the destabilization of residues H10-A20 caused by a structural reorganization. In [BMIM/TfO], this includes residues Y129, N138, Y139, L140, S141, Q150, and I151 (Δ*E_i,_*_CNA_ values ranging from −0.61 to +1.22 kcal mol^−1^) (Fig. 4F). Interestingly, the residues extended over multiple secondary structure elements (Q150 and I151 of β6, Y129 of β5, and N138-S141 of α8), including the buried residue S141 and the almost inaccessible residue Y129, indicating a long-range perturbation of local structural stability due to aIL.

To conclude, these results show that incubation in aIL leads to local decreases and increases of *Bs*LipA structural stability, but only to small global changes in structural stability, in line with the results from the previous section. Due to rigidity percolation [70], i.e., distant structural details can determine whether a protein region is flexible or rigid, such changes usually encompass multiple neighboring residues and can reach residues distant to residues on the surface interacting with IL ions.

### 2.5 IL ion interactions at specific binding sites lead to pathways of perturbed noncovalent interactions

**Fig. 5:**
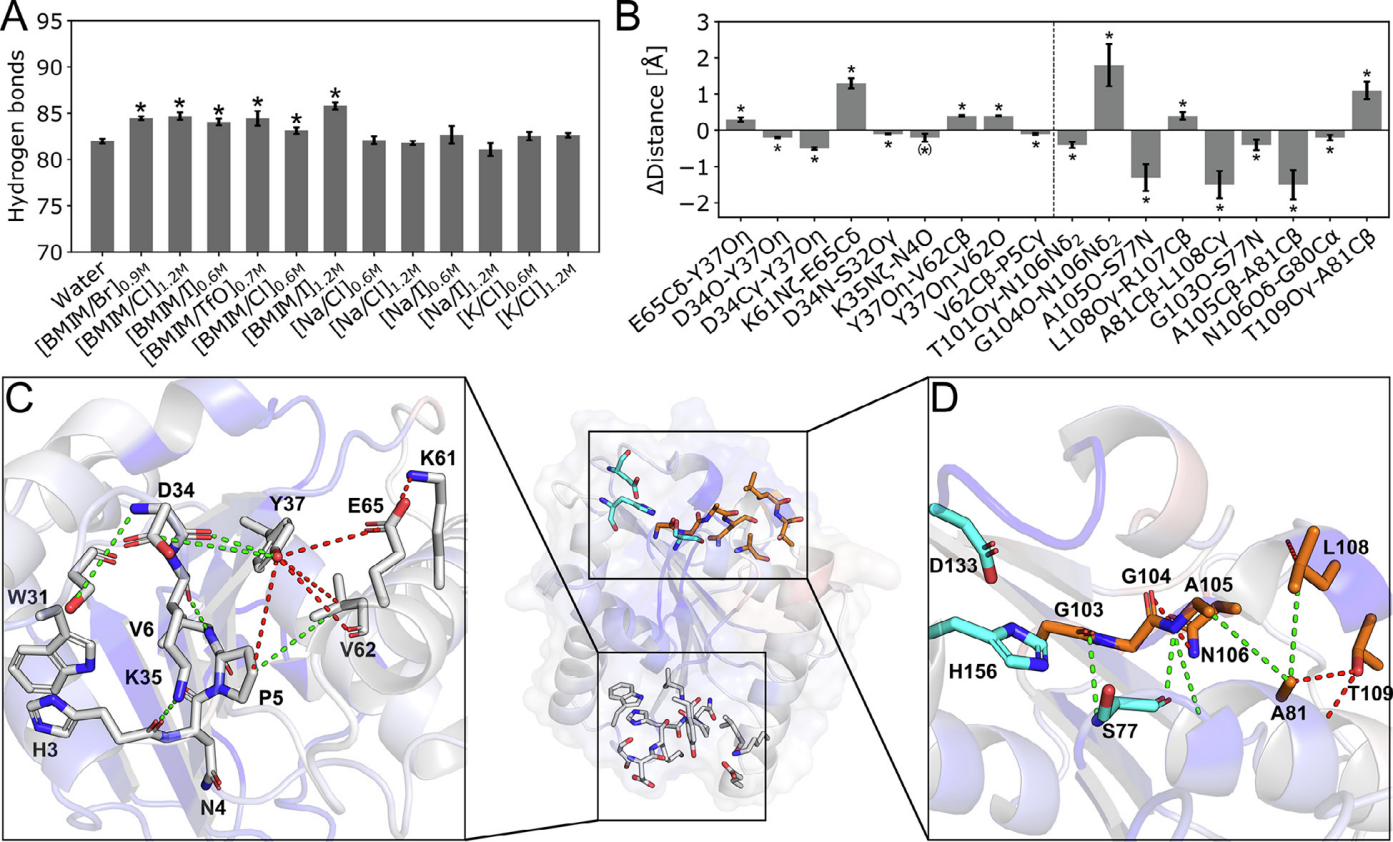
aIL introduce significant perturbations of the intramolecular hydrogen bond network through local interaction changes originating from surface residues. Values in graphs depict the mean ± standard error of the mean (*n* = 5). Significant differences (*p* ≤ 0.05, two-sided independent Student’s *t*-test) to the values from the water simulation are marked with an asterisk. (A) The average number of intramolecular hydrogen bonds of *Bs*LipA per frame. Solvents containing organic ions lead to a significant increase in the number of hydrogen bonds compared to water. (B) Average distance differences of interactions involved in PP1 (left from the dashed line) and PP2 (right from the dashed line) in 0.7 M [BMIM/TfO]. (C) A close-up-view of PP1 originating at residues D34, Y37, and E65 observed in all aIL. A weakened E65Oε-Y37Oη interaction led to a structural reorganization of multiple residues that further influenced adjacent residues. Interactions in PPs are colored green (red) if the distances are significantly smaller (greater) or the interaction is strengthened (weakened). The *Bs*LipA structure is colored according to Δ*E_i_*_,CNA_ values with blue (red) colors indicating more (less) stabilized residues. The catalytic site residues are depicted as cyan sticks. (D) A close-up-view of PP2 observed in [BMIM/TfO], originating from L108. The conformational change in L108 led to changes in interaction distances ranging from 0.1 Å to 1.8 Å showing a major structural reorganization. See caption for panel C for the color code of lines and the coloring of the *Bs*LipA structure according to Δ*E_i,CNA_*. (For interpretation of the references to color in this figure legend, the reader is referred to the web version of this article.)

To understand the origin of the changes in structural stability in aIL at the molecular level, we analyzed changes in noncovalent interactions between *Bs*LipA residues. Here, changes in hydrogen bonds (including salt bridges) are exemplarily described. In all aIL, *Bs*LipA showed a significantly increased average amount of intramolecular hydrogen bonds (*p* < 0.03 for all solvents, two-sided independent Student’s *t*-test) (Fig. 5A). None of the solvents containing only inorganic ions showed the same effect (*p* > 0.1 for all solvents, two-sided independent Student’s *t*-test). These results indicate that interactions of IL ions induce changes in the overall hydrogen bond network.

We then computed the changes in the average occurrence frequency for each H-bond with respect to water. Considering only H-bonds with absolute deviations > 5% resulted in a range of seven significantly (*p* < 0.05, two-sided independent Student’s *t*-test) altered interactions in 0.6 M [BMIM/Cl] and 0.6 M [K/Cl] to 33 in [BMIM/TfO]. The number of significantly altered interactions showed a significant and strong correlation with the concentration (*R*^2^ = 0.66, *p* = 0.02), suggesting that the interactions were influenced by IL ions (Fig. S13).

Further analyses revealed that these changes subsequently induced conformational changes that were transferred to adjacent residues in an attenuated manner via perturbation pathways (PP). As a result, we observed multiple neighboring residues of interaction sites of aIL that showed significantly altered hydrogen bond frequencies and interaction distances (Fig. 5A/B). Two of these interaction sites and their impacts on *Bs*LipA structure will be discussed now. These interaction sites are I) the E65Oε-Y37Oη-D34O interaction, which was significantly perturbed in all 12 solvents, and II) the residue patch around L108, which was significantly perturbed in 0.7 M [BMIM/TfO].

As to the first PP, a reorganization in multiple surface residues of *Bs*LipA in all solvents with D34, Y37, and E65 being the key contributors was observed (Fig. 5C; Table 2), which was reflected in altered interaction distances (Fig. 5A). E65 is a major interaction site for all cations with binding frequencies of up to 45%. Furthermore, Y37 has been experimentally verified as an [BMIM^+^] interaction site [55] and showed binding frequencies of > 22% for all solvents in our simulations. These interactions probably induce the structural reorganization of Y37 to D34 at the expense of the Y37-E65 interaction. The conformational changes lead to increased interactions of D34 with neighboring residues K35 and L36, which results in overall increased stability of the local protein structure around D34, and the subsequent destabilization of H3/N4 via long-range effects from K35, in line with our rigidity analysis (Fig. 4). This destabilization may lead to partial protein unfolding in aIL and, thus, reduce the observed activity of *Bs*LipA [26].

**Table 2:** Occurrence frequencies of H-bonds affected in PP1 in 1.2 M [BMIM/Cl] compared to water.

H-Bond	Water [a]	1.2 M [BMIM/Cl] [b]	Diff. [c]	*p*-value [d]	# of systems [e]	# of aIL [f]
D34O-Y37Oη	0.40	0.65	+0.25	<0.01	12/12	6/6
E65Oε-Y37Oη	0.19	0.05	−0.14	<0.01	12/12	6/6
K35O-V6N	0.59	0.67	+0.08	<0.01	7/12	5/6
H3O-K35Nη	0.26	0.38	+0.12	0.03	10/12	5/6

[a] Occurrence frequency of H-bonds in water.

[b] Occurrence frequency of H-bonds in 1.2 M [BMIM/Cl].

[c] Difference in the occurrence frequency between 1.2 M [BMIM/Cl] and water.

[d] *p*-value determined by a two-sided independent Student’s *t*-test.

[e] Proportion of systems (see Table 5 for a complete overview of all simulated systems) that showed significantly different occurrence frequencies in aIL compared to water.

[f] Proportion of aIL that showed significantly different occurrence frequencies.

As to the second PP, we observed a complex pathway of residues with significantly altered hydrogen bonds and distances that originated at the surface residues L108 and eventually reached the catalytic site residue S77 via a twisting motion of residues G103 to T109 (Fig. 5D). Upon interaction with [TfO^–^], L108 moved closer towards A81, inducing a twisting motion of helix α7 as well as residues G103, G104, A105, and N106, which was reflected in significantly altered hydrogen bond frequencies (Table 3) and intramolecular distances (Fig. 5B). Due to a significantly strengthened interaction of the backbone oxygen of A105 with the backbone nitrogen of S77, the conformational change was transmitted to the catalytic site. The long-range perturbation led to a redistribution of rotamer populations adopted by the catalytic residue S77, reducing the proportion of the active conformation in favor of other, catalytically inactive conformations (Fig. S14, Text S2 in SI). As to the rigidity analysis, this reorganization led to a more rigid local structure due to strengthened interactions of residues G103-T109 with S77-A81 (Fig. 5D).

**Table 3:** Occurrence frequencies of H-bonds affected in PP2 in 0.7 M [BMIM/TfO] compared to water.

H-Bond	Water [a]	[BMIM/TfO] [b]	Difference [c]	*p*-value [d]
T101Oγ-N106Nδ	0.11	0.31	+0.20	<0.01
G104O-N106Nδ	0.19	–	−0.19	0.04
N106O-T110N	0.14	0.20	+0.06	0.09
N106O-T109N	0.27	0.35	+0.08	0.02
A105N-S76O	0.19	0.51	+0.32	0.02
S77Oγ-H76Nδ	0.11	0.17	+0.06	0.03

[a] Occurrence frequency of H-bonds in water.

[b] Occurrence frequency of H-bonds in 0.7 M [BMIM/TfO].

[c] Difference in the occurrence frequency between 0.7 M [BMIM/TfO] and water.

[d] *p*-value determined by a two-sided independent Student’s *t*-test.

To conclude, we showed that aIL induce significant changes in the intramolecular hydrogen bond network, which are related to changes in local structural stability. These changes may affect the integrity of the protein core (PP1) and the catalytic site (PP2).

### 2.6 Substitutions at positions that are part of a PP yield a gain-in-precision over random mutagenesis for improved residual activity of *Bs*LipA

We assessed if substitutions at residues identified as part of a PP yield a higher likelihood for significantly improved residual activity (Eq. (6)) than random mutagenesis (Eq. (7)). To do so, we computed the gain-in-precision (*GiP*) (Eq. (8)) for introducing substitutions there with respect to the precision due to random mutagenesis. Across the entire SSM library [26] for 0.9 M [BMIM/Br], 1.2 M [BMIM/Cl], 0.6 M [BMIM/I], and 0.7 M [BMIM/TfO], 206, 462, 263, and 292 of 3620 variants show significantly increased tolerance for the respective aIL, corresponding to a precision due to random mutagenesis of 6–13%. The *GiP* was calculated for substituting the solvent-exposed residues of PP1 to R/K to repel cation binding or PP2 to D/E to repel anion binding.

**Fig. 6:**
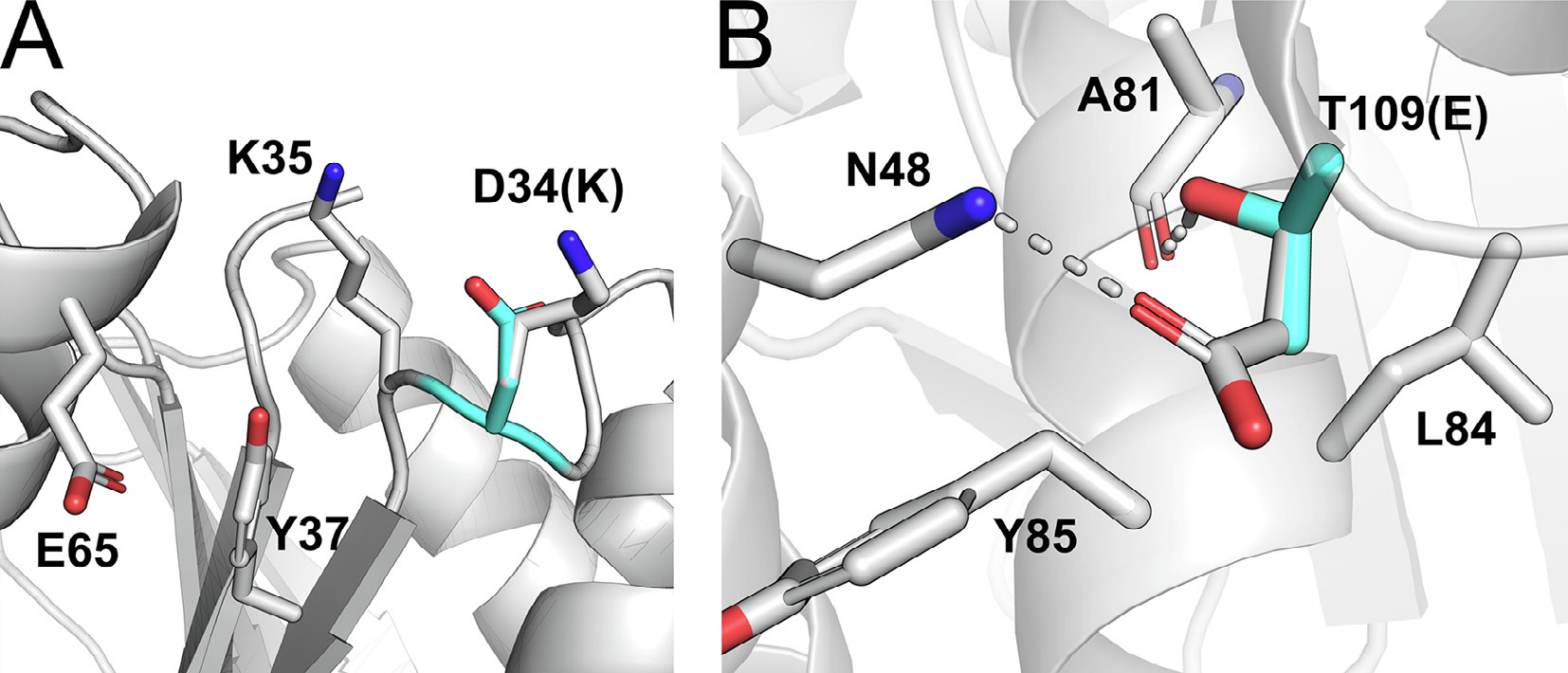
Potential resistance mechanisms of the aIL-resistant D34K and T109E variants. (A) Residues involved in intramolecular interactions with D34 (cyan sticks) or K34 (white sticks) are shown as sticks. In the D34K variant, the introduced lysine residue most likely repels [BMIM^+^] ions around the residue patch, preventing potential conformational changes introduced upon binding of [BMIM^+^] to D34 and its adjacent residues. (B) Residues involved in intramolecular interactions with T109 (cyan sticks) or E109 (white sticks) are shown as sticks. In the T109E variant, the hydrogen bond between T109Oγ and the backbone oxygen of A81 with a distance of 1.8 Å is replaced with a strong ionic interaction of E109Oε with N48Nε (distance of E109Oε to N48Nε is 2.8 Å). The introduced negative surface charge most likely also reduces the occurrence frequency of [TfO^–^] ions around the residue patch. (For interpretation of the references to color in this figure legend, the reader is referred to the web version of this article.)

In PP1, D34 is one of the residues with the highest increase in tolerance upon substitution towards all tested aIL (2.4–4.1-fold) when substituted with positively charged residues. For PP1 involving the solvent-accessible residues H3, D34, K35, Y37, and E65, a cation-induced structural reorganization was suspected. Therefore, we excluded the positively charged residue K35, which already should be beneficial for reducing cation binding, and residue H3, as it is part of a π-stacking interaction with W31. Considering only substitutions at the remaining positions resulted in an average *GiP* of 4.0 (5.4, 2.4, 4.2 and 3.8 for 0.9 M [BMIM/Br], 1.2 M [BMIM/Cl], 0.6 M [BMIM/I], 0.7 M [BMIM/TfO], respectively). A potential resistance mechanism of the D34K variant that showed substantially increased resistance to all aIL (2.9-fold to 4.1-fold) is shown in Fig. 6A. For the D34K variant, visual inspection suggests that the introduced lysine residue most likely repels [BMIM^+^] molecules around the residue patch in contrast to forming stabilizing intramolecular interactions, such as salt bridges or charge-assisted hydrogen bonds, preventing potential conformational changes that are introduced upon binding of [BMIM^+^] to D34 and its adjacent residues. The substitution was predicted by FoldX [71] to be overall neutral with respect to changes in the unfolding free energy ΔΔ*G*_unf_ compared to *Bs*LipA_wildtype_ (−0.17 kcal mol^−1^), indicating that the significant increase in aIL resistance is not induced by an increase in thermodynamic stability of the *Bs*LipA variant but instead by effects based on changes in the overall aIL-protein interactions.

In PP2, which involved the solvent-accessible residues L108 and T109, an anion-induced structural reorganization was suspected. Considering only substitutions at these positions resulted in a *GiP* of 2.9 for 0.7 M [BMIM/TfO]. Interestingly, for these substitutions, all other libraries also revealed an increased *GiP* of 4.0, 3.6, and 3.2 in 0.9 M [BMIM/Br], 1.2 M [BMIM/Cl], and 0.6 M [BMIM/I], respectively, thus potentially highlighting a general beneficial substitution site of the *Bs*LipA structure. A potential resistance mechanism of the T109E variant that showed substantially increased resistance to all aIL (1.9-fold to 2.4-fold) is shown in Fig. 6B. In the T109E variant, the hydrogen bond (1.8 Å) of T109Oγ with the backbone oxygen of A81 is replaced with a strong ionic interaction of E109Oε with N48Nε (2.8 Å). The introduced negative surface charge most likely also reduces the spatial distribution of [TfO^–^] molecules around the residue patch. Notably, the substitution was predicted by FoldX [71] to be destabilizing with respect to changes in the unfolding free energy ΔΔ*G*_unf_ compared to *Bs*LipA_wildtype_ (3.26 kcal mol^−1^), indicating that beneficial substitutions could be missed when relying on single evaluation criteria, such as ΔΔ*G*_unf_ [71].

To conclude, substitutions at residues that are part of a perturbation pathway yield pronounced *GiP* for the improved residual activity of *Bs*LipA. Therefore, positions in such pathways are valuable targets for introducing focused substitutions that counteract the proposed mechanisms of aIL-induced enzyme inactivation.

## 3 Discussion

In this study, we showed that aIL ions preferentially interact with specific *Bs*LipA surface residues and induce changes in the local protein stability through a redistribution of intramolecular interactions. Such changes percolate over neighboring residues to distant regions of the protein and may affect the integrity of the protein core and the catalytic site. Finally, by data mining an experimental complete SSM library of *Bs*LipA, we show that residues that are part of a PP yield pronounced *GiP* for the improved residual activity of *Bs*LipA.

Our results are based on unbiased all-atom MD simulations of 2 µs length per replica, yielding an aggregate simulation time of 130 μs, which, to our knowledge, are the most extensive MD simulations of a protein in aIL to date. We used state-of-the-art force fields [58], [72] and water models [59] that are well established and have been shown by us (Fig. S1) and others [73] to reproduce properties of IL and aIL well. We paid particular attention to obtain converged properties of our simulation systems relevant for this study by monitoring I) the spatial distribution of aIL ions around *Bs*LipA over time, II) the evolution of binding free energies of IL ions to *Bs*LipA residues over time, and III) the backbone RAC of *Bs*LipA (Fig. 1). We showed that ∼ 1.8 µs of simulation time was required to achieve converged results, suggesting that previous MD simulations of protein-aIL systems with simulation times of ∼ 10–100 ns [36], [39], [55] were highly likely too short to yield statistically converged results for assessing protein-ion interactions. Alternative approaches to understand the influence of co-solvents on proteins are provided by the Kirkwood-Buff theory and the preferential binding model [74], although the indistinguishable ion approach used there prohibits a more detailed analysis of ion distributions around the protein [75]. As to our absolute binding free energy computations, we ensured that our results are not biased by kinetic trapping by probing for the number of binding and unbinding events. The Δ*G*^0^ results obtained agree with literature values obtained for a similar setting [64]. So far, only a few publications have investigated the energetics of IL ion binding to proteins in detail [75]. For alkylimidazolium-based IL, simulation results revealed a complex, enthalpically driven binding mechanism [76]. Finally, the CNA approach to probe structural stability has been applied before retro- [77], [78], [79], [80], [54] and prospectively [67], [81] in the context of improving protein thermostability on pairs [79], [80] and series [77], [78], [81] of proteins. It was also benchmarked against the complete SSM library of *Bs*LipA [53] for systematically scrutinizing the impact of substitution sites on thermostability and detergent tolerance [54]. The breadth of these CNA applications is rooted in the rigorous analysis of structural rigidity [82] and the use of structural ensembles to improve robustness [83].

Several effects of enzyme inactivation due to aIL have been suggested [36], [41], [48]. Here, we focused on indirect effects because their molecular basis remains mostly unknown. Initially, in contrast to previous studies [29], [36], [39], [45], [84], we show that aIL ions preferentially interact with a few specific *Bs*LipA surface residues, as demonstrated by computed absolute binding free energies (Δ*G*^0^), which were found to be negative at 1 M standard state only for ∼ 2–8% of all surface residues. Mostly large and weakly hydrated organic ions interacted strongly with *Bs*LipA. These results suggest that qualitative inspection of spatial distributions of solvent molecules (including aIL ions) as done previously [36], [39] may be too coarse to identify the most targeted surface regions. Furthermore, in line with previous studies [39], [41], [48], we identified preferred types of residues interacting with IL ions, including A20, K23, W42, Y49, R107, and L108. Compared to previous computational studies investigating the same aIL/enzyme system, we observed a solvent-specific cooperative binding effect for [BMIM/TfO] that induced a unique interaction site for [BMIM^+^] molecules at the catalytic site cleft and may affect enzyme activity in this aIL similarly to the crowding of [BMIM^+^] molecules observed for the I12F variant [36]. Notably, however, our results indicate that general physicochemical properties, e.g., the charge or electronic state, may be too vague to classify preferred types strictly, as D and E behave markedly different against [BMIM^+^], as does W compared to F and Y (Fig. 2C). Although our protein-wide description of aIL-protein binding free energies, to our knowledge, is currently the most comprehensive and quantitative analysis of that type, we note that it depends on the definition of bound aIL ion states. Here, we chose a definition widely applied before [39], [85], [86].

As before [26], [47], [48], we did not find pronounced changes in the global structure or dynamics upon incubation in aIL that could explain the reduced enzyme activity (Fig. 3). It has also been suggested that aqueous ILs may induce enzyme aggregation, e.g., as a result of local fluctuations in enzyme structure that result in increased exposure of hydrophobic protein residues [27], [35]. However, we did not observe significant increases in the hydrophobic surface area of *Bs*LipA between water and aIL or salt solutions, indicating that enzyme aggregation is likely not the predominant cause for activity reduction of *Bs*LipA in aIL. Furthermore, and unexpected at first, residues most preferentially interacting with IL ions, such as W42, were not identified as sites that yield a *GiP* over random mutagenesis for improved residual activity in the experimental SSM library (see Tables S7–S10 in ref. [26]). However, the binding mechanisms of IL ions have been described as more complex than found for most other co-solutes [87], [88]. This results in ion-protein and ion-ion correlation effects [89] such that effects on protein stability can also be expected if several residues are involved that interact less preferentially with IL ions. Note in this context that Δ*G*^0^ of ∼ 0.5 or ∼ 0.8 kcal mol^−1^ as found for [TfO^−^] binding to K/R or [BMIM^+^] binding to aromatic residues still relates to bound proportions of ∼ 0.43 and ∼ 0.26 at 300 K and 1 M standard state.

Hence, we decided to probe the complex influence of IL ion binding to *Bs*LipA by postprocessing conformations from MD simulations with CNA. We observed local, but not global, changes in structural stability, concordant with the analysis of global structure and dynamics. In most cases, an increase in structural stability was observed, which may have direct functional implications for *Bs*LipA activity, as enzyme flexibility is often associated with enzyme activity [90], [91], [92], [93], [94]. A notable example is the loop containing the catalytic site residue D133 [46], which becomes rigidified in all aIL (Fig. 4C–F). Interestingly, destabilizing effects occur less often, but were observed for, e.g., the loop residues H10-A20, which became more flexible in 0.6 M [BMIM/I]. Regions with altered protein stability on the surface markedly overlapped with binding sites of aIL ions with *Bs*LipA (Fig. 4C–F), suggesting that the effects originate from interactions with IL ions. System-specific changes in structural stability in 0.6 M [BMIM/I] and 0.7 M [BMIM/TfO] further suggest that distinct anion properties, e.g., size, shape, or polarity, can lead to different effects as summarized for other proteins systems in ref. [75].

Although CNA provides detailed information on the impact of IL ion interactions on *Bs*LipA structural stability, it does not reveal the underlying changes in molecular interactions within the protein. Therefore, we analyzed the MD trajectories towards changes in molecular interactions, focusing on hydrogen bonds and salt bridges, as they should be enthalpically driven [95], similar to the binding mechanism of alkylimidazolium ions [76]. The results showed that intramolecular interactions underwent reorganizations in multiple regions. In some cases, these changes percolated over neighbor residues to distant areas of the origin, thereby forming PP. Many residues involved in PP1 and PP2 agreed with regions with altered structural stability observed in CNA (Fig. 5C/D), indicating that rigidity-analyses of MD ensembles could be used to point to the location of PPs.

The PP provide a molecular explanation for the long-range effects of aIL on *Bs*LipA. These indirect effects were observed in other studies [36], [39] and can explain how aIL effects on the surface can be transmitted to the catalytic site, where already small deviations from the optimal geometry were shown to have substantial effects on *Bs*LipA activity [96], [97]. Two *Bs*LipA variants K23E and I151W were investigated in ref. [36]; they were suggested to decrease *Bs*LipA activity by structural reorganizations of the local protein structure upon interacting with aIL, which in the latter case also affected the catalytic site. Interestingly, the conformation of S77 in the perturbed I151W variant is not in the native (active) conformation [36], corroborating our results of an aIL-induced redistribution of conformations adopted by S77. Data mining the experimental complete SSM library [26] strongly supported the impact of interaction reorganizations by aIL on *Bs*LipA activity in that substitutions at residues that are part of a PP yield pronounced *GiP* for an improved residual activity of *Bs*LipA (see Section 2.6). Therefore, such pathways are valuable targets for introducing focused substitutions that counteract the proposed mechanisms of aIL-induced inactivation.

These results imply that enzyme tolerance against alkylimidazolium-based aIL can be efficiently increased by identifying and subsequently modifying aIL-induced PP. For the identification, structural ensembles of the target enzyme in aIL generated via long MD simulations are screened towards aIL-induced PP; this screening can be supported by a prior analysis of changes in local structural stability. The number of substitution sites in PP can be further reduced by considering that predominantly solvent-exposed residue side chains interact with IL ions and that substitutions at buried positions are more likely to yield inactive variants [26], [54], [98]. Finally, by determining specific IL ion-residue interactions guided by Δ*G*^0^ computations, beneficial substitutions can be suggested, e.g., to R/K for cation-based PP and to D/E for anion-based PP.

PP are a so far underexplored mechanism of how aIL-protein interactions influence *Bs*LipA activity. Exploiting them appears superior to applying rules that suggest site-unspecific charge modifications or try to improve protein hydration in a general manner for increasing enzyme tolerance towards aIL [39], [45], [99] as they pinpoint affected surface residues and provide suggestions for specific beneficial substitutions. Notably, we observed additional effects that may affect *Bs*LipA activity and could be targeted to improve aIL resistance of *Bs*LipA, going beyond effects shown to affect *Bs*LipA activity in a study investigating resistant and non-resistant *Bs*LipA variants [36]. Effects observed by us include the increased presence of [BMIM^+^] molecules around the catalytic site due to interactions with a residing [TfO^−^] and the distorted integrity of the catalytic triad, which presumably directly originates from the PP2 in [BMIM/TfO]. Further, we are aware that our analysis is limited to one enzyme here. Still, many variants with the highest increase in aIL tolerance identified in the experimental complete SSM library [26] corroborate the importance to address specific sites: They introduce site-specific hydrophobic effects and additional salt bridges/hydrogen bonds, or increase the polarity of the catalytic site cleft, instead of non-specifically repelling aIL [36]. Furthermore, an NMR-guided surface charge engineering approach [48] based on structural perturbations also showed the potential benefits of site-specific surface charge modification to improve *Bs*LipA tolerance towards [BMIM/Cl]. While this supports our approach that interactions of aIL inducing structural perturbations and long-range effects in enzyme structures can be exploited in rational mutagenesis approaches, our approach does not require demanding NMR experiments.

In summary, we show that binding of IL ions to specific surface residues redistributes the local protein stability and that these changes percolate to distant areas of the enzyme, representing a molecular mechanism for long-range effects of aIL. We postulate that these residues represent key positions in the *Bs*LipA structure that can be exploited to guide the rational design of novel enzyme variants with improved aIL resistance and present an approach to determine such positions. The approach takes about one week of simulation time for enzyme systems with average size on current general-purpose GPUs, can lead to a high *GiP* compared to random mutagenesis, and, thus, is a valuable way for the design of focused variant libraries.

## 4 Materials and methods

### 4.1 System preparation for molecular dynamics simulations

To investigate the interactions of *Bs*LipA with aIL and salt solutions, we performed all-atom, unbiased molecular dynamics (MD) simulations of *Bs*LipA in respective solvents used for evaluating the SSM library [26]. The used ion concentrations and the residual activity of *Bs*LipA in the respective solvents are listed in Table 4.

**Table 4:** Experimental conditions for the investigated solvents.

Solvent	[BMIM/Br]	[BMIM/Cl]	[BMIM/I]	[BMIM/TfO]
Concentration [a]	0.9	1.2	0.6	0.7
Relative residual activity [b]	0.39	0.35	0.37	0.30
Standard deviation [c]	14.5	11.3	12.3	13.0

[a] In M.

[b] Residual activities are given relative to *Bs*LipA in buffer.

[c] Standard deviation of the residual activity in the respective aIL screening system after background subtraction, in % (*n* = 96).

**Table 5:** System setup of investigated solvents.

	MD00	MD01	MD02	MD03	MD04	MD05	MD06	MD07	MD08	MD09	MD10	MD11	MD12
Cation	–	BMIM	BMIM	BMIM	BMIM	BMIM	BMIM	Na	Na	Na	Na	K	K
Anion	Cl	Br	Cl	I	TfO	Cl	I	Cl	Cl	I	I	Cl	Cl
Conc. [a]	–	0.9	1.2	0.6	0.7	0.6	1.2	0.6	1.2	0.6	1.2	0.6	1.2
*n*Cation	–	292	390	194	104	180	361	180	361	180	361	180	361
*n*Anion	4	296	394	198	308	184	365	184	365	184	365	184	365
*n*Water	15,537	16,392	15,856	16,929	16,351	15,753	14,600	16,564	16,584	16,510	16,226	16,297	15,799
V_Box_[b]	535,148	651,694	657,895	666,181	617,658	605,774	624,624	605,968	610,635	623,629	623,185	593,654	616,163

[a] Concentration, in M.

[b] In Å^3^.

**Table 6:** Parameters of validation systems.

	S00	S01	S02	S03	S04	S05	S06	S07	S08	S09	S10	S11	S12	S13
Cation	–	BMIM	BMIM	BMIM	BMIM	BMIM	BMIM	BMIM	BMIM	BMIM	BMIM	BMIM	BMIM	BMIM
Anion	–	Br	Br	Br	Br	Br	Cl	Cl	Cl	Cl	Cl	Cl	Cl	Br
Conc. [a]	–	0.0315	0.0721	0.1118	0.1469	0.1849	0.0321	0.0730	0.1030	0.1403	0.1737	0.2127	0.005	1.309
*n*Cation	0	8	22	34	46	58	10	22	32	44	54	66	1	414
*n*Anion	0	8	22	34	46	58	10	22	32	44	54	66	1	414
*n*Water	17,626	17,580	17,498	17,428	17,358	17,288	17,570	17,500	17,444	17,374	17,316	17,248	17,616	15,201
V_Box_[b]	512,000	512,000	512,000	512,000	512,000	512,000	512,000	512,000	512,000	512,000	512,000	512,000	512,000	512,000

[a] Concentration, in M.

[b] In Å^3^.

To test for ion- and concentration-specific effects, the range of solvents was extended to salt solutions containing [Na^+^] or [K^+^] cations and [Cl^−^] or [I^−^] anions in the lowest and highest concentrations (0.6 M and 1.2 M). A solution of 0.6 M [Na/Cl] mimics natural seawater of ∼ 0.67 M [100].

The coordinates for the starting structure of *Bs*LipA were taken from the crystal structure with PDB-ID 1I6W [46], chain A. Protonation states for all titratable amino acids were assigned according to pH 7.4 using the program Epik [84], [101], which is part of the Protein Preparation Wizard [102] included in Schrödinger’s Maestro program suite [103]. All hydrogen atoms of the crystal structure were removed using the REDUCE program [104] and reassigned with the program LEaP [105] according to the Amber ff14SB library [72], which is included in the AMBER18 program package [106]. We added an ACE cap group at the N-terminal amino acid to avoid an artificially charged terminus. As to the IL ions, the initial 3D structures (Fig. S15) were prepared by using LEaP [105] from AMBER18 [106]. The structures were subjected to quantum mechanical (QM) geometry optimization using Gaussian 16 [107] at the HF/6-31G* level of theory [108]. The resulting parameters, e.g., the atomic partial charges, were compared with parameters from dedicated IL force fields, such as the OPLS force field [109], [110], and found to be in good agreement. The QM-optimized structures were used as starting structures for MD simulations.

Packmol [111] was initially used to place one *Bs*LipA in the center of a cubic simulation box and then to randomly add the needed amount of the respective cations and anions to reflect the desired concentration, with four additional anions to obtain electroneutrality. Since periodic boundary conditions were used, a minimal distance of 15 Å from the protein to the box sides was used to prevent self-interaction of the protein across the box borders. For simulations of *Bs*LipA in pure water, four [Cl^−^] ions were added as counterions. The systems were then solvated using the OPC water model [59], also by using Packmol [111]. The final amount of water molecules and ions as well as the box volume for each solvent is shown in Table 5.

For *Bs*LipA, atomic partial charges and force field parameters were taken from the Amber ff14SB force field [72]. Atomic partial charges for IL were derived according to the RESP procedure [112]. The force field parameters for IL were taken from the general amber force field (GAFF) [58].

### 4.2 Molecular dynamics simulations

The simulations were performed following ref. [69]. The systems were first subjected to three rounds of energy minimization to eliminate steric clashes. First, harmonic restraints with a force constant of 5 kcal mol^−1^ Å^−2^ were applied to all protein atoms for 2500 cycles (500 cycles steepest descent (SD) followed by 2000 cycles conjugate gradient (CG) minimization). Second, the harmonic restraints were reduced to a force constant of 1 kcal mol^−1^ Å^−2^ for 10,000 cycles (2000 cycles SD and 8000 cycles CG minimization). Third, 1000 cycles SD and 4000 cycles CG minimization without any restraints were performed.

In the subsequent thermalization, the system was first heated from 0 K to 100 K over 50 ps in a canonical (NVT) MD simulation. Harmonic restraints of 1 kcal mol^−1^ Å^−2^ were applied on protein atoms, and a time step of 2 fs was used. The temperature was then raised from 100 K to 300 K over 50 ps of isobaric-isothermal (NPT) MD simulations. Subsequently, the density was adapted to 1 g cm^−3^ over 200 ps of NPT–MD simulations. Finally, the harmonic restraints were reduced to 0 kcal mol^−1^ Å^−2^ over the course of six NVT–MD simulations with a length of 50 ps each. In all MD simulations, the particle mesh Ewald (PME) method [113] was used to treat long-range electrostatic interactions. The distance cutoff for short-range non–bonded interactions was set to 9 Å. Langevin dynamics were used with a time constant (*τ*) of 0.5 ps for heat bath-coupling to keep the system temperature at the target temperature of 300.0 K during the simulations. The SHAKE [114] algorithm was applied to all bonds involving hydrogens. To set up five independent MD production simulations, the target temperature during thermalization varied from 299.8 K to 300.2 K in 0.1 K intervals.

The production simulations were performed in the NPT ensemble at 300.0 K for 2.0 µs with a time step of 4 fs using the hydrogen mass repartitioning (HMR) method [115]. Coordinates were saved every 20 ps. All computations were performed using the GPU-accelerated version of pmemd [116] from the AMBER18 program suite [106].

### 4.3 Trajectory analysis

Only the last 1.8 μs of MD simulations, corresponding to 9000 frames, were used for analysis. The analyses were performed with *cpptraj* [117] from the AmberTools18 package [106]. The following measures were evaluated: I) the root-mean-square deviation (RMSD) as a measure of structural similarity and II) the root-mean-square fluctuation (RMSF) as a measure of mobility (in both cases, fitting was performed to the backbone atoms of the crystal structure); III) the solvent-accessible surface area (SASA) and IV) the radius of gyration (ROG) as measures of protein (un-)folding; IV) the number of water molecules in the first hydration shell of the protein (<3.4 Å) as a measure for protein hydration; V) the radial distribution function, VI) solvent density grids, and VII) distances to describe interactions of solvent molecules with *Bs*LipA; VIII) the analysis of hydrogen bonds to describe the intramolecular polar interaction network; IX) dihedral angles of the catalytic site residue S77 to describe the conformational state of the catalytic site; X) intramolecular distances of functional side chain groups (see Section 4.6) and backbone atoms of *Bs*LipA residues to each other. Using the results from the interaction analysis (VII), dissociation constants and absolute binding free energies were computed for each individual protein residue for both solvent cation and anion, respectively (see Section 4.7). Results over five independent trajectories of the same system are shown as means ± standard errors of the mean (SEM) and were analyzed with the R software [118] using the two-sided independent Student’s *t*–test. Results with *p*–values ≤ 0.05 were considered significant.

### 4.4 Quality assessment of the chosen force field/water model/partial charge combination

For the validation of the chosen system parameters and force field/water model/partial charge combination, we set up systems analogous to the procedure described in Section 4.1 following experimental conditions from ref. [119] or MD simulation studies in ref. [120], [121], respectively. Box side lengths of 80 Å for all systems were used to define the system dimensions. We then added IL ions and solvated the system with water molecules, using the respective TIP3P or OPC water model. Temperatures of 298.15 K or 300.00 K were used, following the specifications in the respective references. Minimization and equilibration schemes with adapted temperatures were performed as described in Section 4.2. Production runs were performed for 200 ns with five replicas each, with only the last 150 ns used for analyzing the system density (systems S00–S11, Table 6). For analyzing the self-diffusion coefficient, production runs were performed for ten replicas each, with simulation times of 100 ns or 200 ns for [BMIM/Br] or [BMIM/Cl], respectively, to achieve more robust statistics (systems S12–S13, Table 6).

### 4.5 Calculation of the system-specific correction factor

To determine the system-specific correction factor *F*_i_, which describes the relative amount of water molecules in the system compared to a simulation in pure water, two systems were set up for each solvent with identical dimensions and parameters as described in Section 4.1, one as aIL and one in pure water. The correction factor for each system is given in Eq. (1)

(1) $$F_{i}=\frac{{nWAT}_{aIL}}{{nWAT}_{WAT}}$$

where *n*WAT_aIL_ is the number of water molecules in the aIL system, and *n*WAT_WAT_ is the number of water molecules in the system with pure water.

### 4.6 Definition of bound/unbound states for ion-protein interactions

For the analysis of interaction frequency of *Bs*LipA residues, we computed the distances between the center of mass of every ion to the center of mass of functional groups of every *Bs*LipA residue (Table 7) over the course of the trajectory. The cutoffs for the subsequent analyses were derived from the average radial distribution function (RDF) for solvent ions around every *Bs*LipA residue using the center of mass (COM) as defined in Table 7 and the COM of solvent ions. For [BMIM^+^] and [TfO^−^], the N2 or C1 atoms were used to represent the COM, respectively. The trajectories of the aIL with experimental conditions were used to calculate the anion RDFs. For the calculation of cation RDFs, the systems of [BMIM/Cl], [Na/Cl], and [K/Cl] at a concentration of 0.6 M were used. All RDFs were normalized according to the average system density of the input frames. The radial distribution functions of ions at the protein surface (Fig. S6) showed contact distances between 1.8 and 5.4 Å. For [Na^+^], [K^+^], and [BMIM^+^], distances ≤ 3.6, 4.0, and 4.9 Å indicate bound states, and larger distances unbound states; for [Cl^−^], [Br^−^], [I^−^], and [TfO^−^], the cutoff values are 4.8, 5.0, 5.2, and 5.4 Å, respectively.

**Table 7:** Atoms representing functional groups of *Bs*LipA residues according to Amber ff14SB atom names [72].

Res	Atoms	Res	Atoms	Res	Atoms	Res	Atoms
A	CB	**D**	OD1, OD2	**I**	CB, CG1, CG2, CD1	**F**	CG, CD1, CD2, CE1, CE2, CZ
G	CA	**E**	OG1, OG2	**T**	CB, OG1, CG2	**H**	CG, ND1, CD2, CE1, NE2
K	NZ	**N**	ND2, OD1	**P**	CB, CG, CD	**W**	CG, CD1, CD2, NE1, CE2, CE3, CZ2, CZ3, CH
ACE	C	**Q**	NE1, OE1	**V**	CB, CG1, CG2	**Y**	CG, CD1, CD2, CE1, CE2, CZ, OH
S	OG	**R**	NE, NH1, NH2	**M**	CE, SD, CG, CB	**L**	CB, SG, CD1, CD2

### 4.7 Calculation of dissociation constants and binding free energies of *Bs*LipA residues with ionic liquid ions

To investigate the interactions of *Bs*LipA surface residues with IL ions, we computed the interaction frequencies for all residues with each solvent ion. The frequencies are based on distances between the functional groups of residues and the COM of ions as defined in Section 4.6. If a distance is smaller than or equal to an ion-specific cutoff, the state was classified as *bound*, and *unbound* otherwise. The ion-specific cutoffs were obtained from analyses of the RDF (Fig. S6). The number of bound states was related to the number of frames to yield the *bound fraction*. Subsequently, dissociation constants for each residue were calculated according to Eq. (2).

(2) $$K_{D}=\frac{\left(1-\alpha )\times [I\right]}{\alpha },with\left\{\begin{array}{c} \alpha =bound\;fraction\\ \left[I\right]=ion\;concentration \end{array}\right)$$

Binding free energies (Δ*G*^0^) with *C*^0^ as the standard concentration of 1 M were then computed according to Eq. (3).

(3) $$\Delta G^{0}=RT∙\ln{(K}_{D}/C^{0}),with\left\{\begin{array}{c} R=1.9872\;cal\;K^{-1}\;{mol}^{-1}\\ T=300.0\;K \end{array}\right)$$

### 4.8 Calculation of intramolecular hydrogen bond frequencies

To investigate changes in the intramolecular hydrogen bond network of *Bs*LipA, we computed the average occurrence frequency of each hydrogen bond over the course of the trajectory. As donor heavy atoms, we considered all O/N atoms bound to hydrogen. As acceptor atoms, all O and N atoms were considered. A hydrogen bond was assigned if the distance between the donor heavy atom and the acceptor atom was ≤ 3.0 Å and the angle between donor heavy atom, donor hydrogen, and acceptor atom was ≥ 135°. Only hydrogen bonds with average occurrence frequencies ≥ 5% in at least one solvent were considered, which resulted in 325 individual hydrogen bonds over all 13 solvents. All hydrogen bonds of the same donor and acceptor atom types were summarized to consider side-chain rotations around single bonds. E.g., this reduces the number of potential hydrogen bonds for an Arg-Glu interaction from eight (for hydrogen bonds between either Oδ atom to each one of the Hη atoms of either Nη atom) to one. For the analysis of the hydrogen bonds involved in the catalytic site network, looser distance and angle cutoffs for hydrogen bonds of 3.5 Å and 120° were used.

### 4.9 Constraint network analysis

CNA was performed as described in ref. [54]: The Constraint Network Analysis (CNA) aims at linking structural rigidity and flexibility to the biomolecule’s structure, (thermo)stability, and function [66], [70], [122]. The CNA software acts as front- and back-end to the graph theory-based rigidity analysis software Floppy Inclusions and Rigid Substructure Topography (FIRST) [82]. In CNA, proteins are modeled as constraint networks in a body-and-bar representation, which has been described in detail by Hesphenheide *et al.* [123] Based on the modeled constraint network of the protein structure, a pebble game algorithm decomposes the network into flexible and rigid subparts [124], [125]. To monitor the decay of network rigidity and to identify the rigidity percolation threshold, CNA performs thermal unfolding simulations by consecutively removing noncovalent constraints (hydrogen bonds, including salt bridges) from a network in increasing order of their strength [126]. For this, a hydrogen bond energy *E*_HB_ is computed by a modified version of the potential by Mayo *et al.* [126]. During the thermal unfolding simulations, phase transitions can be identified where the network switches from overall rigid to flexible states. For a given network state σ = f(*T*), hydrogen bonds with an energy *E*_HB_ > *E*_cut_(σ) are removed from the network at temperature *T*. In this study, the thermal unfolding simulation was carried out by decreasing *E*_cut_ from −0.1 to −6.0 kcal mol^−1^ with a step size of 0.1 kcal mol^−1^. *E*_cut_ can be converted to a temperature *T* using the linear equation introduced by Radestock *et al.* (Eq. (9)) [79], [80]. The range of *E*_cut_ is equivalent to increasing the temperature from 302 to 420 K with a step size of 2 K. Because hydrophobic interactions remain constant or become even stronger as the temperature increases [127], [128], the number of hydrophobic tethers was kept unchanged during the thermal unfolding simulation, as done previously [54], [67], [77].

(9) $$T=\frac{-20K}{kcal\cdot {mol}^{-1}}E_{cut}+300K$$

The CNA software is available under academic licenses from http://cpclab.uni-duesseldorf.de/index.php/Software. The CNA web server is accessible at http://cpclab.uni-duesseldorf.de/cna/.

The residue-wise extent of the structural stabilization and destabilization with regard to water, Δ*E_i_*_,CNA,_ was calculated as the sum over all *n* short-range rigid contacts in which residue *i* is involved in as described in ref. [68] (Eq. (4)):

(4) $${\Delta E}_{i,CNA}=\frac{1}{2}\sum_{j\neq i}^{n}{\mathrm{rc}}_{\mathrm{ij}}$$

The overall descriptor of the protein stability *E*_CNA_ was calculated as the sum of all observed interactions in *rc_i,j_* (Eq. (5)):

(5) $$E_{CNA}=\sum_{i}^{n}\sum_{j>i}^{n}{\mathrm{rc}}_{\mathrm{ij}}$$

### 4.10 Calculation of residual activity of a *Bs*LipA variant

The residual activity (RA) of variants was calculated following (Eq. (6)):

(6) $$Residual\;activity=\frac{slope\left(BsLipA\;variant\;or\;WT-EV\right)\;in\;aIL}{slope\left(BsLipA\;variant\;or\;WT-EV\right)\;in\;buffer}$$

where EV indicates the noise measured for the empty vector. The tolerance was considered significantly improved, when RA_aIL_ ≥ RA_buffer_ + 3 σ_aIL_, with RA_aIL_ and RA_buffer_ being the residual activity of the variant in aIL and buffer, respectively and σ_aIL_ being the true standard deviation of the assay [26]. Here, 3 σ_aIL_ was chosen because it corresponds to a *p*-value below 0.01. For a detailed description of how cultivation, purification, and activity assays were performed, please see ref. [26].

### 4.11 Definition of descriptors to evaluate mutagenesis efficiency

To evaluate whether the identified substitution sites improve the precision of substitution prediction to improve aIL tolerance of *Bs*LipA variants, we first calculated the precision *P* (Eq. (7)) and then the gain-in-precision *GiP* (Eq. (8)) when considering only a subset of the complete library (in total, 3620 variants at 181 positions) derived by filtering the variants by a specific property (e.g., location in a PP) with respect to random mutagenesis over the whole library for each solvent.

The precision *P* was defined as the fraction of the number of variants with increased tolerance towards aIL divided by the number of considered variants (Eq. (7)).

(7) $$P=\frac{\mathrm{Variants}\;with\;incr.\;aIL\;tolerance}{\mathrm{Considered}\;variants}$$

The *GiP* was calculated by dividing the precision over a subset of residues considered by the precision over the whole library, which is equivalent to the result due to random mutagenesis (Eq. (8)).

(8) $$\mathrm{GiP}=\frac{\mathrm{Precision}\;over\;a\;subset}{\mathrm{Precision}\;over\;the\;whole\;dataset}$$

## Declaration of Competing Interest

The authors declare that they have no known competing financial interests or personal relationships that could have appeared to influence the work reported in this paper.
