# Supplementary material for: Aqueous ionic liquids redistribute local enzyme stability via long-range perturbation pathways
Source: Comput Struct Biotechnol J. 2021 Jul 8;19:4248–64. doi: 10.1016/j.csbj.2021.07.001 (PMC8355836; doi:10.1016/j.csbj.2021.07.001)
Supplement: Supplementary data 1 [file mmc1.pdf]

# aSupplementary Information

# Aqueous ionic liquids redistribute local enzyme stability via long-range perturbation pathways

Till El Harrar[1,2], Benedikt Frieg[2], Mehdi D. Davari[1,§], Karl-Erich Jaeger[3,4], Ulrich Schwaneberg[1,6], Holger Gohlke[*,2,5]

[1] Institute of Biotechnology, RWTH Aachen University, 52074 Aachen

[2] John-von-Neumann-Institute for Computing (NIC), Jülich Supercomputing Centre (JSC), Institute of Biological Information Processing (IBI-7: Structural Biochemistry), and Institute of Bio- and Geosciences (IBG-4: Bioinformatics), Forschungszentrum Jülich GmbH, 52428 Jülich

[3] Institute of Molecular Enzyme Technology, Heinrich Heine University Düsseldorf, 52428 Jülich

[4] Institute of Bio- and Geosciences IBG-1: Biotechnology, Forschungszentrum Jülich GmbH, 52428 Jülich

[5] Institute for Pharmaceutical and Medicinal Chemistry, Heinrich Heine University Düsseldorf, 40225 Düsseldorf

[6] DWI – Leibniz Institute for Interactive Materials e.V., 52074 Aachen

[§] Current address: Department of Bioorganic Chemistry, Leibniz Institute of Plant Biochemistry, 06120 Halle

Running title: Perturbation pathways of noncovalent interactions in aIL

Keywords: protein stability, molecular dynamics simulations, allostery, biocatalysis, ionic liquids, protein engineering

*Corresponding author:

Prof. Dr. Holger Gohlke

John-von-Neumann-Institute for Computing (NIC), Jülich Supercomputing Centre (JSC), Institute of Biological Information Processing (IBI-7: Structural Biochemistry), and Institute of Bio- and Geosciences (IBG-4: Bioinformatics)

Forschungszentrum Jülich GmbH

Wilhelm-Johnen-Str.

52428 Jülich

Email: h.gohlke@fz-juelich.de

# Table of Contents


1 Supplementary Tables ........ 2

1.1 Binding free energies[a] for all cation interactions with *Bs*LipA residues ........ 2

1.2 Binding free energies[a] for all anion interactions with *Bs*LipA residues ........ 7

2 Supplementary Figures ........ 12

2.1 Influence of water model and ion parametrization ........ 12

2.2 Time evolution of the spatial distribution of ions ........ 13

2.3 Dependency of the amount of surface waters on the amount of system waters ........ 17

2.4 Complete overview of spatial distribution for all solvent molecules ........ 18

2.5 [TfO-] induced catalytic site crowding of [BMIM+] molecules ........ 19

2.6 Radial distribution functions ........ 20

2.7 Analysis of binding and unbinding events ........ 21

2.8 Time evolution of selected binding free energies ........ 22

2.9 RMSF of *Bs*LipA backbone atoms for selected solvents ........ 23

2.10 Hydrophobic surface area ........ 24

2.11 Global protein structure descriptors ........ 25

2.12 CNA difference neighbor stability maps ........ 26

2.13 Dependency of perturbed interactions on the concentration. ........ 27

2.14 Structural reorganization of the catalytic site S77 ........ 28

2.15 Structure of the IL ions ........ 29

2.16 Conformational space analysis of S77 for all solvents ........ 30

2.17 Probabilities of the catalytic site conformations for all solvents ........ 31

2.18 Time evolution of hydrogen bond frequencies in the active site ........ 32

3 Supplementary Results ........ 33

3.1 Text S1: Dynamic properties of aIL can be reasonably well reproduced using current force field and water models ........ 33

3.2 Text S2: Incubation in aIL potentially inactivates the enzyme by inducing a shift from the active to the inactive state of the catalytic site ........ 35

3.3 Text S3: Investigation of a unique binding site in *Bs*LipA reveals an ion-specific cooperative binding effects that may impact enzyme activity through enhanced prevention of substrate access ........ 36

4 Supplementary References ........ 37

# 1 Supplementary Tables

## 1.1 Binding free energies[a] for all cation interactions with *Bs*LipA residues

**Table S1: Binding free energies[a] for cation interactions with *Bs*LipA residues**

| *ID* | *AA* | *SA[b]* | *MD01* | *MD02* | *MD03* | *MD04* | *MD05* | *MD06* | *MD07* | *MD08* | *MD09* | *MD10* | *MD11* | *MD12* |
|---|---|---|---|---|---|---|---|---|---|---|---|---|---|---|
| *Cation* | - | - | [BMIM$^+$] | [BMIM$^+$] | [BMIM$^+$] | [BMIM$^+$] | [BMIM$^+$] | [BMIM$^+$] | [Na$^+$] | [Na$^+$] | [Na$^+$] | [Na$^+$] | [K$^+$] | [K$^+$] |
| *Anion* | - | - | [Br$^-$] | [Cl$^-$] | [I$^-$] | [TfO$^-$] | [Cl$^-$] | [I$^-$] | [Cl$^-$] | [Cl$^-$] | [I$^-$] | [I$^-$] | [Cl$^-$] | [Cl$^-$] |
| *C [c]* | - | - | 0.9 | 1.2 | 0.6 | 0.7 | 0.6 | 1.2 | 0.6 | 1.2 | 0.6 | 1.2 | 0.6 | 1.2 |
| *1* | **A** | exp. | n.d.[d] | n.d. | n.d. | n.d. | n.d. | n.d. | n.d. | n.d. | n.d. | n.d. | n.d. | n.d. |
| *2* | **E** | exp. | n.d. | n.d. | n.d. | n.d. | n.d. | n.d. | n.d. | n.d. | n.d. | n.d. | n.d. | n.d. |
| *3* | **H** | exp. | 1.01 | 1.17 | 0.94 | 0.87 | 0.81 | 1.24 | 2.43 | 2.85 | 2.43 | 2.64 | 2.35 | 2.48 |
| *4* | **N** | exp. | 2.68 | 2.51 | 2.43 | 2.44 | 2.43 | 2.6 | 1.12 | 0.71 | 1.1 | 0.41 | 1.39 | 1.55 |
| *5* | **P** | bur. | -[e] | - | - | - | - | - | - | - | - | - | - | - |
| *6* | **V** | bur. | - | - | - | - | - | - | - | - | - | - | - | - |
| *7* | **V** | bur. | - | - | - | - | - | - | - | - | - | - | - | - |
| *8* | **M** | bur. | - | - | - | - | - | - | - | - | - | - | - | - |
| *9* | **V** | bur. | - | - | - | - | - | - | - | - | - | - | - | - |
| *10* | **H** | bur. | - | - | - | - | - | - | - | 2.71 | 2.43 | 2.48 | 2.43 | 2.68 |
| *11* | **G** | exp. | - | - | - | - | - | - | - | - | - | - | - | - |
| *12* | **I** | exp. | 2.02 | 2.05 | 1.97 | 1.62 | 2.02 | 2.16 | - | - | - | - | - | - |
| *13* | **G** | exp. | 0.99 | 0.99 | 1.19 | 0.83 | 1.08 | 1.02 | - | - | - | - | 2.43 | 2.85 |
| *14* | **G** | exp. | 1.7 | 1.74 | 1.85 | 1.48 | 1.87 | 1.66 | - | - | - | - | - | - |
| *15* | **A** | exp. | 1.9 | 1.89 | 1.97 | 1.86 | 1.97 | 2.01 | - | - | - | - | 2.43 | 2.43 |
| *16* | **S** | exp. | 2.21 | 2.03 | 1.86 | 1.77 | 2.16 | 1.88 | 2.35 | 2.38 | 2.35 | 2.2 | 2.35 | 2.33 |
| *17* | **F** | exp. | 0.42 | 0.38 | 0.44 | 0.33 | 0.49 | 0.4 | - | - | - | - | 2.43 | 2.38 |
| *18* | **N** | exp. | 1.54 | 1.62 | 1.69 | 1.41 | 1.53 | 1.57 | - | 2.85 | - | 2.85 | 2.43 | 2.85 |
| *19* | **F** | bur. | - | - | - | - | - | - | - | - | - | - | - | - |
| *20* | **A** | exp. | 2.16 | 2.18 | 2.18 | 1.5 | 2.27 | 2.15 | - | - | - | - | - | - |
| *21* | **G** | exp. | 2.21 | 2.13 | 2.18 | 1.8 | 2.35 | 2.11 | - | - | - | - | - | - |
| *22* | **I** | bur. | - | - | - | - | - | - | - | - | - | - | - | - |
| *23* | **K** | exp. | - | - | - | 2.53 | - | - | - | - | - | - | - | - |
| *24* | **S** | exp. | 1.2 | 1.07 | 1.22 | 0.63 | 1.32 | 1.08 | 2.22 | 2.16 | 2.27 | 1.86 | 2.02 | 1.89 |
| *25* | **Y** | exp. | - | - | 2.43 | 2.53 | - | - | - | - | - | - | - | - |
| *26* | **L** | bur. | - | - | - | - | - | - | - | - | - | - | - | - |
| *27* | **V** | exp. | 2.68 | 2.85 | - | 2.53 | - | 2.85 | - | - | - | - | - | - |
| *28* | **S** | exp. | 0.97 | 0.88 | 1.03 | 0.75 | 1.03 | 0.95 | 1.83 | 1.85 | 1.92 | 1.69 | 1.77 | 1.6 |
| *29* | **Q** | exp. | 1.5 | 1.42 | 1.53 | 1.47 | 1.51 | 1.52 | 2.18 | 2.15 | 2.35 | 2.18 | 2.35 | 2.15 |
| *30* | **G** | exp. | 0.45 | 0.56 | 0.49 | 0.42 | 0.33 | 0.62 | - | - | - | - | 2.43 | 2.51 |
| *31* | **W** | exp. | 0.76 | 0.85 | 0.75 | 0.61 | 0.58 | 1 | - | - | - | - | - | 2.85 |
| *32* | **S** | exp. | 2.51 | 2.28 | 2.43 | 2.53 | 2.43 | 2.28 | 2.27 | 2.46 | 2.27 | 2.38 | 2.27 | 2.23 |
| *33* | **R** | exp. | 1.72 | 1.6 | 1.77 | 1.47 | 1.78 | 1.63 | - | 2.85 | - | 2.85 | - | 2.85 |
| *34* | **D** | exp. | 1.56 | 1.42 | 1.57 | 1.57 | 1.59 | 1.57 | 0.89 | 0.79 | 0.8 | 0.72 | 1.09 | 1.1 |
| *35* | **K** | exp. | - | 2.85 | 2.43 | 2.53 | - | 2.85 | - | - | - | - | - | 2.85 |
| *36* | **L** | exp. | - | - | - | - | - | - | - | - | - | - | - | - |
| *37* | **Y** | exp. | 1.97 | 1.92 | 1.82 | 1.87 | 1.77 | 2 | - | - | - | 2.85 | 2.43 | 2.43 |
| *38* | **A** | exp. | - | - | - | - | - | - | - | - | - | - | - | - |
| *39* | **V** | bur. | - | - | - | - | - | - | - | - | - | - | - | - |

| | | | | | | | | | | | | | | |
|---|---|---|---|---|---|---|---|---|---|---|---|---|---|---|
| *40* | **D** | exp. | 1.65 | 1.52 | 1.54 | 1.53 | 1.63 | 1.55 | 0.73 | 0.7 | 0.74 | 0.46 | 1.26 | 1.31 |
| *41* | **F** | exp. | - | - | - | - | - | - | - | - | - | - | - | - |
| *42* | **W** | exp. | **-0.37** | **-0.44** | **-0.37** | **-0.5** | **-0.42** | **-0.39** | - | 2.85 | - | 2.85 | 2.18 | 2.04 |
| *43* | **D** | exp. | 2.68 | 2.43 | 2.35 | 2.31 | 2.35 | 2.46 | 1.45 | 1.12 | 1.05 | 0.94 | 2.18 | 2.15 |
| *44* | **K** | exp. | 2.68 | 2.85 | 2.43 | 2.53 | 2.43 | 2.76 | - | 2.85 | - | 2.85 | - | 2.85 |
| *45* | **T** | exp. | 1.56 | 1.52 | 1.53 | 1.32 | 1.63 | 1.55 | 1.28 | 1 | 0.95 | 1.33 | 1.6 | 1.5 |
| *46* | **G** | exp. | - | 2.85 | - | - | - | - | - | - | - | - | - | - |
| *47* | **T** | exp. | 2.11 | 1.95 | 2.05 | 1.75 | 1.97 | 1.98 | 1.54 | 1.12 | 1.06 | 1.51 | 1.92 | 1.72 |
| *48* | **N** | exp. | 2.68 | 2.85 | - | - | 2.43 | - | - | - | - | - | - | - |
| *49* | **Y** | exp. | **-0.02** | **-0.06** | 0.02 | **-0.16** | 0.03 | **-0.01** | 2.43 | 2.85 | 2.43 | 2.85 | 2.43 | 2.23 |
| *50* | **N** | exp. | 1.75 | 1.59 | 1.66 | 1.47 | 1.61 | 1.59 | 2.18 | 2.01 | 2.1 | 2.11 | 2.1 | 1.89 |
| *51* | **N** | bur. | - | - | - | - | - | - | - | - | - | - | - | - |
| *52* | **G** | exp. | - | - | - | - | - | - | - | - | - | - | - | - |
| *53* | **P** | exp. | 1.61 | 1.55 | 1.71 | 1.31 | 1.7 | 1.67 | - | - | - | - | - | 2.85 |
| *54* | **V** | exp. | 2.68 | 2.85 | - | 2.53 | 2.43 | 2.71 | - | - | - | - | - | - |
| *55* | **L** | bur. | - | - | - | - | - | - | - | - | - | - | - | - |
| *56* | **S** | exp. | 2.68 | 2.76 | 2.43 | 2.53 | 2.43 | 2.76 | - | 2.85 | - | 2.85 | - | 2.85 |
| *57* | **R** | exp. | 1.9 | 1.86 | 1.97 | 1.46 | 1.97 | 1.84 | - | - | - | - | - | 2.85 |
| *58* | **F** | exp. | 2.26 | 2.33 | 2.18 | 2.28 | 2.02 | 2.33 | - | - | - | - | - | - |
| *59* | **V** | bur. | - | - | - | - | - | - | - | - | - | - | - | - |
| *60* | **Q** | exp. | 1.97 | 1.87 | 2.02 | 1.87 | 2.1 | 1.92 | 2.43 | 2.33 | 2.35 | 2.18 | 2.02 | 2.18 |
| *61* | **K** | exp. | 2.68 | 2.85 | 2.43 | 2.53 | 2.43 | 2.6 | - | - | - | - | 2.43 | 2.85 |
| *62* | **V** | bur. | - | - | - | - | - | - | - | - | - | - | - | - |
| *63* | **L** | exp. | - | - | - | - | - | 2.85 | - | - | - | - | - | - |
| *64* | **D** | exp. | 1.43 | 1.37 | 1.48 | 1.47 | 1.43 | 1.45 | 0.62 | 0.59 | 0.54 | 0.52 | 0.85 | 0.91 |
| *65* | **E** | exp. | 0.58 | 0.59 | 0.58 | 0.73 | 0.47 | 0.7 | 0.27 | 0.29 | 0.26 | 0.24 | 0.66 | 0.71 |
| *66* | **T** | exp. | - | 2.85 | - | - | - | 2.85 | - | - | - | - | - | - |
| *67* | **G** | exp. | 0.87 | 0.81 | 0.87 | 0.92 | 0.85 | 0.91 | - | 2.85 | - | 2.85 | 2.43 | 2.33 |
| *68* | **A** | exp. | - | 2.85 | - | - | - | - | - | - | - | - | - | - |
| *69* | **K** | exp. | 2.68 | 2.85 | 2.43 | - | 2.43 | 2.85 | - | - | - | - | - | 2.85 |
| *70* | **K** | bur. | - | - | - | - | - | - | - | - | - | - | - | - |
| *71* | **V** | bur. | - | - | - | - | - | - | - | - | - | - | - | - |
| *72* | **D** | bur. | - | - | - | - | - | - | 0.57 | 0.47 | 0.73 | -0.08 | 0.83 | 0.96 |
| *73* | **I** | bur. | - | - | - | - | - | - | - | - | - | - | - | - |
| *74* | **V** | bur. | - | - | - | - | - | - | - | - | - | - | - | - |
| *75* | **A** | bur. | - | - | - | - | - | - | - | - | - | - | - | - |
| *76* | **H** | bur. | - | - | - | - | - | - | - | - | - | - | - | - |
| *77* | **S** | exp. | 2.54 | 2.58 | 2.43 | 1.7 | - | 2.85 | - | - | - | - | - | - |
| *78* | **M** | exp. | 1.62 | 1.72 | 1.53 | 1.34 | 1.96 | 1.63 | - | - | - | - | - | - |
| *79* | **G** | bur. | - | - | - | - | - | - | - | - | - | - | - | - |
| *80* | **G** | bur. | - | - | - | - | - | - | - | - | - | - | - | - |
| *81* | **A** | bur. | - | - | - | - | - | - | - | - | - | - | - | - |
| *82* | **N** | bur. | - | - | - | - | - | - | - | - | - | - | - | - |
| *83* | **T** | bur. | - | - | - | - | - | - | - | - | - | - | - | - |
| *84* | **L** | bur. | - | - | - | - | - | - | - | - | - | - | - | - |
| *85* | **Y** | exp. | - | - | - | - | - | - | - | - | - | - | - | - |
| *86* | **Y** | bur. | - | - | - | - | - | - | - | - | - | - | - | - |

| | | | | | | | | | | | | | | |
|---|---|---|---|---|---|---|---|---|---|---|---|---|---|---|
| *87* | **I** | exp. | - | - | - | - | - | - | - | - | - | - | - | - |
| *88* | **K** | exp. | - | - | - | 2.53 | - | - | - | - | - | - | - | - |
| *89* | **N** | exp. | 1.78 | 1.8 | 1.73 | 1.33 | 1.77 | 1.77 | 2.43 | 2.6 | 2.43 | 2.43 | 2.43 | 2.43 |
| *90* | **L** | exp. | 2.68 | 2.85 | - | 2.53 | 2.43 | 2.85 | - | - | - | - | - | - |
| *91* | **D** | exp. | 2.26 | 2.23 | 2.43 | 2.11 | 2.35 | 2.28 | 1.46 | 1.33 | 1.33 | 1.27 | 1.82 | 1.67 |
| *92* | **G** | bur. | - | - | - | - | - | - | - | - | - | - | - | - |
| *93* | **G** | exp. | - | - | 2.43 | - | - | 2.85 | - | - | - | - | - | - |
| *94* | **N** | exp. | 1.7 | 1.6 | 1.82 | 1.72 | 1.77 | 1.69 | 1.96 | 1.87 | 2.18 | 1.87 | 2.02 | 1.95 |
| *95* | **K** | exp. | - | 2.85 | - | - | - | 2.85 | - | - | - | - | - | 2.85 |
| *96* | **V** | bur. | - | - | - | - | - | - | - | - | - | - | - | - |
| *97* | **A** | exp. | 2.68 | 2.76 | 2.43 | 2.53 | 2.43 | 2.85 | - | - | - | - | - | - |
| *98* | **N** | exp. | 2.43 | 2.41 | 2.33 | 2.53 | 2.43 | 2.85 | 2.3 | 2.68 | 2.19 | 2.35 | 1.7 | 1.87 |
| *99* | **V** | bur. | - | - | - | - | - | - | - | - | - | - | - | - |
| *100* | **V** | bur. | - | - | - | - | - | - | - | - | - | - | - | - |
| *101* | **T** | bur. | - | - | - | - | - | - | - | - | - | - | - | - |
| *102* | **L** | bur. | - | - | - | - | - | - | - | - | - | - | - | - |
| *103* | **G** | bur. | - | - | - | - | - | - | - | - | - | - | - | - |
| *104* | **G** | bur. | - | - | - | - | - | - | - | - | - | - | - | - |
| *105* | **A** | bur. | 2.54 | 2.71 | 2.43 | 2.53 | 2.43 | 2.18 | - | - | - | - | - | - |
| *106* | **N** | bur. | - | - | - | - | - | - | - | - | - | - | - | - |
| *107* | **R** | exp. | 2.68 | 2.68 | 2.43 | 2.21 | 2.43 | 2.63 | - | - | - | - | - | - |
| *108* | **L** | exp. | 1.79 | 1.73 | 1.7 | 1.54 | 1.87 | 1.82 | - | - | - | - | - | - |
| *109* | **T** | exp. | 2.68 | 2.85 | - | - | - | 2.85 | - | - | - | - | - | - |
| *110* | **T** | exp. | - | - | - | - | - | - | - | - | - | - | - | - |
| *111* | **G** | exp. | - | - | - | - | - | - | - | - | - | - | - | - |
| *112* | **K** | exp. | 2.68 | 2.85 | - | - | - | 2.85 | - | - | - | - | - | 2.85 |
| *113* | **A** | bur. | - | - | - | - | - | - | - | - | - | - | - | 2.85 |
| *114* | **L** | exp. | - | - | - | - | - | - | - | - | - | - | - | - |
| *115* | **P** | exp. | 2.68 | 2.43 | 2.27 | 2.36 | 2.43 | 2.43 | - | - | - | - | - | - |
| *116* | **G** | exp. | - | - | 2.43 | - | - | 2.85 | 1.84 | 1.57 | 0.67 | 2.49 | 2.43 | 2.85 |
| *117* | **T** | exp. | 1.58 | 1.62 | 1.59 | 1.57 | 1.63 | 1.59 | 2.43 | 2.85 | 2.33 | 2.68 | 2.43 | 2.43 |
| *118* | **D** | exp. | 2.16 | 2.11 | 2.18 | 2.28 | 2.02 | 2.1 | 1.57 | 1.47 | 1.77 | 1.23 | 1.92 | 1.87 |
| *119* | **P** | exp. | 1.44 | 1.31 | 1.4 | 1.41 | 1.38 | 1.41 | 2.43 | 2.85 | 2.43 | 2.85 | 2.43 | 2.43 |
| *120* | **N** | exp. | 0.92 | 0.83 | 1.02 | 0.97 | 0.9 | 0.94 | 1.14 | 1.13 | 1.14 | 1.06 | 1.14 | 1.1 |
| *121* | **Q** | exp. | 1.25 | 1.15 | 1.33 | 1.27 | 1.28 | 1.36 | 1.43 | 1.42 | 1.55 | 1.27 | 1.48 | 1.46 |
| *122* | **K** | exp. | 2.68 | 2.68 | 2.43 | 2.53 | 2.43 | 2.85 | - | 2.85 | - | 2.85 | 2.43 | 2.85 |
| *123* | **I** | bur. | - | - | - | - | - | - | - | - | - | - | - | - |
| *124* | **L** | exp. | 2.26 | 2.23 | 2.43 | 2.39 | 2.43 | 2.63 | - | - | - | - | - | - |
| *125* | **Y** | bur. | - | - | - | - | - | - | - | - | - | - | - | - |
| *126* | **T** | bur. | - | - | - | - | - | - | - | - | - | - | - | - |
| *127* | **S** | bur. | - | - | - | - | - | - | - | - | - | - | - | - |
| *128* | **I** | bur. | - | - | - | - | - | - | - | - | - | - | - | - |
| *129* | **Y** | exp. | - | - | - | - | - | - | - | - | - | - | - | - |
| *130* | **S** | bur. | - | - | - | - | - | - | - | - | 2.43 | 2.43 | - | - |
| *131* | **S** | exp. | 1.58 | 1.49 | 1.68 | 1.59 | 1.63 | 1.72 | 2.33 | 2.38 | 2.11 | 2.33 | 2.27 | 2.16 |
| *132* | **A** | exp. | 1.58 | 1.73 | 1.52 | 1.73 | 1.83 | 1.6 | - | - | - | - | 2.43 | 2.85 |
| *133* | **D** | exp. | 2.29 | 1.92 | 2.07 | 1.74 | 1.92 | 2.17 | 1.17 | 0.97 | 0.94 | 0.76 | 1.37 | 1.45 |

| | | | | | | | | | | | | | | |
|---|---|---|---|---|---|---|---|---|---|---|---|---|---|---|
| *134* | **M** | exp. | 0.8 | 1.17 | 0.91 | 1.12 | 1.17 | 1.13 | 2.43 | 2.85 | 2.43 | 2.57 | 2.33 | 2.51 |
| *135* | **I** | exp. | 2.06 | 1.65 | 1.72 | 1.3 | 1.77 | 1.75 | - | - | 2.43 | 2.43 | - | - |
| *136* | **V** | exp. | 1.79 | 2.48 | 2.43 | 1.94 | - | 2.85 | - | - | 2.43 | 2.85 | - | - |
| *137* | **M** | exp. | 1.15 | 1.22 | 1.14 | 0.94 | 1.15 | 1.25 | 2.43 | 2.85 | 2.43 | 2.85 | 2.43 | 2.43 |
| *138* | **N** | exp. | 2.68 | 2.85 | 2.43 | 2.53 | 2.43 | 2.74 | 2.16 | 2.43 | 2.07 | 2.11 | 1.95 | 2.34 |
| *139* | **Y** | exp. | 0.28 | 0.24 | 0.22 | 0.01 | 0.24 | 0.25 | 2.43 | 2.85 | - | 2.85 | 2.43 | 2.6 |
| *140* | **L** | exp. | - | 2.85 | - | - | - | 2.85 | - | - | - | - | - | 2.85 |
| *141* | **S** | bur. | - | - | - | - | - | - | - | - | - | - | - | - |
| *142* | **R** | exp. | 2.16 | 1.97 | 2.1 | 1.72 | 2.27 | 2 | - | - | - | - | - | - |
| *143* | **L** | bur. | - | - | - | - | - | - | - | - | - | - | - | - |
| *144* | **D** | exp. | 2.21 | 2.1 | 2.43 | 2.01 | 2.35 | 2.05 | 2 | 1.8 | 1.63 | 1.77 | 2.27 | 1.91 |
| *145* | **G** | exp. | 2.68 | 2.68 | - | 2.53 | 2.43 | 2.85 | - | - | - | - | 2.43 | 2.85 |
| *146* | **A** | exp. | - | - | - | - | - | - | - | - | - | - | - | - |
| *147* | **R** | exp. | 1.76 | 1.61 | 1.92 | 1.6 | 1.87 | 1.7 | - | - | - | - | - | - |
| *148* | **N** | exp. | - | - | - | - | - | - | - | - | - | - | - | 2.85 |
| *149* | **V** | exp. | 2.68 | 2.85 | - | 2.53 | - | 2.85 | - | - | - | - | - | - |
| *150* | **Q** | exp. | 1.62 | 1.59 | 1.71 | 1.48 | 1.73 | 1.51 | 1.84 | 2.01 | 2.05 | 1.94 | 2.05 | 2 |
| *151* | **I** | bur. | - | - | - | - | - | - | - | - | - | - | - | - |
| *152* | **H** | exp. | 0.83 | 0.81 | 0.86 | 0.78 | 0.84 | 0.85 | 2.43 | 2.55 | 2.43 | 2.41 | 2.02 | 1.89 |
| *153* | **G** | exp. | 1.5 | 1.45 | 1.63 | 1.39 | 1.56 | 1.57 | - | - | - | - | - | 2.85 |
| *154* | **V** | exp. | 2.68 | 2.85 | - | 2.53 | - | 2.85 | - | - | - | - | - | - |
| *155* | **G** | exp. | 2.68 | 2.85 | 2.43 | 2.53 | 2.43 | 2.6 | - | - | - | - | - | - |
| *156* | **H** | exp. | 2.18 | 2.71 | 2.43 | 1.67 | 2.43 | 2.18 | - | - | - | 2.85 | 2.43 | - |
| *157* | **I** | exp. | 1.88 | 1.9 | 1.95 | 1.63 | 1.82 | 1.9 | - | - | - | - | - | - |
| *158* | **G** | exp. | 2.68 | 2.85 | 2.35 | 2.53 | 2.43 | 2.85 | - | - | - | - | - | - |
| *159* | **L** | bur. | - | - | - | - | - | - | - | - | - | - | - | - |
| *160* | **L** | bur. | - | - | - | - | - | - | - | - | - | - | - | - |
| *161* | **Y** | exp. | 0.11 | 0.14 | 0.21 | **-0.08** | 0.04 | 0.15 | - | - | - | - | 2.43 | 2.85 |
| *162* | **S** | exp. | - | - | - | 2.53 | - | 2.85 | - | - | - | - | - | - |
| *163* | **S** | exp. | 1.36 | 1.29 | 1.35 | 1.11 | 1.43 | 1.32 | 2.27 | 1.95 | 2.18 | 1.98 | 2.02 | 1.86 |
| *164* | **Q** | exp. | 1.14 | 1.01 | 1.15 | 0.94 | 1.15 | 1.12 | 2.02 | 1.95 | 1.97 | 1.85 | 1.97 | 1.84 |
| *165* | **V** | bur. | - | - | - | - | - | - | - | - | - | - | - | - |
| *166* | **N** | exp. | - | - | 2.43 | 2.53 | - | 2.85 | - | - | - | - | - | - |
| *167* | **S** | exp. | 1.43 | 1.31 | 1.53 | 1.27 | 1.46 | 1.39 | 2.43 | 2.38 | 2.43 | 2.23 | 2.27 | 2.15 |
| *168* | **L** | exp. | 2.68 | 2.6 | 2.43 | 2.53 | 2.43 | 2.68 | - | - | - | - | - | - |
| *169* | **I** | bur. | - | - | - | - | - | - | - | - | - | - | - | - |
| *170* | **K** | exp. | 2.68 | 2.85 | - | 2.53 | 2.43 | 2.85 | - | - | - | - | 2.43 | 2.85 |
| *171* | **E** | exp. | 1.72 | 1.62 | 1.77 | 1.69 | 1.73 | 1.73 | 1.09 | 0.99 | 1 | 0.93 | 1.43 | 1.38 |
| *172* | **G** | bur. | - | - | - | - | - | - | - | - | - | - | - | - |
| *173* | **L** | exp. | - | - | - | - | - | - | - | - | - | - | - | - |
| *174* | **N** | exp. | 0.41 | 0.4 | 0.33 | 0.26 | 0.32 | 0.49 | 1.45 | 1.58 | 1.36 | 1.55 | 1.26 | 1.32 |
| *175* | **G** | exp. | 1.1 | 1.14 | 1.09 | 1.15 | 0.99 | 1.26 | - | - | - | - | 2.43 | 2.85 |
| *176* | **G** | exp. | 1.56 | 1.5 | 1.53 | 1.61 | 1.46 | 1.62 | - | - | - | - | 2.43 | 2.85 |
| *177* | **G** | exp. | - | - | - | 2.53 | - | - | - | - | - | - | - | - |
| *178* | **Q** | exp. | 1.36 | 1.1 | 1.43 | 1.3 | 1.37 | 1.32 | 1.41 | 1.21 | 1.36 | 1.19 | 1.3 | 1.31 |
| *179* | **N** | exp. | 2.68 | - | - | 1.86 | - | - | 2.06 | 2.29 | 2.1 | 2.01 | 2.13 | 1.91 |
| *180* | **T** | exp. | 1.8 | 1.98 | 1.92 | 1.7 | 1.97 | 1.85 | 2.43 | 2.11 | 2.35 | 2.28 | 1.97 | 1.93 |

| | | | | | | | | | | | | | |
|---|---|---|---|---|---|---|---|---|---|---|---|---|---|
| *181* | **N** | exp. | 1.61 | 1.17 | 1.57 | 1.55 | 1.58 | 1.51 | 1.3 | 1.16 | 1.12 | 1.25 | 1.24 | 1.18 |

[a] In kcal $mol^{-1}$ for 1 M standard state. The extended MD simulation times of 2 µs per replica lead to a SEM < 0.25 kcal $mol^{-1}$ for 96% (486/505 over all solvents) of all observed interactions with binding free energies ≤ 2.0 kcal $mol^{-1}$. Negative binding free energies are highlighted in bold.
[b] Relative solvent accessibility: bur.: buried (< 5%), exp.: exposed (≥ 5%).
[c] Concentration in M.
[d] n.d.: not determined; Residues A1 & E2 are missing in the PDB structure of 1I6W.
[e] -: A dash indicates that no interactions were observed in our simulations.

## 1.2 Binding free energies[a] for all anion interactions with *Bs*LipA residues

**Table S2: Binding free energies[a] for anion interactions with *Bs*LipA residues**

| *ID* | *AA* | *SA*[b] | *MD01* | *MD02* | *MD03* | *MD04* | *MD05* | *MD06* | *MD07* | *MD08* | *MD09* | *MD10* | *MD11* | *MD12* |
|---|---|---|---|---|---|---|---|---|---|---|---|---|---|---|
| *Cation* | - | - | [BMIM$^+$] | [BMIM$^+$] | [BMIM$^+$] | [BMIM$^+$] | [BMIM$^+$] | [BMIM$^+$] | [Na$^+$] | [Na$^+$] | [Na$^+$] | [Na$^+$] | [K$^+$] | [K$^+$] |
| *Anion* | - | - | [Br$^-$] | [Cl$^-$] | [I$^-$] | [TfO$^-$] | [Cl$^-$] | [I$^-$] | [Cl$^-$] | [Cl$^-$] | [I$^-$] | [I$^-$] | [Cl$^-$] | [Cl$^-$] |
| *C*[c] | - | - | 0.9 | 1.2 | 0.6 | 0.7 | 0.6 | 1.2 | 0.6 | 1.2 | 0.6 | 1.2 | 0.6 | 1.2 |
| *1* | **A** | exp. | n.d.[d] | n.d. | n.d. | n.d. | n.d. | n.d. | n.d. | n.d. | n.d. | n.d. | n.d. | n.d. |
| *2* | **E** | exp. | n.d. | n.d. | n.d. | n.d. | n.d. | n.d. | n.d. | n.d. | n.d. | n.d. | n.d. | n.d. |
| *3* | **H** | exp. | 1.8 | 2.2 | 1.66 | 1.14 | 2.43 | 1.59 | 2.43 | 2.51 | 1.78 | 1.75 | 2.43 | 2.38 |
| *4* | **N** | exp. | 2.26 | 2.43 | 2.02 | 1.1 | 2.43 | 1.85 | 2.43 | 2.38 | 1.73 | 1.48 | 2.43 | 2.38 |
| *5* | **P** | bur. | -[e] | - | - | 2.03 | - | - | - | - | - | - | - | - |
| *6* | **V** | bur. | - | - | - | - | - | - | - | - | - | - | - | - |
| *7* | **V** | bur. | - | - | - | - | - | - | - | - | - | - | - | - |
| *8* | **M** | bur. | - | - | - | 2.53 | - | - | - | - | - | - | - | - |
| *9* | **V** | bur. | - | - | - | - | - | - | - | - | - | - | - | - |
| *10* | **H** | bur. | - | - | - | - | - | - | - | - | - | - | - | - |
| *11* | **G** | exp. | - | - | - | 2.39 | - | 2.85 | - | 2.85 | 2.43 | 2.85 | - | - |
| *12* | **I** | exp. | 2.29 | 2.76 | 1.97 | 0.79 | 2.43 | 1.86 | - | 2.85 | 1.87 | 1.85 | - | 2.85 |
| *13* | **G** | exp. | 1.8 | 2.18 | 1.54 | 0.69 | 2.02 | 1.58 | 2.1 | 2.05 | 1.43 | 1.43 | 2.27 | 2.15 |
| *14* | **G** | exp. | 2.68 | 2.85 | 2.43 | 1.4 | - | 2.38 | - | 2.85 | 2.35 | 2.28 | - | 2.85 |
| *15* | **A** | exp. | 2.26 | 2.51 | 1.83 | 1.3 | 2.43 | 1.95 | 2.43 | 2.38 | 1.92 | 1.67 | 2.35 | 2.43 |
| *16* | **S** | exp. | 2.16 | 2.28 | 1.76 | 0.98 | 2.43 | 2 | 2.43 | 2.19 | 1.82 | 1.6 | 2.3 | 2.43 |
| *17* | **F** | exp. | 2.51 | 2.85 | 2.02 | 1.13 | 2.43 | 2.07 | 2.43 | 2.85 | 1.92 | 1.89 | 2.43 | 2.85 |
| *18* | **N** | exp. | 2.21 | 2.43 | 1.95 | 0.5 | 2.43 | 1.75 | 2.43 | 2.68 | 1.97 | 1.84 | 2.43 | 2.51 |
| *19* | **F** | bur. | - | - | - | 2.53 | - | - | - | - | - | - | - | - |
| *20* | **A** | exp. | 1.2 | 1.55 | 0.89 | **-0.41** | 1.59 | 0.84 | 1.7 | 1.73 | 0.87 | 0.92 | 1.7 | 1.73 |
| *21* | **G** | exp. | 1.87 | 2.18 | 1.54 | 0.47 | 2.27 | 1.43 | 2.43 | 2.43 | 1.51 | 1.52 | 2.43 | 2.43 |
| *22* | **I** | bur. | - | - | - | - | - | - | - | - | - | - | - | - |
| *23* | **K** | exp. | 0.77 | 0.85 | 0.68 | **-0.44** | 0.95 | 0.64 | 1.15 | 1.12 | 0.66 | 0.59 | 1.17 | 1.18 |
| *24* | **S** | exp. | 1.18 | 1.33 | 0.99 | **-0.27** | 1.34 | 1.01 | 1.45 | 1.45 | 0.99 | 1.02 | 1.45 | 1.49 |
| *25* | **Y** | exp. | 2.68 | 2.85 | 2.43 | 1.55 | - | 2.43 | - | 2.85 | 2.43 | 2.38 | - | 2.85 |
| *26* | **L** | bur. | - | - | - | - | - | - | - | - | - | - | - | - |
| *27* | **V** | exp. | 2.26 | 2.85 | 1.63 | **-0.16** | 2.43 | 1.67 | - | 2.85 | 1.59 | 1.65 | 2.43 | 2.85 |
| *28* | **S** | exp. | 1.39 | 1.58 | 1.19 | 0.29 | 1.63 | 1.2 | 1.59 | 1.6 | 1.15 | 1.13 | 1.63 | 1.62 |
| *29* | **Q** | exp. | 1.3 | 1.46 | 1.17 | 0.8 | 1.59 | 1.14 | 1.63 | 1.56 | 1.19 | 1.12 | 1.59 | 1.6 |
| *30* | **G** | exp. | 2.26 | 2.51 | 1.87 | 0.88 | 2.43 | 1.86 | 2.43 | 2.43 | 2.02 | 1.75 | 2.43 | 2.38 |
| *31* | **W** | exp. | - | - | - | 2.44 | - | - | - | - | - | - | - | - |
| *32* | **S** | exp. | 1.43 | 1.49 | 1.43 | 0.94 | 1.59 | 1.36 | 1.73 | 1.55 | 1.34 | 1.16 | 1.66 | 1.65 |
| *33* | **R** | exp. | 0.87 | 0.99 | 0.8 | 0.08 | 1.06 | 0.78 | 1.14 | 1.1 | 0.7 | 0.67 | 1.12 | 1.13 |
| *34* | **D** | exp. | 2.01 | 2.07 | 2.02 | 1.68 | 2.02 | 1.95 | 2.02 | 1.95 | 1.92 | 1.85 | 2.02 | 1.95 |
| *35* | **K** | exp. | 1.12 | 1.23 | 1.12 | 0.41 | 1.36 | 1.07 | 1.48 | 1.38 | 1.14 | 0.94 | 1.51 | 1.47 |
| *36* | **L** | exp. | - | - | - | 2.18 | - | - | - | - | - | - | - | - |
| *37* | **Y** | exp. | - | - | - | 0.81 | - | 2.85 | - | 2.85 | - | 2.85 | - | - |
| *38* | **A** | exp. | 2.59 | 2.85 | 1.97 | 0.29 | - | 2.15 | 2.43 | 2.76 | 2.1 | 1.85 | 2.43 | 2.85 |
| *39* | **V** | bur. | - | - | - | - | - | - | - | - | - | - | - | - |
| *40* | **D** | exp. | 2.68 | 2.85 | 2.43 | 2.44 | - | 2.85 | - | 2.85 | 2.43 | 2.51 | 2.43 | 2.85 |
| *41* | **F** | exp. | - | - | - | - | - | - | - | - | - | - | - | - |

| | | | | | | | | | | | | | | |
|---|---|---|---|---|---|---|---|---|---|---|---|---|---|---|
| *42* | **W** | exp. | 2.59 | 2.85 | 2.1 | 1.39 | 2.43 | 2.18 | 2.43 | 2.68 | 2.1 | 2.11 | 2.43 | 2.68 |
| *43* | **D** | exp. | 2.68 | 2.85 | 2.43 | 1.74 | 2.43 | 2.6 | 2.43 | 2.85 | 2.43 | 2.34 | 2.43 | 2.85 |
| *44* | **K** | exp. | 0.71 | 0.78 | 0.69 | 0.61 | 0.88 | 0.65 | 1.01 | 0.95 | 0.69 | 0.61 | 1.06 | 1 |
| *45* | **T** | exp. | 2.01 | 2.43 | 1.77 | 0.98 | 2.43 | 1.8 | 2.43 | 2.11 | 1.7 | 1.7 | 2.43 | 2.38 |
| *46* | **G** | exp. | - | 2.85 | - | 1.67 | - | - | - | - | - | 1.75 | - | - |
| *47* | **T** | exp. | 2.01 | 2.38 | 1.82 | 1.3 | 2.43 | 1.76 | 2.43 | 2.33 | 1.87 | 1.62 | 2.43 | 2.43 |
| *48* | **N** | exp. | 1.94 | 2.38 | 1.59 | **-0.06** | 2.43 | 1.64 | 2.43 | 2.85 | 1.77 | 1.75 | 2.43 | 2.85 |
| *49* | **Y** | exp. | 1.67 | 1.86 | 1.45 | 0.79 | 1.82 | 1.52 | 1.97 | 1.86 | 1.48 | 1.43 | 1.97 | 1.89 |
| *50* | **N** | exp. | 1.83 | 1.9 | 1.45 | 0.8 | 1.92 | 1.46 | 2.02 | 1.95 | 1.59 | 1.53 | 2.1 | 1.95 |
| *51* | **N** | bur. | - | - | - | 2.53 | - | - | - | - | - | - | - | - |
| *52* | **G** | exp. | - | - | - | - | - | - | - | - | - | - | - | - |
| *53* | **P** | exp. | 2.01 | 2.43 | 1.59 | 0.08 | 2.43 | 1.6 | 2.43 | 2.43 | 1.56 | 1.55 | 2.43 | 2.43 |
| *54* | **V** | exp. | 2.21 | 2.68 | 1.88 | 0.4 | 2.43 | 1.86 | 2.43 | 2.85 | 2.1 | 1.95 | 2.43 | 2.85 |
| *55* | **L** | bur. | - | - | - | - | - | - | - | - | - | - | - | - |
| *56* | **S** | exp. | 2.26 | 2.43 | 1.92 | 0.32 | 2.43 | 1.9 | 2.43 | 2.43 | 1.77 | 1.63 | 2.43 | 2.43 |
| *57* | **R** | exp. | 0.88 | 1.03 | 0.8 | **-0.09** | 1.11 | 0.78 | 1.34 | 1.29 | 0.87 | 0.84 | 1.34 | 1.31 |
| *58* | **F** | exp. | - | - | - | 0.69 | - | - | - | - | - | 2.85 | - | - |
| *59* | **V** | bur. | - | - | - | - | - | - | - | - | - | - | - | - |
| *60* | **Q** | exp. | 1.83 | 2.04 | 1.77 | 1.15 | 2.02 | 1.77 | 2.02 | 1.89 | 1.59 | 1.47 | 2.02 | 1.92 |
| *61* | **K** | exp. | 0.73 | 0.8 | 0.73 | 0.06 | 0.95 | 0.63 | 1.09 | 1.05 | 0.76 | 0.64 | 1.15 | 1.07 |
| *62* | **V** | bur. | - | - | - | 1 | - | - | - | - | - | - | - | - |
| *63* | **L** | exp. | - | - | - | 2.06 | - | - | - | - | - | - | - | - |
| *64* | **D** | exp. | 2.59 | 2.6 | 2.43 | 1.92 | 2.43 | 2.38 | 2.43 | 2.28 | 2.27 | 2.07 | 2.43 | 2.33 |
| *65* | **E** | exp. | 2.26 | 2.28 | 2.02 | 1.79 | 2.43 | 2.08 | 2.1 | 2.01 | 2.02 | 1.87 | 2.18 | 2.04 |
| *66* | **T** | exp. | - | 2.85 | - | 1.55 | - | 2.85 | - | 2.85 | 2.43 | 2.76 | - | 2.85 |
| *67* | **G** | exp. | 2.26 | 2.51 | 2.02 | 1.31 | 2.43 | 2 | 2.35 | 2.11 | 1.82 | 1.71 | 2.35 | 2.15 |
| *68* | **A** | exp. | 2.26 | 2.51 | 2.27 | 0.86 | 2.43 | 2.07 | 2.43 | 2.43 | 1.97 | 1.64 | 2.43 | 2.68 |
| *69* | **K** | exp. | 0.75 | 0.8 | 0.77 | 0.68 | 0.94 | 0.7 | 0.97 | 0.9 | 0.67 | 0.54 | 1.01 | 0.97 |
| *70* | **K** | bur. | 2.34 | 2.43 | 1.99 | 1.25 | 2.43 | 2.02 | 2.43 | 2.43 | 1.78 | 1.6 | 2.43 | 2.51 |
| *71* | **V** | bur. | - | - | - | - | - | - | - | - | - | - | - | - |
| *72* | **D** | bur. | - | - | - | - | - | - | - | 2.85 | - | 2.85 | - | - |
| *73* | **I** | bur. | - | - | - | - | - | - | - | - | - | - | - | - |
| *74* | **V** | bur. | - | - | - | - | - | - | - | - | - | - | - | - |
| *75* | **A** | bur. | - | - | - | - | - | - | - | - | - | - | - | - |
| *76* | **H** | bur. | - | - | - | 1.64 | - | 1.86 | - | - | - | - | - | - |
| *77* | **S** | exp. | 2.01 | 2.03 | 2.07 | 0.71 | 2.16 | 1.98 | 2.3 | 2.71 | 1.83 | 1.71 | 2.43 | 2.51 |
| *78* | **M** | exp. | 2.68 | 2.85 | 1.89 | 0.78 | - | 2.48 | - | - | 2.23 | 2.33 | - | - |
| *79* | **G** | bur. | - | - | - | - | - | - | - | - | - | - | - | - |
| *80* | **G** | bur. | - | - | - | - | - | - | - | - | - | - | - | - |
| *81* | **A** | bur. | - | - | - | 1.78 | - | 2.85 | - | - | 2.43 | 2.64 | - | - |
| *82* | **N** | bur. | - | - | - | - | - | - | - | - | - | - | - | - |
| *83* | **T** | bur. | - | - | - | - | - | - | - | - | - | - | - | - |
| *84* | **L** | bur. | - | - | - | - | - | - | - | - | - | - | - | - |
| *85* | **Y** | exp. | 2.11 | 2.6 | 1.75 | 0.99 | 2.43 | 1.73 | 2.43 | 2.85 | 1.92 | 1.95 | 2.43 | 2.85 |
| *86* | **Y** | bur. | - | - | - | - | - | - | - | - | - | - | - | - |
| *87* | **I** | exp. | - | - | - | - | - | - | - | - | - | - | - | - |
| *88* | **K** | exp. | 0.61 | 0.78 | 0.5 | 0.06 | 0.85 | 0.52 | 0.99 | 1 | 0.5 | 0.5 | 0.98 | 1.02 |

| | | | | | | | | | | | | | | |
|---|---|---|---|---|---|---|---|---|---|---|---|---|---|---|
| *89* | **N** | exp. | 1.1 | 1.38 | 0.9 | 0.5 | 1.46 | 0.91 | 1.63 | 1.56 | 0.98 | 1 | 1.59 | 1.63 |
| *90* | **L** | exp. | 2.68 | - | 2.35 | 0.82 | - | 2.33 | - | 2.85 | 2.35 | 2.18 | - | 2.85 |
| *91* | **D** | exp. | 2.16 | 2.23 | 2.02 | 1.68 | 2.43 | 2.15 | 2.43 | 2.28 | 2.02 | 1.89 | 2.43 | 2.23 |
| *92* | **G** | bur. | - | - | - | - | - | - | - | - | - | - | - | - |
| *93* | **G** | exp. | - | - | - | 0.37 | - | 2.85 | - | - | - | 2.85 | - | - |
| *94* | **N** | exp. | 1.62 | 1.82 | 1.56 | 0.97 | 1.87 | 1.46 | 1.77 | 1.8 | 1.36 | 1.27 | 1.97 | 1.87 |
| *95* | **K** | exp. | 1.58 | 1.67 | 1.59 | 1.03 | 1.77 | 1.49 | 1.77 | 1.67 | 1.38 | 1.32 | 1.82 | 1.75 |
| *96* | **V** | bur. | - | - | - | - | - | - | - | - | - | - | - | - |
| *97* | **A** | exp. | 2.68 | 2.85 | 2.43 | 1.66 | - | 2.38 | - | 2.68 | 2.08 | 1.71 | - | 2.85 |
| *98* | **N** | exp. | - | - | - | 2.42 | - | - | - | 2.85 | 2.43 | 2.68 | - | 2.85 |
| *99* | **V** | bur. | - | - | - | - | - | - | - | - | - | - | - | - |
| *100* | **V** | bur. | - | - | - | - | - | - | - | - | - | - | - | - |
| *101* | **T** | bur. | - | - | - | - | - | - | - | - | - | - | - | - |
| *102* | **L** | bur. | - | - | - | - | - | - | - | - | - | - | - | - |
| *103* | **G** | bur. | - | 2.85 | - | - | - | - | - | - | 1.24 | 1.8 | - | - |
| *104* | **G** | bur. | 2.4 | 2.36 | 1.8 | 2.19 | - | 1.65 | - | - | 1.5 | 1.63 | - | - |
| *105* | **A** | bur. | 1.97 | 2.15 | 2.01 | 0.59 | 2.43 | 2.25 | 2.43 | 2.85 | 1.67 | 1.49 | 2.43 | 2.85 |
| *106* | **N** | bur. | - | - | - | - | - | - | - | - | - | - | - | - |
| *107* | **R** | exp. | 1.66 | 1.87 | 1.5 | 0.75 | 2.03 | 1.5 | 1.92 | 2.15 | 1.5 | 1.67 | 2.1 | 2.05 |
| *108* | **L** | exp. | 2.16 | 2.85 | 1.63 | **-0.32** | 2.43 | 1.67 | 2.43 | 2.85 | 1.7 | 1.68 | 2.43 | 2.85 |
| *109* | **T** | exp. | 1.9 | 2.35 | 1.5 | 0.93 | 2.43 | 1.56 | 2.43 | 2.68 | 1.76 | 1.78 | 2.43 | 2.64 |
| *110* | **T** | exp. | 1.78 | 2.23 | 1.51 | 1.04 | 2.43 | 1.62 | 2.43 | 2.43 | 1.48 | 1.55 | 2.43 | 2.38 |
| *111* | **G** | exp. | 1.52 | 1.77 | 1.32 | 0.66 | 1.78 | 1.38 | 1.92 | 1.98 | 1.3 | 1.31 | 2.02 | 1.95 |
| *112* | **K** | exp. | 0.69 | 0.79 | 0.64 | 0.44 | 0.88 | 0.67 | 0.97 | 0.91 | 0.5 | 0.52 | 0.92 | 0.94 |
| *113* | **A** | bur. | - | - | - | 0.87 | - | - | - | - | - | - | - | - |
| *114* | **L** | exp. | 2.68 | - | 2.18 | **-0.19** | - | 2.28 | - | - | 1.92 | 1.95 | - | - |
| *115* | **P** | exp. | 1.83 | 2.15 | 1.43 | 0.69 | 2.18 | 1.45 | 2.1 | 2.07 | 1.21 | 1.29 | 2.1 | 2.23 |
| *116* | **G** | exp. | - | - | 2.43 | **-0.16** | - | 2.6 | - | - | 2.27 | 2.38 | - | 2.85 |
| *117* | **T** | exp. | 2.01 | 2.33 | 1.82 | 0.9 | 2.43 | 1.78 | 2.35 | 2.18 | 1.73 | 1.69 | 2.43 | 2.28 |
| *118* | **D** | exp. | 2.26 | 2.18 | 2.1 | 1.7 | 2.35 | 2.15 | 2.27 | 2.2 | 2.02 | 2 | 2.18 | 2.15 |
| *119* | **P** | exp. | 1.58 | 1.84 | 1.3 | 0.8 | 1.97 | 1.28 | 1.73 | 1.69 | 1.19 | 1.17 | 1.73 | 1.71 |
| *120* | **N** | exp. | 1.4 | 1.55 | 1.26 | 0.85 | 1.59 | 1.23 | 1.53 | 1.46 | 1.17 | 1.1 | 1.51 | 1.45 |
| *121* | **Q** | exp. | 1.65 | 1.84 | 1.48 | 0.95 | 1.92 | 1.48 | 1.87 | 1.76 | 1.42 | 1.16 | 1.87 | 1.75 |
| *122* | **K** | exp. | 1.29 | 1.35 | 1.23 | 0.78 | 1.4 | 1.13 | 1.51 | 1.29 | 1 | 1.01 | 1.43 | 1.38 |
| *123* | **I** | bur. | - | - | - | - | - | - | - | - | - | - | - | - |
| *124* | **L** | exp. | - | - | - | 1.5 | - | - | - | - | - | 2.85 | - | - |
| *125* | **Y** | bur. | - | - | - | - | - | - | - | - | - | - | - | - |
| *126* | **T** | bur. | - | - | - | 2.02 | - | - | - | - | - | - | - | - |
| *127* | **S** | bur. | - | - | - | - | - | - | - | - | - | - | - | - |
| *128* | **I** | bur. | - | - | - | - | - | - | - | - | - | - | - | - |
| *129* | **Y** | exp. | 2.51 | 2.85 | 2.18 | 0.97 | 2.43 | 2.55 | - | 2.85 | 2.33 | 2.33 | - | 2.85 |
| *130* | **S** | bur. | - | 2.85 | - | 2.39 | - | - | 2.43 | - | 1.41 | 1.75 | - | - |
| *131* | **S** | exp. | 1.96 | 2.11 | 1.7 | 0.85 | 2.22 | 1.81 | 2.22 | 2.15 | 2.01 | 1.87 | 2.18 | 2.16 |
| *132* | **A** | exp. | 1.92 | 2.08 | 1.52 | 0.71 | 2.27 | 1.46 | 2.13 | 2.04 | 1.42 | 1.46 | 2.1 | 2.13 |
| *133* | **D** | exp. | 2.51 | 2.36 | 2.16 | 1.87 | 2.43 | 2.41 | 2.35 | 2.33 | 2.13 | 1.99 | 2.43 | 2.47 |
| *134* | **M** | exp. | 2.19 | 2.68 | 1.83 | 0.6 | 2.43 | 1.91 | 2.22 | 2.68 | 1.73 | 1.81 | 2.43 | 2.6 |
| *135* | **I** | exp. | 2.24 | 2.55 | 1.88 | 0.52 | 2.43 | 1.84 | 2.43 | 2.76 | 1.45 | 1.51 | 2.43 | 2.76 |

| | | | | | | | | | | | | | | |
|---|---|---|---|---|---|---|---|---|---|---|---|---|---|---|
| *136* | **V** | exp. | 2.09 | 2.57 | 2.35 | 0.5 | - | 2.24 | 2.23 | - | 1.51 | 1.8 | 2.43 | 2.85 |
| *137* | **M** | exp. | 1.97 | 2.28 | 1.46 | 0.06 | 2.35 | 1.52 | 2.33 | 2.16 | 1.6 | 1.45 | 2.27 | 2.17 |
| *138* | **N** | exp. | 2.07 | 2.55 | 1.8 | 0.91 | 2.43 | 2.11 | 2.43 | 2.43 | 1.93 | 1.93 | 2.16 | 2.6 |
| *139* | **Y** | exp. | 1.88 | 2.04 | 1.56 | 0.73 | 2.02 | 1.64 | 2.13 | 1.98 | 1.68 | 1.62 | 2.02 | 2 |
| *140* | **L** | exp. | 2.68 | 2.85 | 2.43 | 0.41 | - | 2.38 | - | 2.85 | 2.06 | 2.13 | - | 2.85 |
| *141* | **S** | bur. | - | 2.43 | - | 1.93 | - | - | - | - | 1.37 | 2 | - | - |
| *142* | **R** | exp. | 0.99 | 1.15 | 0.88 | 0.23 | 1.22 | 0.94 | 1.39 | 1.26 | 1.03 | 0.86 | 1.41 | 1.33 |
| *143* | **L** | bur. | - | - | - | - | - | - | - | - | - | - | - | - |
| *144* | **D** | exp. | 2.01 | 2.18 | 1.97 | 1.55 | 2.27 | 2.04 | 2.35 | 2.18 | 2.02 | 1.89 | 2.18 | 2.18 |
| *145* | **G** | exp. | 2.34 | 2.46 | 2.13 | 1.17 | 2.43 | 2.17 | 2.43 | 2.76 | 2.18 | 1.85 | 2.43 | 2.76 |
| *146* | **A** | exp. | - | - | - | - | - | - | - | - | - | - | - | - |
| *147* | **R** | exp. | 1.56 | 1.69 | 1.53 | 0.76 | 2.02 | 1.42 | 1.92 | 1.82 | 1.48 | 1.32 | 1.92 | 1.84 |
| *148* | **N** | exp. | 2.68 | 2.85 | 2.43 | 1.77 | - | 2.51 | - | 2.85 | 2.43 | 2.43 | 2.43 | 2.85 |
| *149* | **V** | exp. | 2.26 | 2.85 | 1.78 | 0.75 | 2.43 | 1.82 | 2.43 | 2.85 | 1.77 | 1.73 | 2.43 | 2.85 |
| *150* | **Q** | exp. | 1.3 | 1.6 | 1.12 | 0.46 | 1.63 | 1.16 | 1.83 | 1.69 | 1.28 | 1.2 | 1.73 | 1.7 |
| *151* | **I** | bur. | - | - | 2.43 | 1.05 | - | 2.76 | - | - | 2.43 | 2.85 | - | - |
| *152* | **H** | exp. | 1.5 | 1.86 | 1.22 | 0.8 | 1.82 | 1.24 | 1.97 | 1.86 | 1.39 | 1.3 | 1.92 | 1.89 |
| *153* | **G** | exp. | 1.6 | 1.87 | 1.36 | 0.51 | 2.05 | 1.3 | 1.97 | 1.9 | 1.28 | 1.16 | 1.97 | 1.87 |
| *154* | **V** | exp. | 2.59 | 2.85 | 2.1 | 0.83 | - | 2.2 | - | 2.85 | 2.43 | 2.07 | - | 2.85 |
| *155* | **G** | exp. | 1.9 | 2 | 1.58 | 1.15 | 2.35 | 1.76 | 2.01 | 2.33 | 1.46 | 1.48 | 2.08 | 2.32 |
| *156* | **H** | exp. | 2.54 | 2.43 | 2.23 | 1.4 | 2.43 | 2.38 | 2.43 | 2.85 | 1.56 | 1.89 | 2.43 | - |
| *157* | **I** | exp. | 1.78 | 2.21 | 1.46 | 0.06 | 2.43 | 1.46 | 2.43 | 2.43 | 1.41 | 1.41 | 2.43 | 2.38 |
| *158* | **G** | exp. | 1.84 | 2.28 | 1.27 | **-0.16** | 2.43 | 1.31 | 2.43 | 2.28 | 1.4 | 1.3 | 2.35 | 2.33 |
| *159* | **L** | bur. | - | - | - | - | - | - | - | - | - | - | - | - |
| *160* | **L** | bur. | - | - | - | 2.19 | - | - | - | - | - | - | - | - |
| *161* | **Y** | exp. | 2.51 | 2.85 | 2.02 | 0.9 | 2.43 | 2.07 | 2.43 | 2.85 | 2.02 | 2 | 2.43 | 2.85 |
| *162* | **S** | exp. | 2.06 | 2.28 | 1.97 | 0.6 | 2.43 | 1.98 | 2.43 | 2.43 | 2.1 | 2.07 | 2.43 | 2.43 |
| *163* | **S** | exp. | 0.96 | 1.09 | 0.92 | 0.39 | 1.15 | 0.91 | 1.26 | 1.22 | 0.93 | 0.91 | 1.26 | 1.25 |
| *164* | **Q** | exp. | 1.43 | 1.69 | 1.24 | 0.52 | 1.77 | 1.21 | 1.82 | 1.73 | 1.24 | 1.2 | 1.82 | 1.75 |
| *165* | **V** | bur. | - | - | - | 2.53 | - | - | - | - | - | - | - | - |
| *166* | **N** | exp. | 2.68 | - | 2.43 | 1.81 | - | 2.85 | - | - | 2.43 | 2.85 | - | - |
| *167* | **S** | exp. | 1.38 | 1.49 | 1.36 | 0.51 | 1.56 | 1.32 | 1.59 | 1.56 | 1.3 | 1.24 | 1.59 | 1.56 |
| *168* | **L** | exp. | - | - | 2.43 | 0.76 | - | 2.85 | - | - | 2.43 | 2.68 | - | - |
| *169* | **I** | bur. | - | - | - | - | - | - | - | - | - | - | - | - |
| *170* | **K** | exp. | 0.99 | 1.01 | 1.03 | 0.59 | 1.17 | 0.95 | 1.34 | 1.24 | 1.06 | 0.92 | 1.34 | 1.27 |
| *171* | **E** | exp. | 2.16 | 2.23 | 2.1 | 1.37 | 2.43 | 2.11 | 2.43 | 2.23 | 2.02 | 1.98 | 2.43 | 2.23 |
| *172* | **G** | bur. | - | - | - | 2.16 | - | - | - | - | - | - | - | - |
| *173* | **L** | exp. | - | - | - | - | - | - | - | - | - | - | - | - |
| *174* | **N** | exp. | 1.9 | 2.18 | 1.73 | 0.72 | 2.35 | 1.67 | 2.43 | 2.15 | 1.82 | 1.54 | 2.43 | 2.23 |
| *175* | **G** | exp. | 2.68 | - | 2.43 | 1.33 | - | 2.6 | - | 2.68 | 2.43 | 2.01 | - | 2.85 |
| *176* | **G** | exp. | 2.68 | - | 2.43 | 1.36 | - | 2.85 | - | 2.85 | 2.43 | 2.43 | - | 2.85 |
| *177* | **G** | exp. | - | - | - | 1.17 | - | - | - | - | - | - | - | - |
| *178* | **Q** | exp. | 1.73 | 1.85 | 1.7 | 1.1 | 2.1 | 1.55 | 1.87 | 1.86 | 1.51 | 1.37 | 1.92 | 1.87 |
| *179* | **N** | exp. | 2.68 | 2.85 | 2.43 | 1.71 | - | 2.85 | - | 2.85 | - | 2.68 | 2.43 | 2.85 |
| *180* | **T** | exp. | 1.99 | 2.15 | 1.77 | 1.05 | 2.18 | 1.71 | 2.1 | 1.95 | 1.56 | 1.45 | 2.02 | 2.06 |
| *181* | **N** | exp. | 1.8 | 1.8 | 1.72 | 1.17 | 2.1 | 1.74 | 1.97 | 1.9 | 1.48 | 1.61 | 1.92 | 1.84 |

[a] In kcal $mol^{-1}$ for 1 M standard state. The extended MD simulation times of 2 µs per replica lead to a SEM < 0.25 kcal $mol^{-1}$ for 96% (702/730 over all solvents) of all observed interactions with final binding free energies ≤ 2.0 kcal $mol^{-1}$. Negative binding free energies are highlighted in bold.
[b] Relative solvent accessibility: bur.: buried (< 5%), exp.: exposed (≥ 5%).
[c] Concentration in M.
[d] n.d.: not determined; Residues A1 & E2 are missing in the PDB structure of 1I6W.
[e] A dash indicates that no interactions were observed in our simulations.

# 2 Supplementary Figures

## 2.1 Influence of water model and ion parametrization

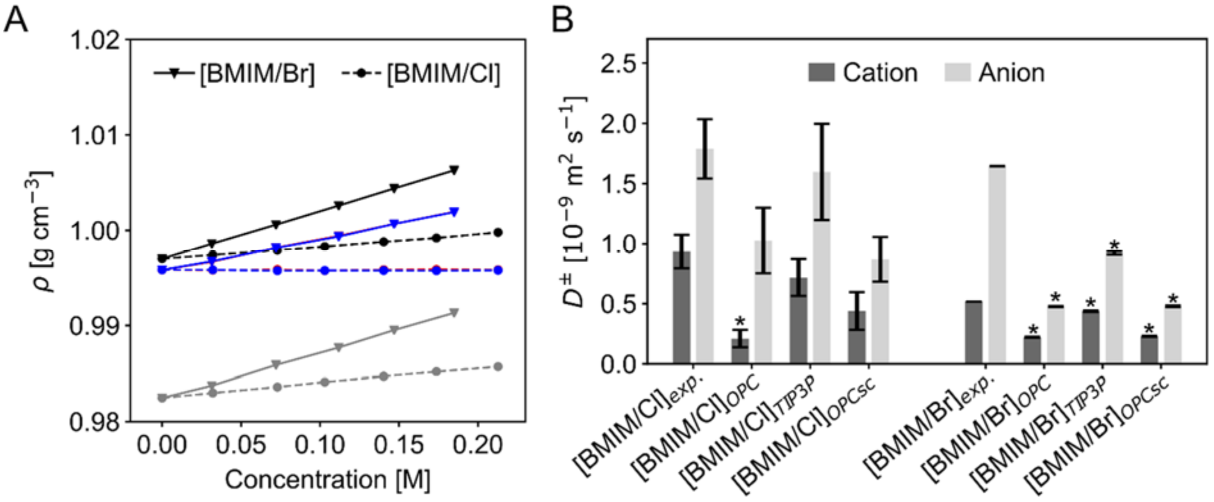


**Figure S1:** Influence of water model and partial charges on properties of IL. (A) Density-concentration relationship for systems of ▼ [BMIM/Br] and ● [BMIM/Cl] (exp. values from ref. [1]: black, OPC/1.0*e*: red (mostly covered by the blue OPC/0.9*e* line), OPC/0.9*e*: blue, TIP3P/1.0*e*: grey). (B) Self-diffusion coefficients for cations (black) and anions (grey) of [BMIM/Cl] and [BMIM/Br], respectively. Data is shown as mean ± standard error of the mean ($n = 5$). Experimental values are averaged over all available experimental sources of refs. [2-6]. OPC: OPC/1.0*e*, TIP3P: TIP3P/1.0*e*, OPCsc: OPC/0.9*e*. Significant differences as to experiment ($p \leq 0.05$, two-sided independent Student's *t*-test) are marked with an asterisk.

## 2.2 Time evolution of the spatial distribution of ions

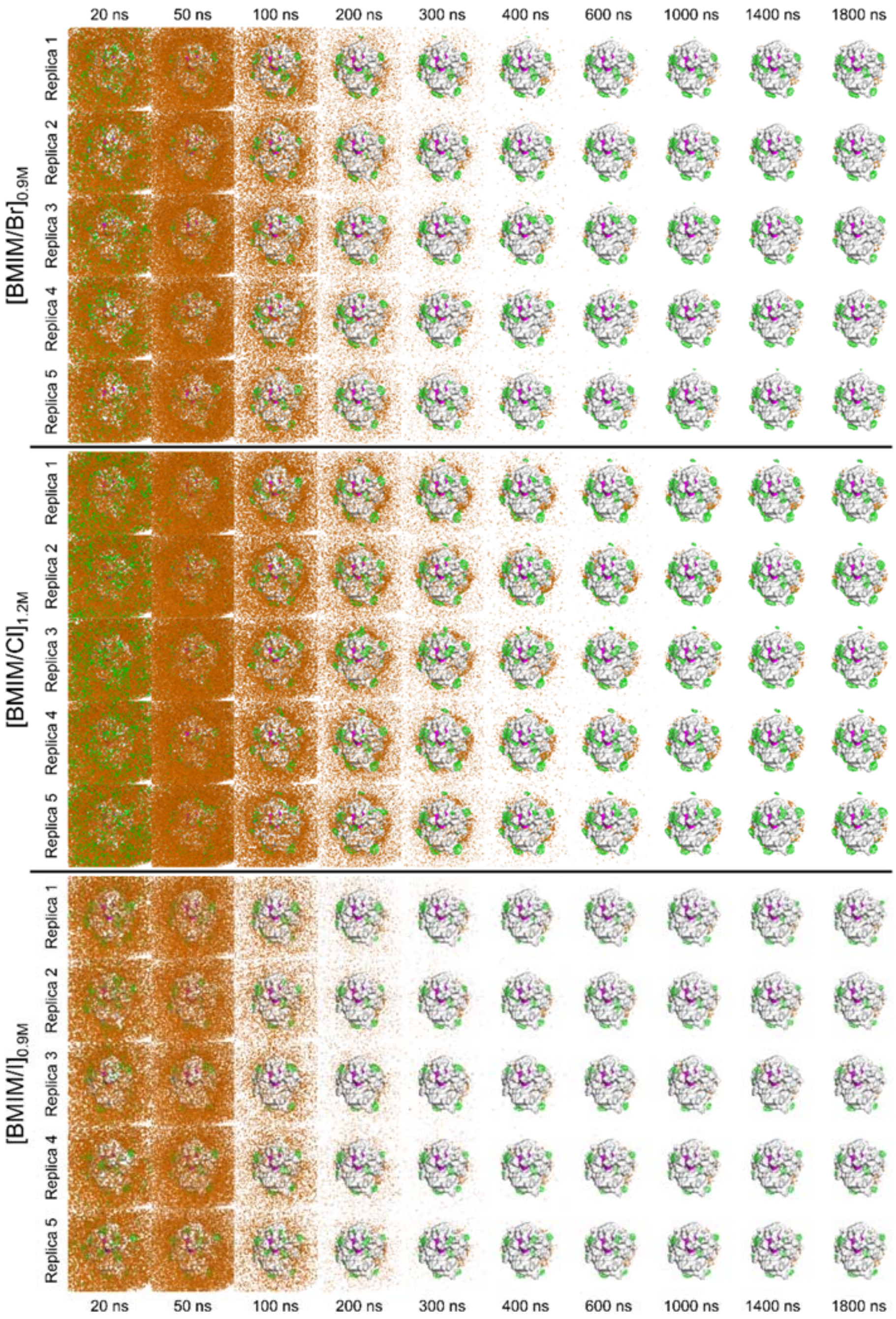


**Figure S2A:** Evolution of the spatial distribution of cation and anions at the *Bs*LipA surface. Density grids are shown for each replica of 0.9 M [BMIM/Br] (upper panel), 1.2 M [BMIM/Cl] (middle panel), and 0.6 M [BMIM/I] (lower panel) after 20 ns, 50 ns, 100 ns, 200 ns, 300 ns, 400 ns, 500 ns, 600 ns, 1000 ns, 1400 ns, and 1800 ns. All distributions were normalized according to the number of frames, which is equivalent to the cumulative simulation time. Areas with a high density of [$BMIM^+$] throughout MD simulations are shown as green meshes and areas for [$Br^-$], [$Cl^-$], or [$I^-$] ions as orange meshes, respectively. The purple patches show the partial surface area of the catalytic site residues. Densities of water are removed for clarity. To stay consistent with the main text and **Figure S4**, $\sigma$-values of 0.04 for [$BMIM^+$] and 0.0025 for [$Br^-$], [$Cl^-$], and [$I^-$] were used.

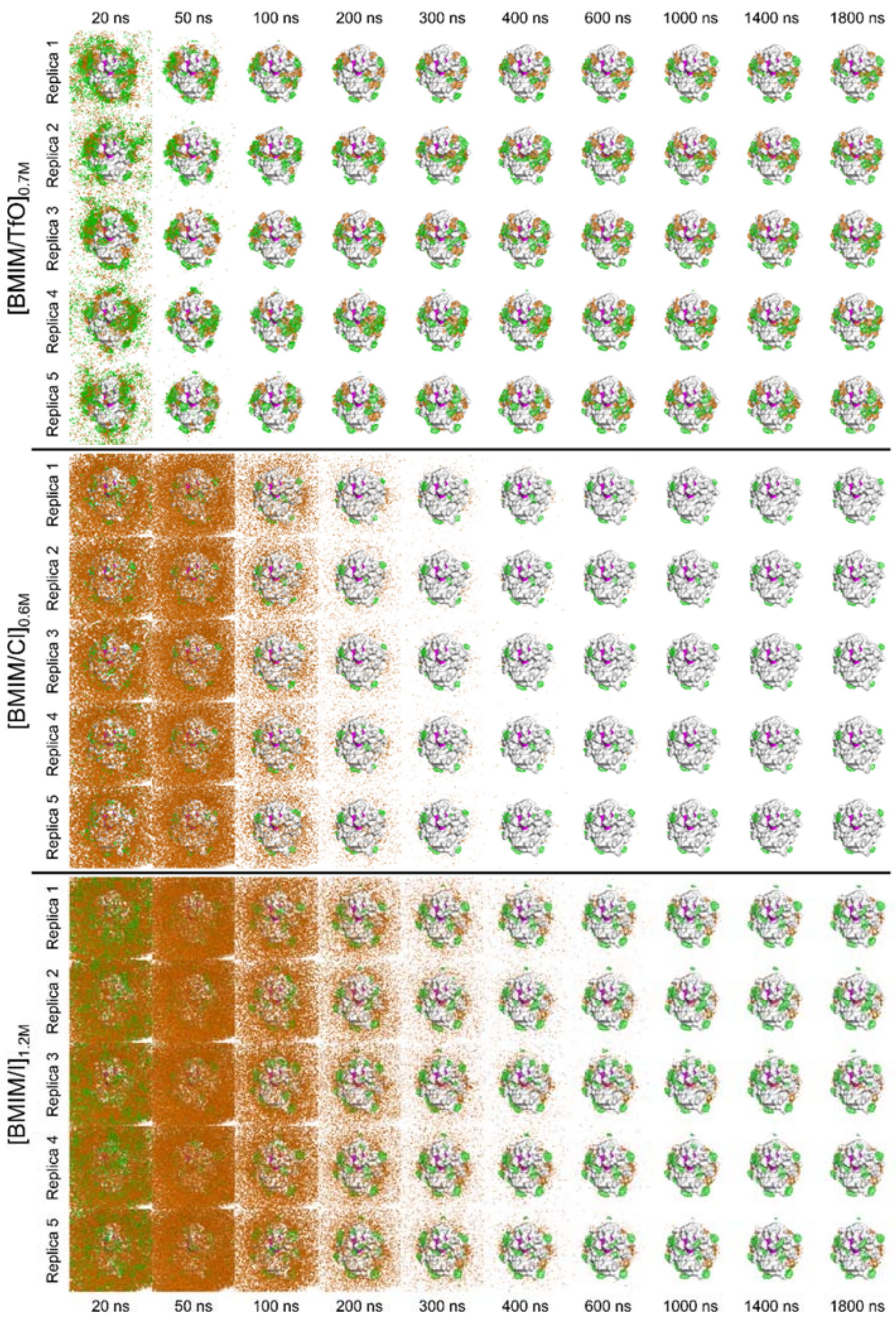


**Figure S2B:** Evolution of the spatial distribution of cation and anions at the *Bs*LipA surface. Density grids are shown for each replica of 0.7 M [BMIM/TfO] (upper panel), 0.6 M [BMIM/Cl] (middle panel), and 1.2 M [BMIM/I] (lower panel) after 20 ns, 50 ns, 100 ns, 200 ns, 300 ns, 400 ns, 500 ns, 600 ns, 1000 ns, 1400 ns, and 1800 ns. All distributions were normalized according to the number of frames, which is equivalent to the cumulative simulation time. Areas with a high density of [$BMIM^+$] throughout MD simulations are shown as green meshes and areas for [$TfO^-$], [$Cl^-$], or [$I^-$] ions as orange meshes, respectively. The purple patches show the partial surface area of the catalytic site residues. Densities of water are removed for clarity. To stay consistent with the main text and **Figure S4**, $\sigma$-values of 0.04 for [$BMIM^+$], 0.02 for [$TfO^-$], and 0.0025 for [$Cl^-$] and [$I^-$] were used.

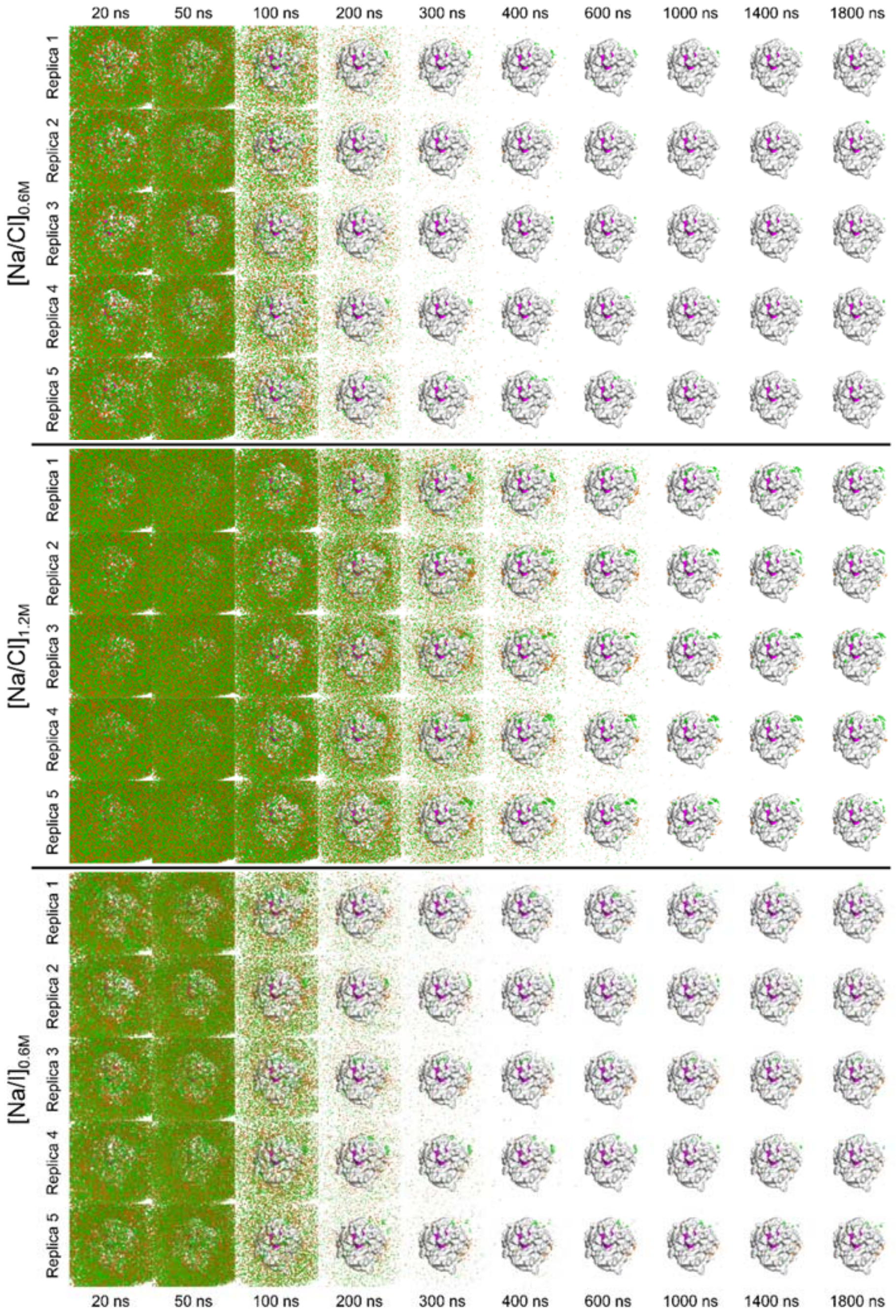


**Figure S2C:** Evolution of the spatial distribution of cation and anions at the *Bs*LipA surface. Density grids are shown for each replica of 0.6 M [Na/Cl] (upper panel), 1.2 M [Na/Cl] (middle panel), and 0.6 M [Na/I] (lower panel) after 20 ns, 50 ns, 100 ns, 200 ns, 300 ns, 400 ns, 500 ns, 600 ns, 1000 ns, 1400 ns, and 1800 ns. All distributions were normalized according to the number of frames, which is equivalent to the cumulative simulation time. Areas with a high density of [$Na^+$] throughout MD simulations are shown as green meshes and areas for [$Cl^-$] or [$I^-$] ions as orange meshes, respectively. The purple patches show the partial surface area of the catalytic site residues. Densities of water are removed for clarity. To stay consistent with the main text and **Figure S4**, $\sigma$-values of 0.0025 were used for all ions.

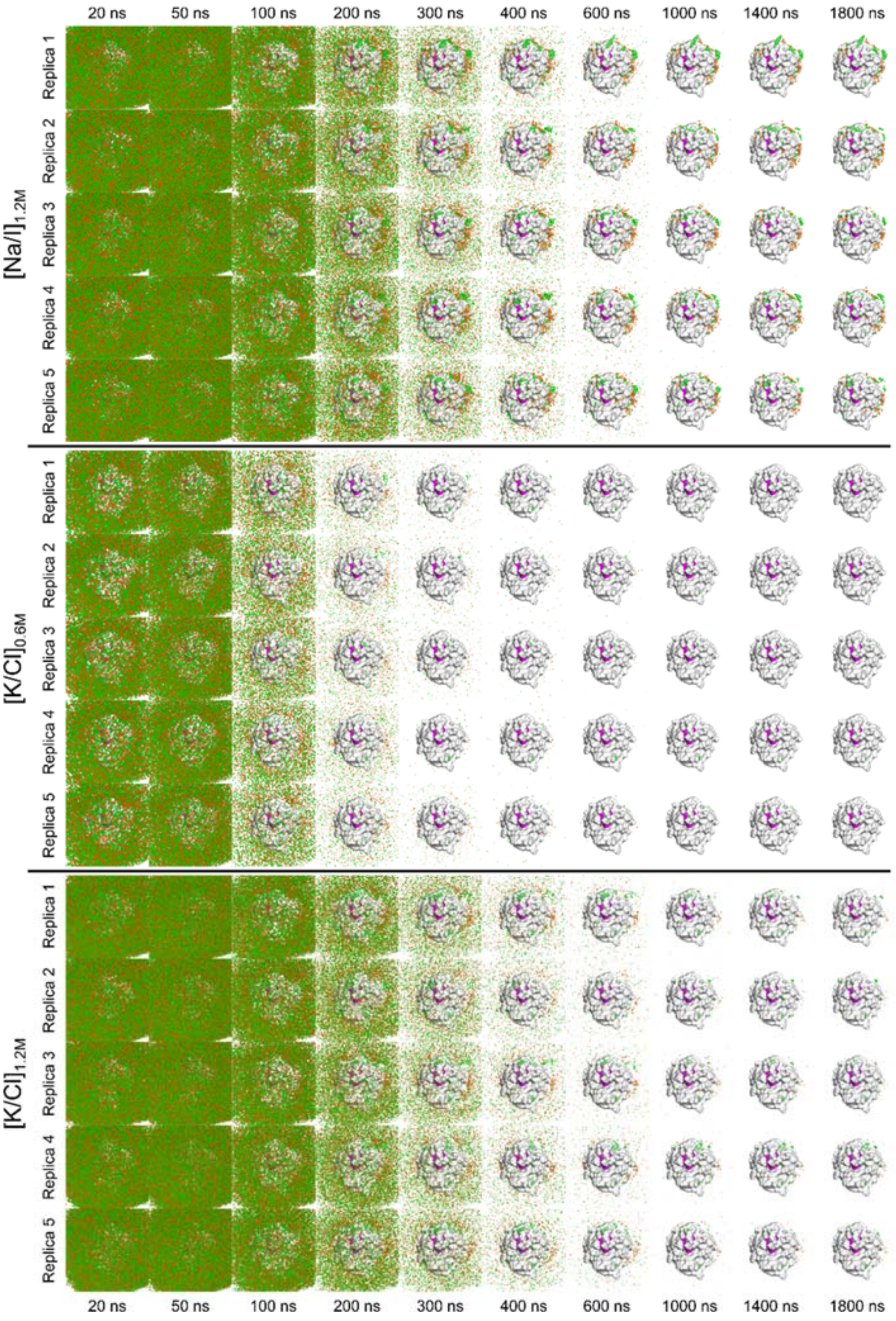


**Figure S2D:** Evolution of the spatial distribution of cation and anions at the *Bs*LipA surface. Density grids are shown for each replica of 1.2 M [Na/I] (upper panel), 0.6 M [K/Cl] (middle panel), and 1.2 M [K/Cl] (lower panel) after 20 ns, 50 ns, 100 ns, 200 ns, 300 ns, 400 ns, 500 ns, 600 ns, 1000 ns, 1400 ns, and 1800 ns. All distributions were normalized according to the number of frames, which is equivalent to the cumulative simulation time. Areas with a high density of [$Na^+$] or [$K^+$] throughout MD simulations are shown as green meshes and areas for [$Cl^-$] or [$I^-$] ions as orange meshes, respectively. The purple patches show the partial surface area of the catalytic site residues. Densities of water are removed for clarity. To stay consistent with the main text and **Figure S4**, $\sigma$-values of 0.0025 were used for all ions.

## 2.3 Dependency of the amount of surface waters on the amount of system waters

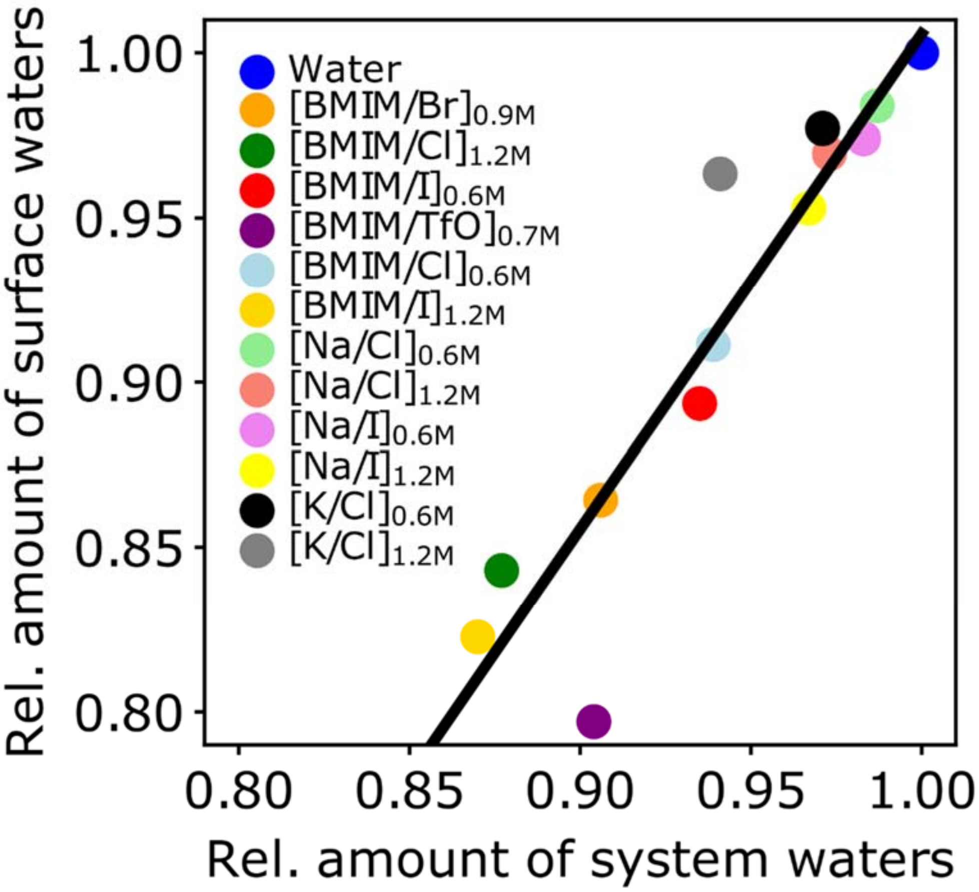


**Figure S3:** Dependency of the amount of water molecules on the protein surface on the amount of water molecules in the simulation box for all investigated solvents. The x-axis denotes the relative amount of waters in the simulation box compared to pure water. The y-axis denotes the relative amount of surface waters compared to pure water. Data points are shown as the mean ($n$ = 5).

## 2.4 Complete overview of spatial distribution for all solvent molecules

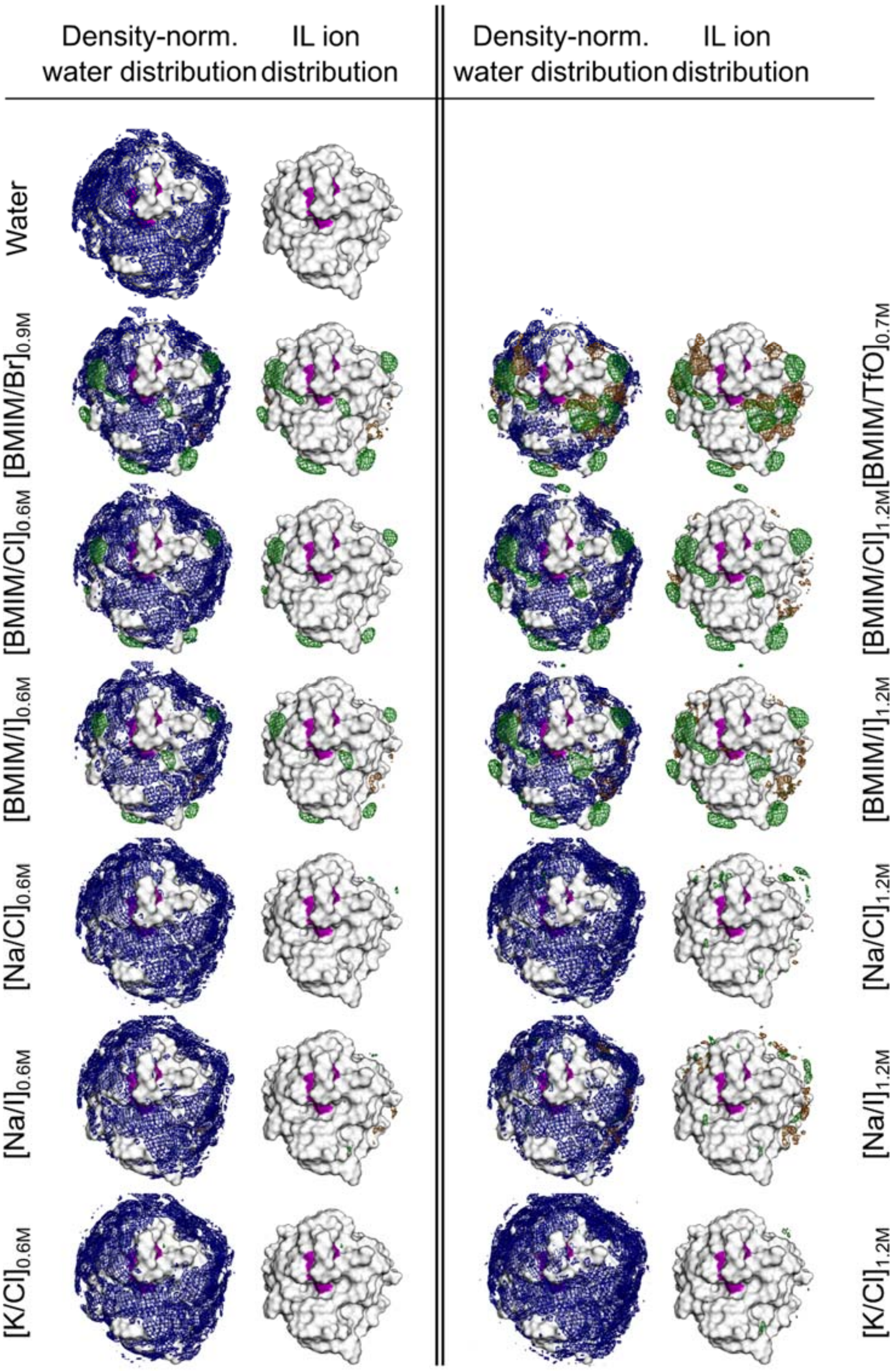


**Figure S4:** Spatial distribution of solvent molecules around the *Bs*LipA surface. Areas with a high density of water molecules throughout MD simulations are shown as blue meshes, areas for the respective cations as green meshes, and areas for anions as orange meshes. The purple patches show the partial surface area of the catalytic site residues. In the left panels of each column, the densities for water molecules were normalized to the number of water molecules in the simulation box; in the right panels, water densities were removed for clarity. All distributions were normalized according to the number of frames. $\sigma$-values defining the intensity cutoff of the represented data of 0.04 for [BMIM$^+$], 0.02 for [TfO$^-$], and 0.0025 for alkali metals/halides were used.

## 2.5 [TfO$^-$]-induced catalytic site-crowding by [BMIM$^+$] ions

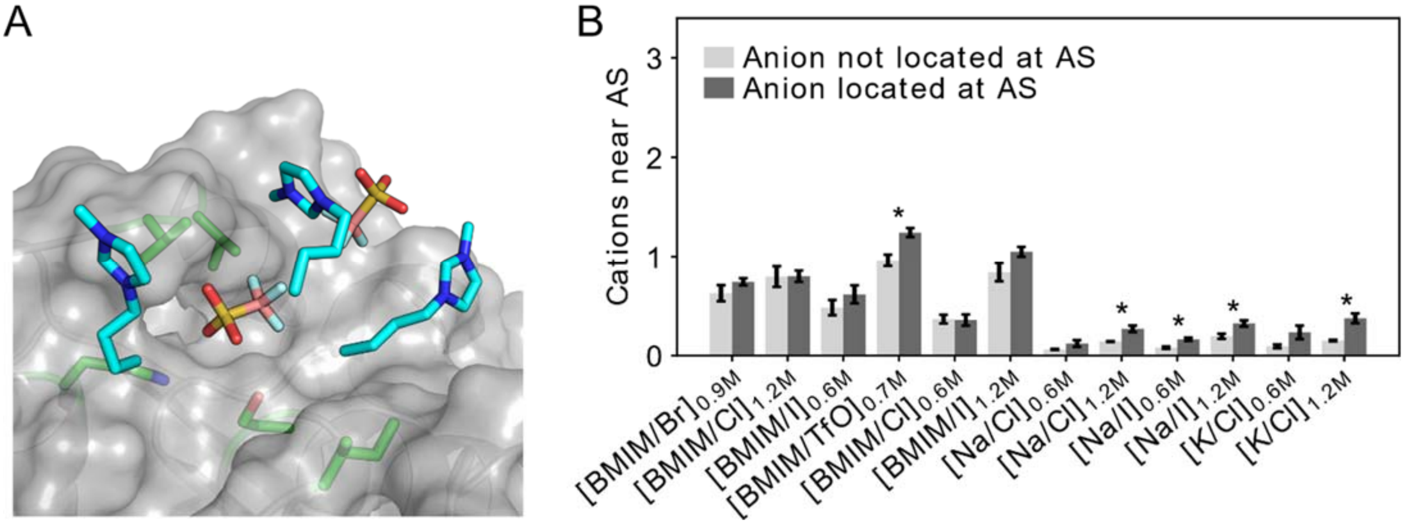


**Figure S5:** [TfO$^-$] anions may deteriorate substrate access to the catalytic site by inducing crowding of [BMIM$^+$] molecules. (A) Snapshot of the catalytic site region with [TfO$^-$] anion bound in the catalytic site cleft interacting with butyl moieties of multiple [BMIM$^+$] cations. Residues of *Bs*LipA involved in [TfO$^-$] binding are depicted as sticks with carbon atoms colored in green. Carbon atoms of [BMIM$^+$] and [TfO$^-$] are colored in cyan and orange, respectively. (B) The average number of cations within 10 Å of S77 when an anion is bound/not bound at the catalytic site (distance to S77Oγ lower than/greater than 5 Å, the average cut off over all anions). We observed a significant ($p \leq 0.05$, two-sided independent Student's *t*-test) increase in the average number of [BMIM$^+$] molecules when [TfO$^-$] is present at the catalytic site cleft. A similar increase in the number of cations when an anion is present in the catalytic site cleft was also observed for multiple salt solutions, but not for other [BMIM$^+$]-containing solvents. Data is shown as mean ± standard error of the mean. Significant differences are marked with an asterisk.

## 2.6 Radial distribution functions

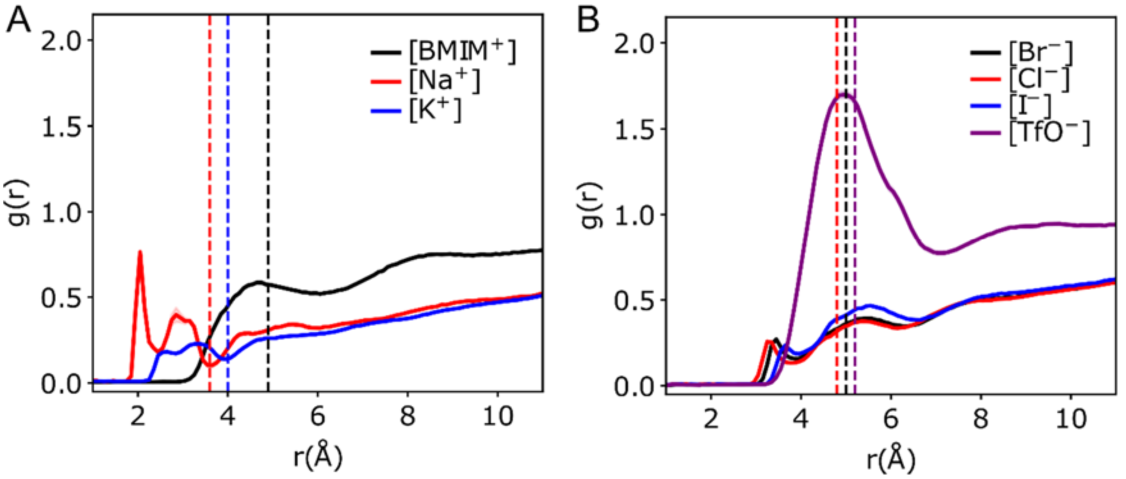


**Figure S6:** Radial distribution functions for IL and salt ions around *Bs*LipA residues. The derived cutoff distances used to define bound states in subsequent analyses are marked with a vertical dashed line. (A) Analysis of cation RDFs for systems of 0.6 M [BMIM/Cl] (black), 0.6 M [Na/Cl] (red), and 0.6 M [K/Cl] (blue). (B) Analysis of anion RDFs for systems of 0.9 M [BMIM/Br] (black), 1.2 M [BMIM/Cl] (red), 0.6 M [BMIM/I] (blue), and 0.7 M [BMIM/TfO] (purple). Data is shown as mean ± standard error of the mean ($n$ = 5).

## 2.7 Analysis of binding and unbinding events

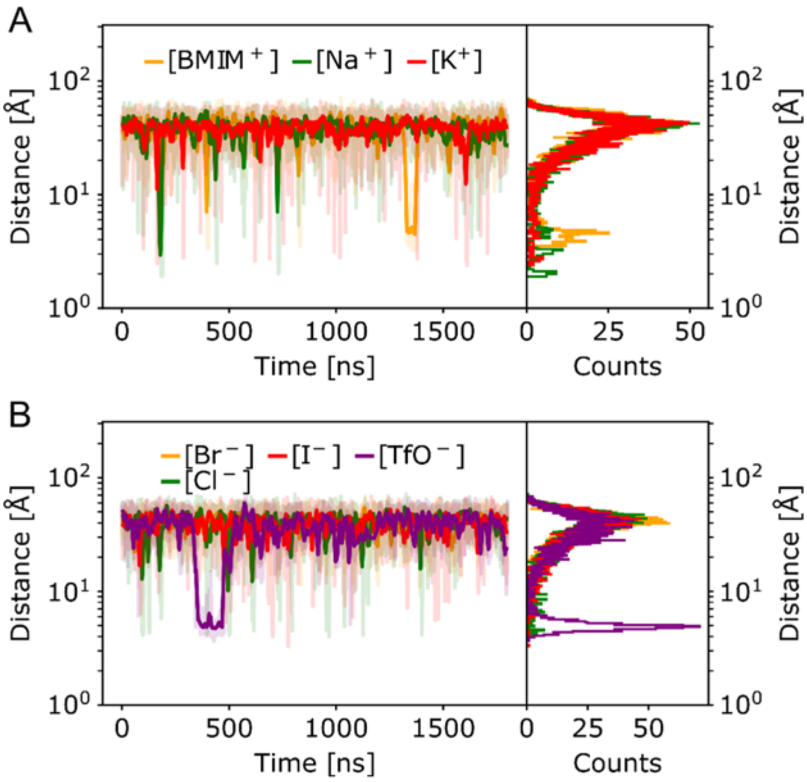


**Figure S7:** Distances from most occupied residues to highest occupying ions over the course of the trajectory. Solid lines in the time-resolved plot show the distance smoothed over ±50 frames and transparent lines show the raw data. (A) Distances of ions that showed the most frequent interactions to W42 for [$BMIM^+$] (yellow) or E65 for [$Na^+$] (green) and [$K^+$] (red), respectively. (B) Ion-residue distances for [$TfO^-$] (purple) to K23 or [$Br^-$] (yellow), [$Cl^-$] (green), and [$I^-$] (red) to R57.

## 2.8 Time evolution of selected binding free energies

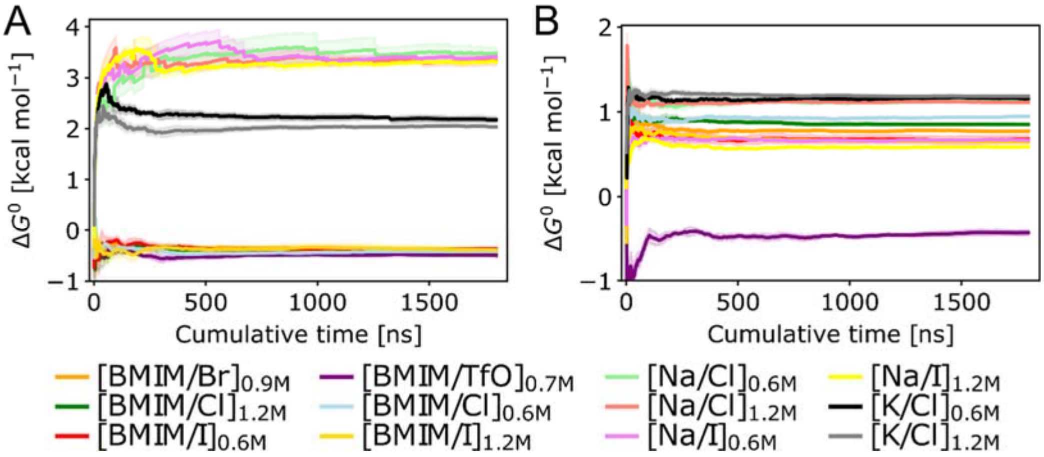


**Figure S8:** Evolution of binding free energies during the MD simulations. The y-axis denotes the average binding free energy over the five independent trajectories. The x-axis denotes the simulation time ($t$). Data is shown as mean ± standard error of mean ($n = 5$). (A) Absolute binding free energies of cation interactions with residue W42 for all systems. (B) Absolute binding free energies of anion interactions with residue K23 for all systems. All binding free energies converge ($\Delta(\Delta G^0_{\text{final}} - \Delta G^0(t)) \leq 0.5$ kcal mol$^{-1}$) within the simulation time: Interactions with negative binding free energies (at 1 M standard state) converge within the first 100 ns (e.g., $\Delta G^0_{[\text{BMIM}+],\text{W42}}$ or $\Delta G^0_{[\text{TfO}-],\text{K23}}$); interactions with positive binding free energies (at 1 M standard state) require up to 600 ns to converge (e.g., $\Delta G^0_{[\text{Na}+],\text{W42}}$).

## 2.9 RMSF of *Bs*LipA backbone atoms for selected solvents

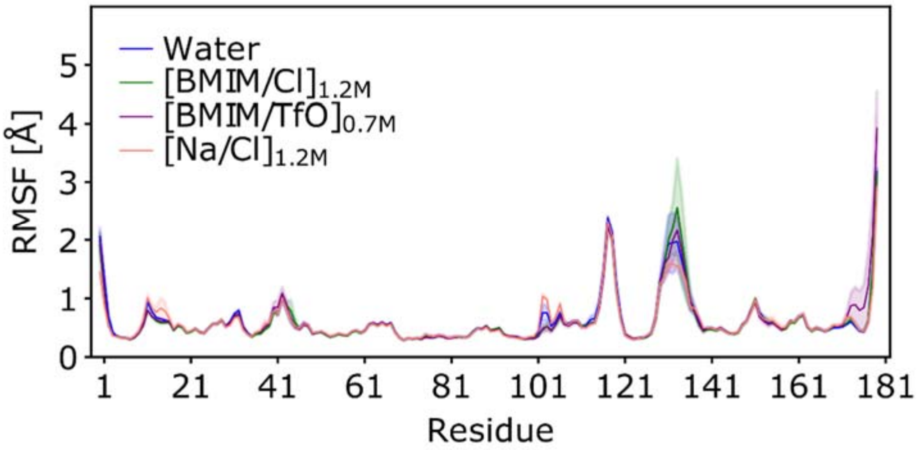


**Figure S9:** *Bs*LipA displays no changes in atomic mobility of protein backbone residues upon incubation in ionic solutions. RMSF analyses of backbone atoms for *Bs*LipA in ionic solutions showed no significant change compared to water (blue). Values are exemplarily shown for 1.2 M [BMIM/Cl] (green), 0.7 M [BMIM/TfO] (purple), and 1.2 M [Na/Cl] (salmon). Data is shown as mean ± standard error of the mean ($n$ = 5).

## 2.10 Hydrophobic surface area

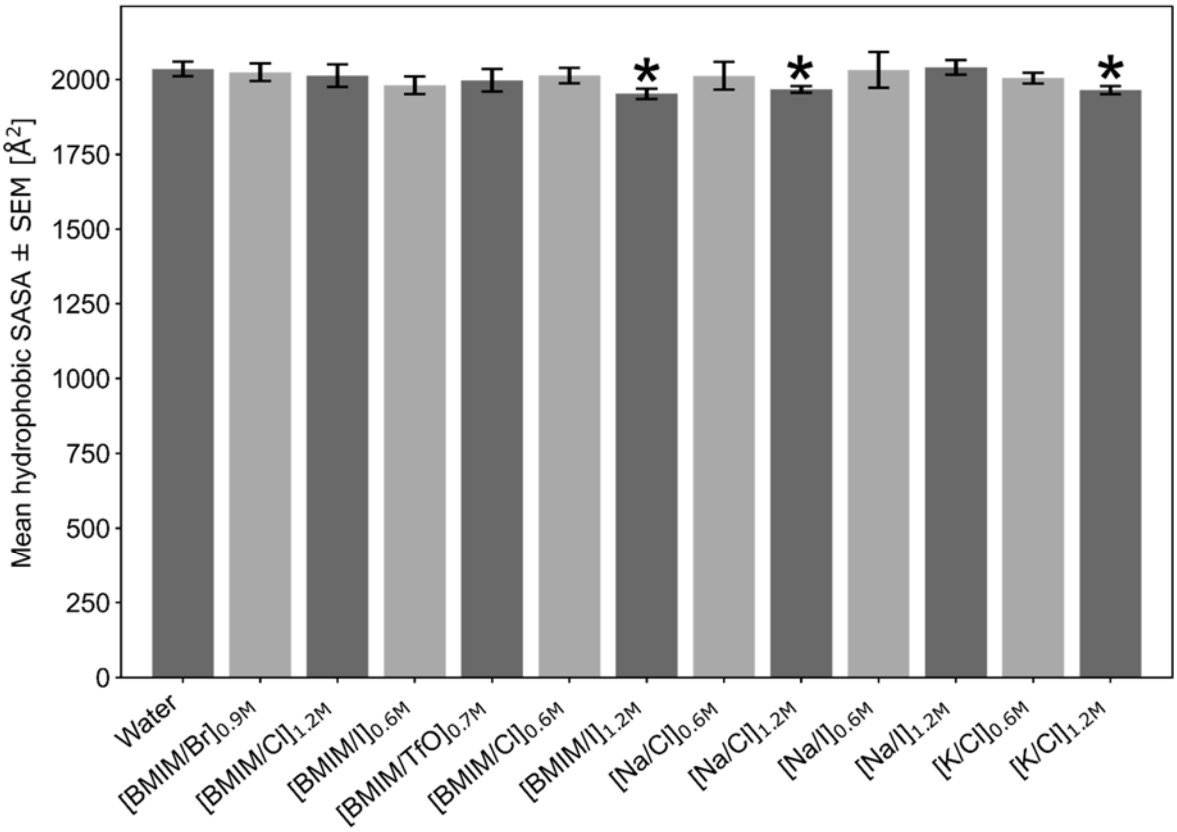


**Figure S10:** Average hydrophobic solvent-accessible surface area of *Bs*LipA. The y-axis denotes the average hydrophobic SASA over the five independent MD trajectories for each solvent. Data is shown as mean ± standard error of the mean ($n = 5$). Significant ($p \leq 0.05$, two-sided independent Student's *t*-test) changes with respect to water are indicated with an asterisk.

## 2.11 Global protein structure descriptors

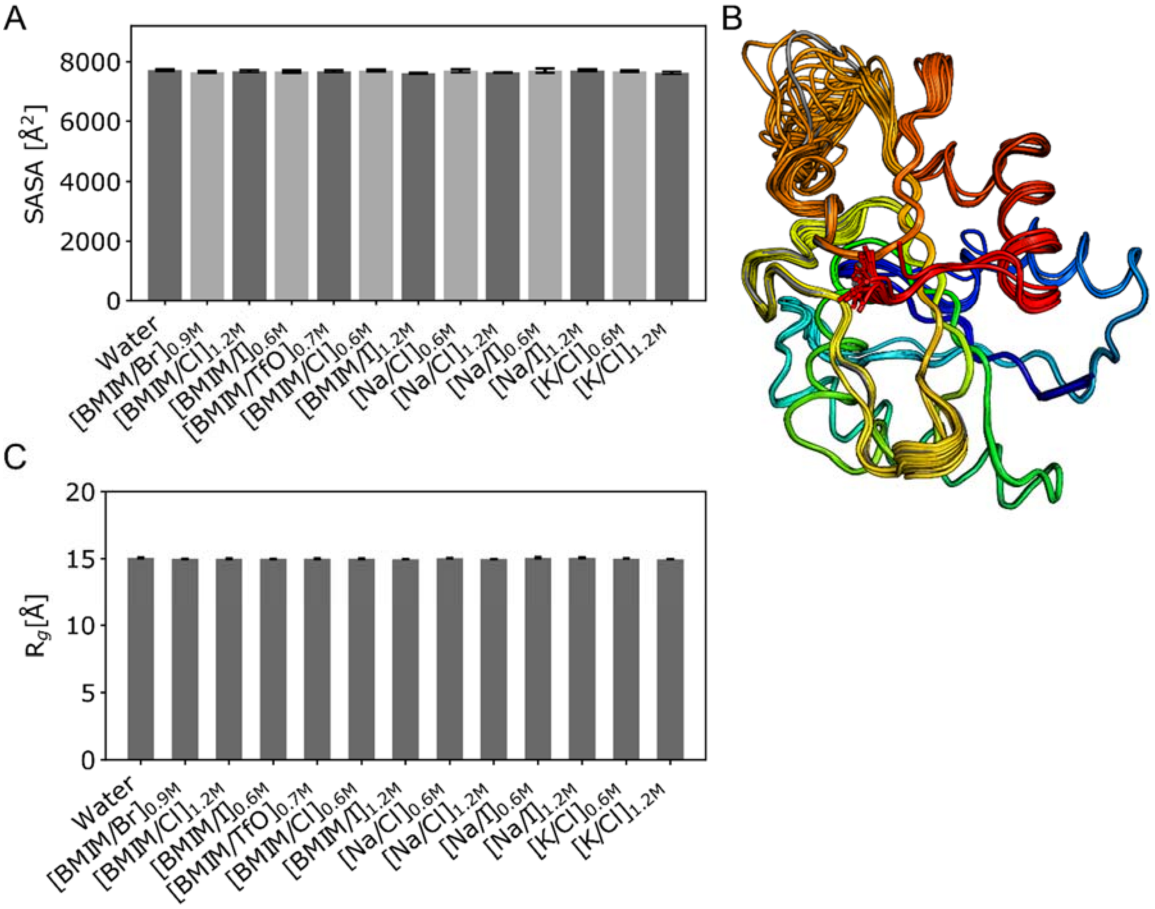


**Figure S11:** No descriptor of global protein structure reveals major changes in *Bs*LipA structure upon incubation in ionic solutions. (A) Solvent-accessible surface area of *Bs*LipA. The y-axis denotes the average SASA over the five independent trajectories for each solvent. Data is shown as mean ± standard error of the mean ($n$ = 5). There is no significant change with respect to water. (B) Average structure of each replica for all solvents (rainbow-colored from N-terminus (blue) to C-terminus (red)) and water (grey structures). The average structures largely superimpose perfectly. Only a few residues, mostly in loops, show more considerable mobility. (C) The radius of gyration of *Bs*LipA. Data is shown as mean ± standard error of mean ($n$ = 5). There is no significant change with respect to water.

## 2.12 CNA difference neighbor stability maps

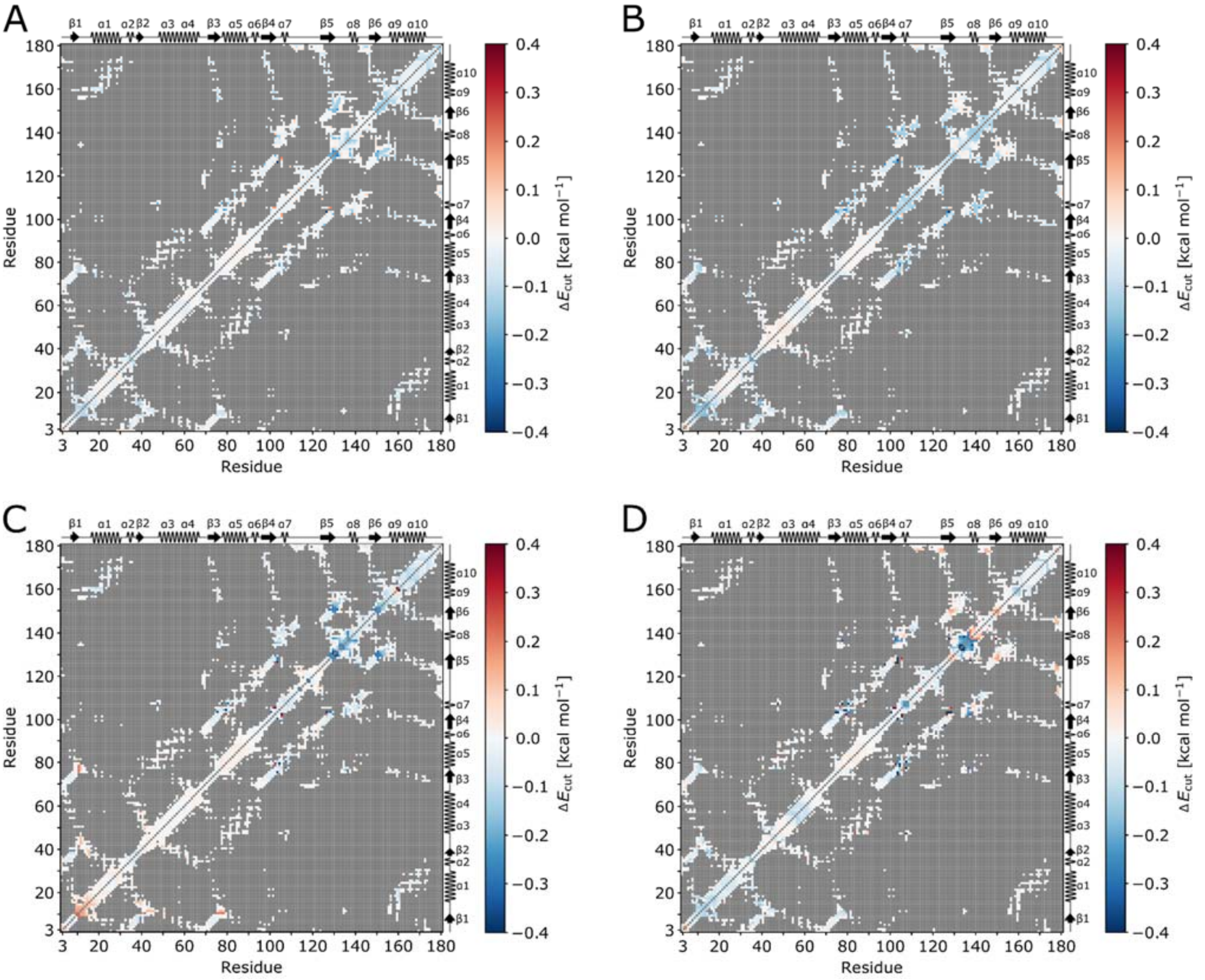


**Figure S12:** Incubation of *Bs*LipA in aIL induce changes in the local structural stability. (A-D) $\Delta rc_{ij,neighbor}$ of 0.9 M [BMIM/Br], 1.2 M [BMIM/Cl], 0.6 M [BMIM/I], 0.7 M [BMIM/TfO] with respect to water, highlighting changes in the protein stability upon incubation in aIL. $\Delta E_{\text{cut}}$ describes the difference in energy when a rigid contact between two residues $R_{i,j}$ is lost during the thermal unfolding simulation with red (blue) colors denoting lower (higher) energies, thus indicating that a contact is less (more) stabilized in aIL than in water. Only contacts of residues that are at most 5 Å apart are considered; contacts with higher distances are shown in grey. Data is shown as mean over all replica ($n$ = 5).

## 2.13 Dependency of perturbed interactions on the concentration.

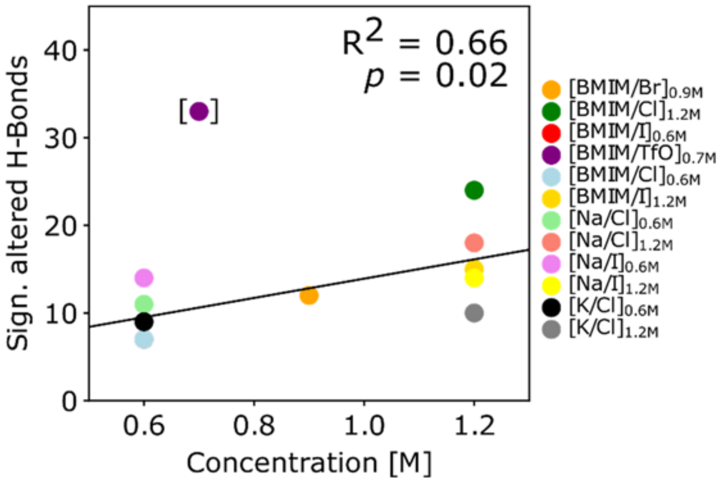


**Figure S13:** Significantly altered hydrogen bonds with respect to the concentration. 0.7 M [BMIM/TfO] was excluded from the regression analysis and is shown in brackets. The x-axis denotes the concenctration of the solvent. The y-axis denotes the average number of significantly altered interactions compared to pure water. Data is shown as mean ($n$ = 5).

## 2.14 Structural reorganization of the catalytic site S77

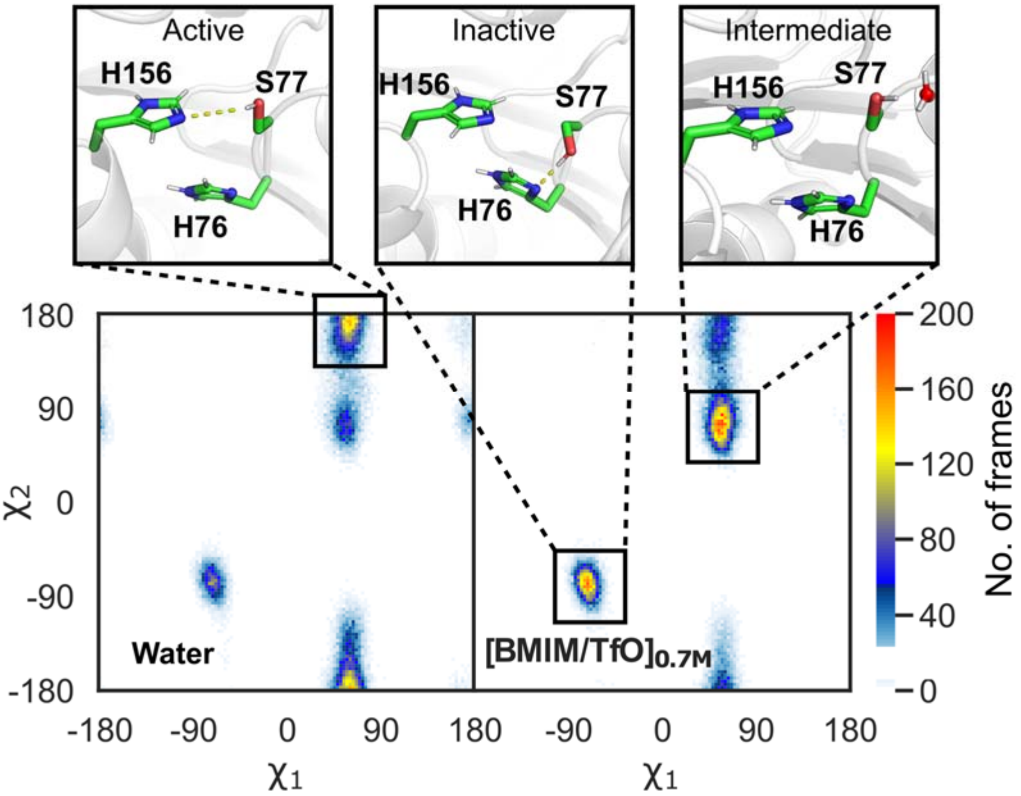


**Figure S14:** Ionic liquids disrupt the catalytic site integrity. Conformational space adopted by S77 in water and 0.7 M [BMIM/TfO]. The x-axis denotes the dihedral angle $\chi_1$ defined by atoms N, Cα, Cβ, Oγ, the y-axis the dihedral angle $\chi_2$ defined by atoms Cα, Cβ, Oγ, Hγ. Representative structures for active (left panel), intermediate (middle panel), and inactive (right panel) S77 conformations observed throughout the trajectories are shown. Incubation in ionic solutions led to a redistribution of the S77 conformations and an increased likelihood of the inactive conformation.

## 2.15 Structure of the IL ions

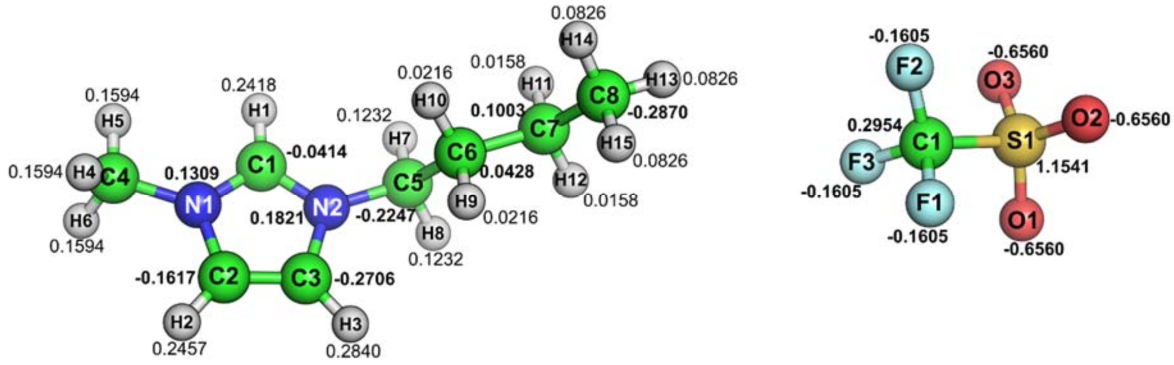


**Figure S15:** QM-derived structures and partial charges of [BMIM$^+$] and [TfO$^-$] ions used in all MD simulations. The initial structures were calculated using Gaussian 16 [7] at the HF/6-31G* level of theory [8]. Partial charges were derived following the RESP procedure [9]. For MD simulations of [BMIM/Br] and [BMIM/Cl] with scaled charges, all partial charges of [BMIM$^+$] were scaled by a factor of 0.9.

## 2.16 Conformational space analysis of S77 for all solvents

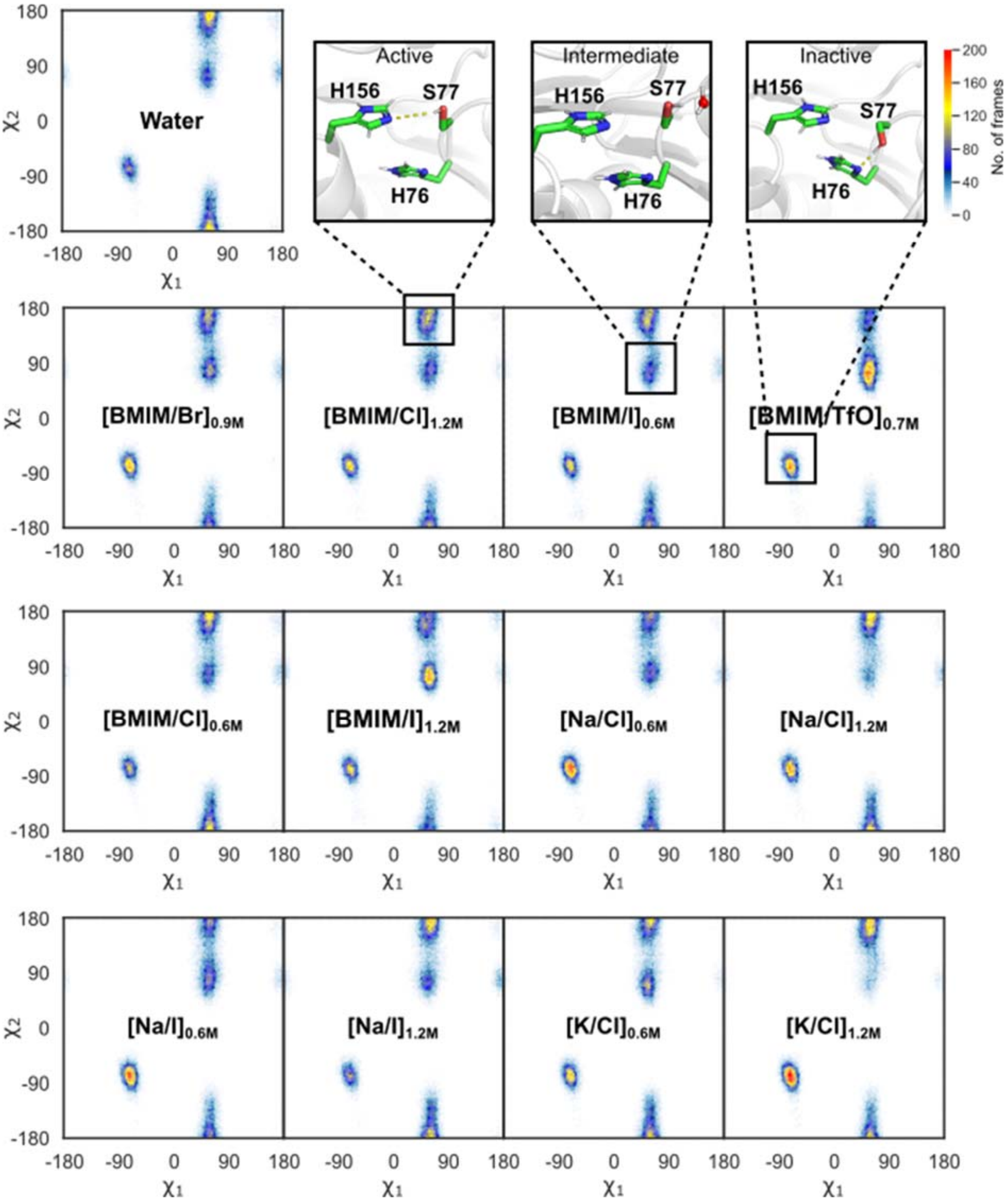


**Figure S16:** Conformational space adopted by S77 for all solvents. The x-axis denotes the dihedral angle χ1 defined by atoms N, Cα, Cβ, Oγ, the y-axis the dihedral angle χ2 defined by atoms Cα, Cβ, Oγ, Hγ. Representative structures for active (left panel), intermediate (middle panel), and inactive (right panel) S77 conformations observed in the MD trajectories are shown. Incubation in aIL led to a redistribution of the S77 conformations and an increased likelihood of the inactive conformation.

## 2.17 Probabilities of the catalytic site conformations for all solvents

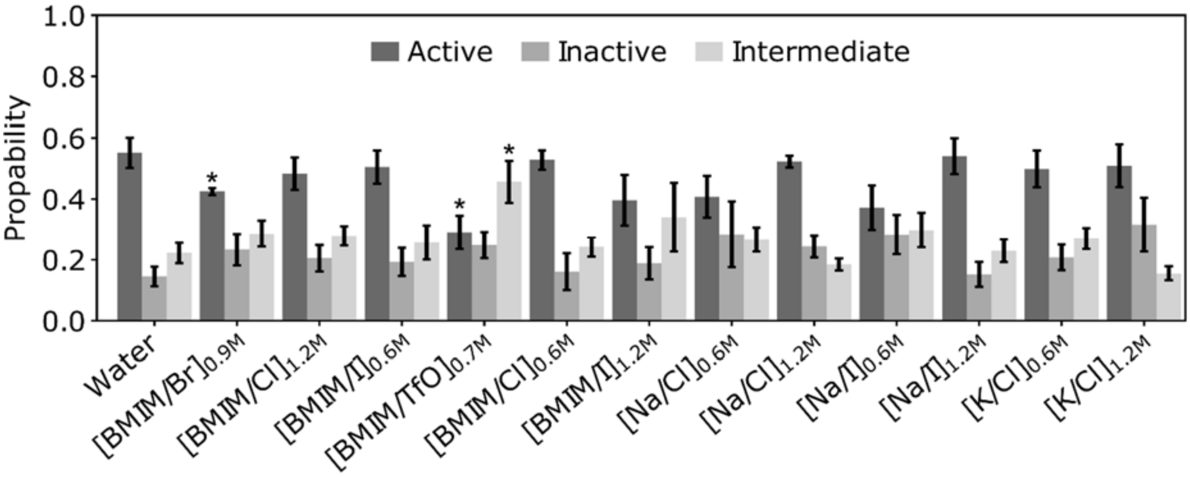


**Figure S17:** Probability of S77 conformations. Probabilities for the catalytically active conformation (dark grey), the intermediate conformation (middle grey), and the inactive conformation (light grey) are shown. Incubation in aIL led to a significant redistribution of the adapted conformations in 0.9 M [BMIM/Br] and 0.7 M [BMIM/TfO]. Data is shown as mean ± standard error of the mean ($n = 5$). Significant differences ($p \leq 0.05$, two-sided independent Student's $t$-test) with respect to the values from the water simulation are marked with an asterisk.

## 2.18 Time evolution of hydrogen bond frequencies in the active site

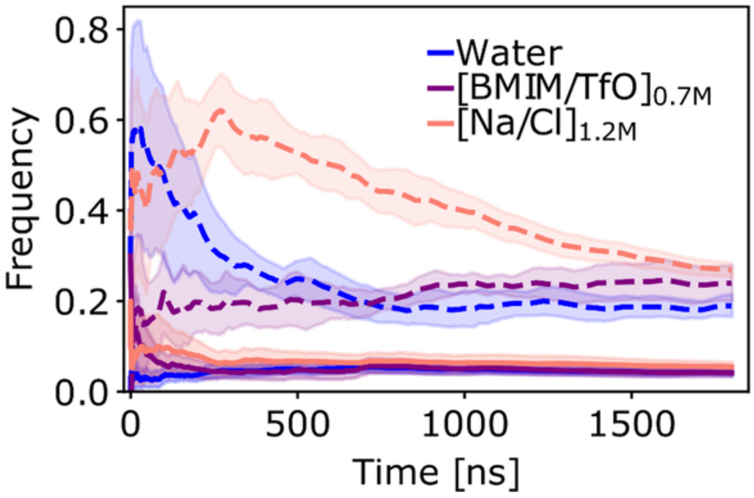


**Figure S18**: Evolution of the hydrogen bond frequencies of the active (solid line) and inactive (dashed line) state during the MD simulations for water (blue), 0.7 M [BMIM/TfO] (purple), and 1.2 M [Na/Cl] (orange). Data is shown as mean ± standard error of mean ($n = 5$). ~200 ns and ~1600 ns of simulation time were required to obtain converged occurrence frequencies.

# 3 Supplementary Results

## 3.1 Text S1: Dynamic properties of aIL can be reasonably well reproduced using current force field and water models

The mean system densities considering all atoms for [BMIM/Br] using the OPC water model with unscaled ion charges followed a linear trend from 0.996 g $cm^{-3}$ at 0 M to 1.006 g $cm^{-3}$ at the highest concentration of 0.1849 M (**Figure S1A**). For [BMIM/Cl], the density is 0.996 g $cm^{-3}$ across all concentrations. Scaling the charges to ±0.9*e* as suggested [10] yielded identical results, thus, data points from the OPC water model with unscaled ion charges (red line) are almost completely covered by data points of the water model with scaled ion charges (blue line). With the TIP3P water model, the [BMIM/Br] system showed densities from 0.982 g $cm^{-3}$ at 0 M to 0.990 g $cm^{-3}$ at the highest concentration, while the [BMIM/Cl] system showed a density of 0.982 g $cm^{-3}$ at 0 M up to 0.985 g $cm^{-3}$ at the highest concentration. The SEM was < 0.001 g $cm^{-3}$ for all systems. In general, the slopes of the mean system densities with all investigated water models are in very good accordance with experimental results from ref. [11] (**Figure S1A**), suggesting that equilibrium properties are well reproduced by all shown water model and ion partial charge combinations. This finding agrees with previous results on pure IL [12]. The absolute values are underestimated by < 0.5% (< 0.005 g $cm^{-3}$) for the OPC water model with both scaled and unscaled ion partial charges and by ~ 1.5% (0.015 g $cm^{-3}$) for the TIP3P water model with unscaled ion charges (**Figure S1A**). Interestingly, underestimation of densities of IL has previously been shown for the GAFF force field and an IL-specific refined OPLS-AA force field for pure IL and, thus, seems to carry over to aIL albeit with a reduced effect [12]. Since multiple studies [13-16] revealed that transport properties of pure IL are underestimated in MD simulations when using non-polarizable force fields, we additionally computed the self-diffusion coefficients for both cations and anions in aIL (**Figure S1B**). To do so, we set up systems of [BMIM/Cl] at infinite dilution as well as of [BMIM/Br] at a concentration of 1.309 M with the same water model and partial charge combinations used in the density calculations (see **section 5.1** of the main text). For [BMIM/Cl], unscaled (scaled) partial charges and the OPC water model led to diffusion coefficients of $0.17 \cdot 10^{-9}$ ($0.39 \cdot 10^{-9}$) $m^2 s^{-1}$ for [$BMIM^+$] and $1.00 \cdot 10^{-9}$ ($0.72 \cdot 10^{-9}$) $m^2 s^{-1}$ for [$Cl^-$]. The TIP3P water model resulted in higher values with diffusion coefficients of $0.7 \cdot 10^{-9}$ $m^2 s^{-1}$ for [$BMIM^+$] and $1.37 \cdot 10^{-9}$ $m^2 s^{-1}$ for [$Cl^-$], in agreement with the lower viscosity of this water model [17]. For [BMIM/Br], we observed self-diffusion coefficients of $0.22 \cdot 10^{-9}$ $m^2 s^{-1}$ for [$BMIM^+$] and $0.48 \cdot 10^{-9}$ $m^2 s^{-1}$ for [$Br^-$] with the OPC water model and unscaled ion charges; scaling the charges did not yield significant ($p \leq 0.05$, two-sided independent Student's *t*-test) differences. The self-diffusion coefficients obtained with unscaled ion charges and the TIP3P water model were $0.44 \cdot 10^{-9}$ $m^2 s^{-1}$ and $0.92 \cdot 10^{-9}$ $m^2 s^{-1}$ for [$BMIM^+$] and [$Br^-$]. Experimental studies reported self-diffusion coefficients of [BMIM/Br] and [BMIM/Cl] at infinite dilution or very low concentrations in the range of 0.662-$1.324 \cdot 10^{-9}$ $m^2 s^{-1}$ for [$BMIM^+$], $1.644 \cdot 10^{-9}$ $m^2 s^{-1}$ for [$Br^-$] and $1.542 \cdot 10^{-9}$ $m^2 s^{-1}$ for [$Cl^-$], respectively [2,3,5,6] (**Figure S1B**).

All shown water model and ion partial charge combinations of aIL reproduce the thermodynamic properties reasonably well and show transport properties in the same order of magnitude as experimental values. Self-diffusion constants of [$BMIM^+$], [$Br^-$], and [$Cl^-$] tended to be underestimated by simulations with the OPC water model, whereas the TIP3P water model led to results closer to the experiment (**Figure S1B**). As the TIP3P water model overestimates the diffusion coefficient of pure water by a factor of roughly 2.4 [17], this also affects the dynamics of solutes in aqueous systems. Thus, more accurate transport properties potentially come at the cost of a markedly lowered reproducibility of multiple other bulk properties of water and aqueous systems when compared to the OPC water model, including the static dielectric constant, the density, and the heat of vaporization [17]. Further, the underestimation of the diffusion coefficients only affects kinetic properties of the system and not thermodynamic properties investigated in this study. Hence, for our aIL, we

decided to use the OPC water model due to the significantly improved results for multiple other bulk properties of water.

Interestingly, our results show that the commonly used approach of scaling the partial charges of the IL ions to account for charge transfer effects [15,18] did not lead to improved results for our aIL, neither for densities nor for self-diffusion coefficients. This result indicates that the charge transfer effect observed for pure IL affects simulations of aIL much less.

## 3.2 Text S2: Incubation in aIL potentially inactivates the enzyme by inducing a shift from the active to the inactive state of the catalytic site

To identify if the conformational changes induced by PP2 influences the activity of *Bs*LipA, we investigated the structural integrity of S77 in terms of dihedral angles $\chi_1$ (defined by the S77 atoms N, Cα, Cβ, Oγ) and $\chi_2$ (defined by the S77 atoms Cα, Cβ, Oγ, Hγ). $\chi_1$ describes the orientation of the hydroxyl oxygen, and $\chi_2$ the orientation of the hydroxyl hydrogen.

The catalytic site of *Bs*LipA contains the catalytic triad S77, H156, and D133. In the crystal structure, a hydrogen bond network is formed by a hydrogen bond between the hydrogen of Oγ of S77 and the deprotonated Nε of H156. Additionally, a second, alternative conformation of the catalytic triad of *Bs*LipA has been described [19], in which S77 does not form an H-bond with H156, but instead interacts with the adjacent H76 in a flipped conformation. This conformation was suggested to be an inactive state, related to the release of the reaction product [19]. These states were also visible in snapshots in ref. [20], but were not discussed in terms of their effect on catalytic site integrity or *Bs*LipA activity.

Three main states were found for all solvents with ($\chi_1$/$\chi_2$)-combinations of I) (75°/170°), II) (75°/80°), and III) (-80°/-80°) (**Figure S14; Figure S16**). State I) relates to the active conformation, as observed in the crystal structures of *Bs*LipA and state III) to the alternative conformation likely leading to inactive *Bs*LipA. State II) is an intermediate conformation where S77 interacts with solvent molecules. Incubation of *Bs*LipA in aIL induced a redistribution of the state populations compared to water (**Figure S17**). Particularly [BMIM/TfO] led to a decrease of the active conformation from 56% in water to 29% ($p < 0.01$, two-sided independent Student's *t*-test). By contrast, none of the salt solutions led to a significant reduction in the population of the active conformation. The reduction was accompanied by a significant increase in the intermediate conformation from 22% in water to 46% ($p < 0.02$, two-sided independent Student's *t*-test) in [BMIM/TfO], with no other solvent showing a similar increase of the intermediate conformation. As to the inactive conformation, compared to a population of 15% in water, [BMIM/TfO] induces an increase to 25% ($p < 0.09$, two-sided independent Student's *t*-test). Note that we did not observe significant correlations between the presence of [$BMIM^+$] or [$TfO^-$] ions at the catalytic site and a flip from the active conformation to the inactive conformation ($R^2 < 0.14$, $p = 0.80$; data not shown). Notably, the interactions needed at least 200 ns or 700 ns to converge (**Figure S18**), therefore making it unlikely that the simulation times of previous studies [20] were sufficient to reach convergence.

Taken together, our results suggest that incubation in aIL reduces the activity of *Bs*LipA by a shift of the active-to-inactive state equilibrium of the catalytic triad residue S77. As we found no evidence of the shift being caused by direct interactions of IL ions with the catalytic site, these results suggest that PP represent an indirect molecular mechanism of aIL induced protein inactivation.

## 3.3 Text S3: Investigation of a unique binding site in *Bs*LipA reveals an ion-specific cooperative binding effects that may impact enzyme activity through enhanced prevention of substrate access

In response to the unique binding site of [BMIM$^+$] molecules in 0.7 M [BMIM/TfO] depicted in **Figure 2** of the main text, we investigated the occupancy of IL ions at the catalytic site cleft.

We computed the distance of each ion to S77Oγ and determined how many ions were closer to the active site cleft than 10 Å; S77 is roughly located in the center of the catalytic site cleft. Here, we followed the procedure described in **section 5.6** of the main text and used the N2 or C1 atoms to describe the center of mass of [BMIM$^+$] or [TfO$^-$], respectively, and the ion center for alkali and halide ions. For [BMIM$^+$]-containing solvents, a close-by [TfO$^-$] led to a significant ($p < 0.01$, two-sided independent Student's *t*-test) increase of the average amount of [BMIM$^+$] ions close to the active site (distance from COM to S77Oγ ≤ 10 Å) (**Figure S5A**). We mainly observed hydrophobic interactions of the butyl moiety of [BMIM$^+$] with the trifluoromethyl group of [TfO$^-$]. On average, 0.96 [BMIM$^+$] molecules per frame were located within 10 Å of the catalytic site when no anion was present, and 1.24 molecules when [TfO$^-$] was located at the catalytic site. For other combinations of [BMIM$^+$] with anions such as [Cl$^-$] and [I$^-$], there is no significant increase in the average amount of [BMIM$^+$] close to the active site (**Figure S5B**). Interestingly, some of the solvents containing only salt ions also showed a significantly increased number of cations in the proximity of the catalytic site when anions were located at the catalytic site, however, on a lower scale. For example, as to 1.2 M [K/Cl], the presence of [Cl$^-$] at the catalytic site led to a significant increase ($p < 0.01$, two-sided independent Student's *t*-test) of [K$^+$] cations close to the catalytic site from 0.15 cations to 0.37 cations.

Taken together, these results indicate that certain aIL lead to an increased crowding of ions at the catalytic site, likely due to favorable interactions between the ions. This effect may inhibit enzyme activity by a competitive inhibition mechanism, preventing substrate access to the catalytic site or, more indirectly, by introducing electrostatic effects at the catalytic site. Inhibition due to blocked substrate access through crowding of [BMIM$^+$] at the entrance to the catalytic site of *Bs*LipA has also been suggested by Zhao *et al.* investigating the I12F and I12R variants of *Bs*LipA [20] in 0.7 M [BMIM/TfO] and, thus, may be especially prominent in solvents with anions that can bridge interactions between the protein and the cation.

# 4 Supplementary References


**1.** Frauenkron-Machedjou VJ, Fulton A, Zhu L, Anker C, Bocola M, *et al.* (2015) Towards understanding directed evolution: More than half of all amino acid positions contribute to ionic liquid resistance of Bacillus subtilis lipase A. ChemBioChem*,* 16(6), 937-945. https://doi.org/10.1002/cbic.201402682

**2.** Bešter-Rogač M, Fedotova MV, Kruchinin E, & Klähn M (2016) Mobility and association of ions in aqueous solutions: the case of imidazolium based ionic liquids [10.1039/C6CP05010G]. Physical Chemistry Chemical Physics*,* 18(41), 28594-28605. https://doi.org/10.1039/C6CP05010G

**3.** Nakakoshi M, Ishihara S, Utsumi H, Seki H, Koga Y*, et al.* (2006) Anomalous dynamic behavior of ions and water molecules in dilute aqueous solution of 1-butyl-3-methylimidazolium bromide studied by NMR. Chem. Phys. Lett.*,* 427(1), 87-90. https://doi.org/https://doi.org/10.1016/j.cplett.2006.06.052

**4.** Bhargava BL, & Klein ML (2009) Aqueous solutions of imidazolium ionic liquids: Molecular dynamics studies [10.1039/B908046E]. Soft Matter*,* 5(18), 3475-3480. https://doi.org/10.1039/B908046E

**5.** Sarraute S, Costa Gomes MF, & Pádua AAH (2009) Diffusion coefficients of 1-alkyl-3-methylimidazolium ionic liquids in water, methanol, and acetonitrile at infinite dilution. J. Chem. Eng. Data*,* 54(9), 2389-2394. https://doi.org/10.1021/je800817b

**6.** Soriano AN, Agapito AM, Lagumbay LJLI, Caparanga AR, & Li M-H (2011) Diffusion coefficients of aqueous ionic liquid solutions at infinite dilution determined from electrolytic conductivity measurements. J. Taiwan Inst. Chem. Eng.*,* 42(2), 258-264. https://doi.org/https://doi.org/10.1016/j.jtice.2010.06.003

**7.** Frisch MJ, Trucks GW, Schlegel HB, Scuseria GE, Robb MA*, et al.* (2016). *Gaussian 16 Rev. A.03.* In

**8.** Roothaan CCJ (1951) New developments in molecular orbital theory. Rev. Mod. Phys.*,* 23(2), 69-89. https://doi.org/10.1103/RevModPhys.23.69

**9.** Bayly CI, Cieplak P, Cornell W, & Kollman PA (1993) A well-behaved electrostatic potential based method using charge restraints for deriving atomic charges: The RESP model. J. Biophys. Chem.*,* 97(40), 10269-10280. https://doi.org/10.1021/j100142a004

**10.** Morrow TI, & Maginn EJ (2002) Molecular dynamics study of the ionic liquid 1-n-butyl-3-methylimidazolium hexafluorophosphate. J. Phys. Chem. B*,* 106(49), 12807-12813. https://doi.org/10.1021/jp0267003

**11.** Shekaari H, Zafarani-Moattar MT, Kazempour A, & Ghasedi-Khajeh Z (2015) Volumetric properties of aqueous ionic-liquid solutions at different temperatures. J. Chem. Eng. Data*,* 60(6), 1750-1755. https://doi.org/10.1021/je501161t

**12.** Sprenger KG, Jaeger VW, & Pfaendtner J (2015) The general AMBER force field (GAFF) can accurately predict thermodynamic and transport properties of many ionic liquids. J. Phys. Chem. B*,* 119(18), 5882-5895. https://doi.org/10.1021/acs.jpcb.5b00689

**13.** Salanne M (2015) Simulations of room temperature ionic liquids: From polarizable to coarse-grained force fields [10.1039/C4CP05550K]. Phys. Chem. Chem. Phys.*,* 17(22), 14270-14279. https://doi.org/10.1039/C4CP05550K

**14.** Schröder C (2012) Comparing reduced partial charge models with polarizable simulations of ionic liquids [10.1039/C2CP23329K]. Phys. Chem. Chem. Phys.*,* 14(9), 3089-3102. https://doi.org/10.1039/C2CP23329K

**15.** Schröder C, Lyons A, & Rick SW (2020) Polarizable MD simulations of ionic liquids: How does additional charge transfer change the dynamics? [10.1039/C9CP05478B]. Phys. Chem. Chem. Phys.*,* 22(2), 467-477. https://doi.org/10.1039/C9CP05478B

**16.** Yan T, Burnham CJ, Del Pópolo MG, & Voth GA (2004) Molecular dynamics simulation of ionic liquids: The effect of electronic polarizability. J. Phys. Chem. B*,* 108(32), 11877-11881. https://doi.org/10.1021/jp047619y

**17.** Izadi S, Anandakrishnan R, & Onufriev AV (2014) Building water models: A different approach. J. Phys. Chem. Lett.*,* 5(21), 3863-3871. https://doi.org/10.1021/jz501780a

**18.** Hollóczki O, Malberg F, Welton T, & Kirchner B (2014) On the origin of ionicity in ionic liquids: Ion pairing versus charge transfer [10.1039/C4CP01177E]. Phys. Chem. Chem. Phys.*,* 16(32), 16880-16890. https://doi.org/10.1039/C4CP01177E

**19.** Kawasaki K, Kondo H, Suzuki M, Ohgiya S, & Tsuda S (2002) Alternate conformations observed in catalytic serine of Bacillus subtilis lipase determined at 1.3 Å resolution. Acta Crystallogr., Sect. D: Struct. Biol., 58(7), 1168-1174. https://doi.org/10.1107/s090744490200714x

**20.** Zhao J, Frauenkron-Machedjou VJ, Fulton A, Zhu L, Davari MD, *et al.* (2018) Unraveling the effects of amino acid substitutions enhancing lipase resistance to an ionic liquid: a molecular dynamics study [10.1039/C7CP08470F]. Phys. Chem. Chem. Phys., 20(14), 9600-9609. https://doi.org/10.1039/C7CP08470F
